# A practical synthesis of long-chain iso-fatty acids (iso-C_12_–C_19_) and related natural products

**DOI:** 10.3762/bjoc.9.210

**Published:** 2013-09-04

**Authors:** Mark B Richardson, Spencer J Williams

**Affiliations:** 1 School of Chemistry, Bio21 Molecular Science and Biotechnology Institute, University of Melbourne, Parkville, Victoria 3010, Australia

**Keywords:** chemoselective reduction, Evans’ auxiliary, Grignard addition, homologation, ionic hydrogenation

## Abstract

A gram-scale synthesis of terminally-branched iso-fatty acids (iso-C_12_–C_19_) was developed commencing with methyl undec-10-enoate (methyl undecylenate) (for iso-C_12_–C_14_) or the C_15_ and C_16_ lactones pentadecanolide (for iso-C_15_–C_17_) and hexadecanolide (for iso-C_18_–C_19_). Central to the approaches outlined is the two-step construction of the terminal isopropyl group through addition of methylmagnesium bromide to the ester/lactones and selective reduction of the resulting tertiary alcohols. Thus, the C_12_, C_17_ and C_18_ iso-fatty acids were obtained in three steps from commercially-available starting materials, and the remaining C_13_–C_16_ and C_19_ iso-fatty acids were prepared by homologation or recursive dehomologations of these fatty acids or through intercepting appropriate intermediates. Highlighting the synthetic potential of the iso-fatty acids and various intermediates prepared herein, we describe the synthesis of the natural products (*S*)-2,15-dimethylpalmitic acid, (*S*)-2-hydroxy-15-methylpalmitic acid, and 2-oxo-14-methylpentadecane.

## Introduction

Long-chain iso-fatty acids occur in a broad range of organisms, and are especially abundant in bacteria where, through incorporation into phospholipids, they influence membrane fluidity [1]. Emerging evidence has revealed unexpected roles for certain iso-fatty acids; for example iso-C_15_ and iso-C_17_ fatty acids have been shown to be essential in the development of the model eukaryote *Caenorhabditis elegans* [2]. They are present as esters and amides in natural products including septacidin [3], teicoplanins [4], tunicaminyluracil-based antibiotics [5] (tunicamycins [6], corynetoxins [7], and streptovirudins [8]), the arylomycin glycopeptide antibiotics [9–10], maradolipids [11], plipastatin-type lipopeptides [12], Nod factors [13], glycosylglycerides [14–15], phosphoglycolipids [16], and various sphingolipids [17–19]. The terminal isopropyl group of the iso-fatty acids arises from valine and leucine, which through transamination and decarboxylation reactions yield isobutyryl-CoA and isovaleryl-CoA [20]. These starter units are elongated by fatty acid synthases to the final iso-fatty acids (even numbered for isobutyryl-CoA; odd-numbered for isovaleryl-CoA) through extension with malonyl-CoA [21–22]. Long-chain iso-fatty acids are important analytical reference compounds owing to the presence of these materials in tobacco [23], wool wax [24], butter fat [25], human sebaceous secretions [26] (adult skin [27], meibum [28], cerumen [29], and newborn vernix caseosa [30–31]), and a wide variety of microbiological samples [1].

**Figure 1:**
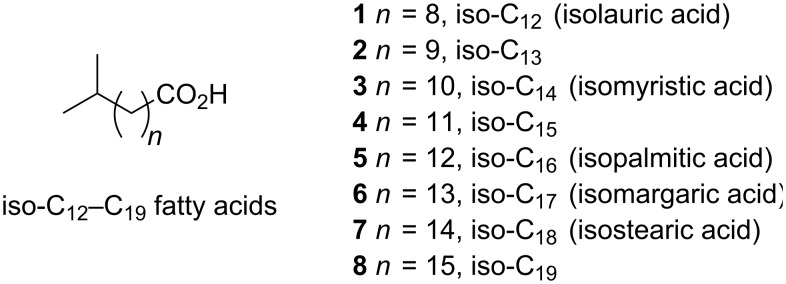
Structures of iso-fatty acids.

Previous syntheses of iso-fatty acids have typically utilized extended, multi-step sequences. Two main approaches have been used: (1) two-component cross-couplings that include α-ketoester alkylation/decarboxylation [32–33], aldehyde–olefin photoaddition [34], acetylide alkylation (sp^3^–sp) [35–36], Wittig coupling [3,21,37–39], Kolbe electrosynthesis [35,40–42], organocadmium (sp^2^–sp^3^) [43–46], organomagnesium (sp^2^–sp^3^) [47], or organocopper (sp^3^–sp^3^) [48–49] cross-couplings; or (2) bidirectional extension of a central thiophene C_4_-fragment [50]. Two fundamentally different approaches worth special mention are the synthesis of the iso-C_14_ acid **3** by direct hydro-isopropylation of the terminal alkene of methyl undecylenate (available as a pyrolysis product of ricinoleic acid) using isopropyl chloroformate and ethyldichloroaluminium [51], and the synthesis of the iso-C_17_ acid **6** from methyl ustilate (15,16-dihydroxypalmitate) [52]. Despite the interest in natural products containing iso-fatty acids, these compounds are not readily acquired in multigram quantities due to the complexity of the synthetic routes or limited availability of starting materials. To overcome these problems, we report the scalable, gram-scale syntheses of eight common iso-C_12_–C_19_ acids **1**–**8** (Figure 1), from readily available starting materials. To illustrate the opportunities that our approach provides, we demonstrate the elaboration of the C_17_-iso-fatty acid **6** and an intermediate, **22**, to several terminal-branched natural products that have not previously been synthesized.

## Results and Discussion

**Scheme 1:**
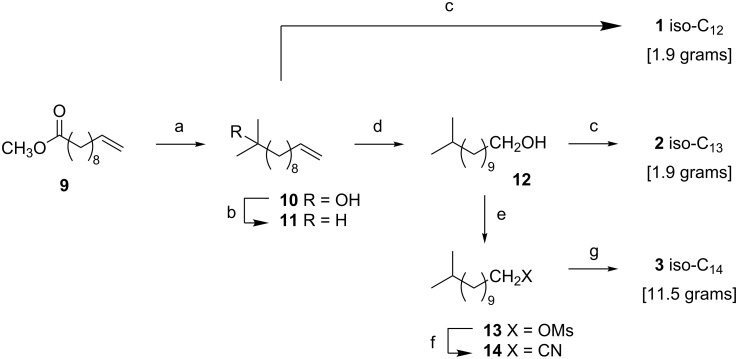
Synthesis of iso-C_12_ **1**, iso-C_13_ **2**, and iso-C_14_ **3** fatty acids from methyl undecylenate (**9**). Reagents and conditions: (a) MeMgBr, THF, 98%; (b) BF_3_·Et_2_O, Et_3_SiH, CH_2_Cl_2_, 99%; (c) KMnO_4_, Bu_4_NBr, AcOH, H_2_O, 88% for **1**, 96% for **2**; (d) i) I_2_, NaBH_4_, THF, ii) H_2_O_2_, 95%; (e) MsCl, Et_3_N, CH_2_Cl_2_, 98%; (f) KCN, DMSO, THF, 72%; (g) aq NaOH, EtOH, 96%.

Our approach to the iso-C_12_–C_14_ fatty acids **1**–**3** commenced from methyl undec-10-enoate (methyl undecylenate) **9**. Reaction of **9** with methylmagnesium bromide afforded the tertiary alcohol **10** in 98% yield (Scheme 1). Selective reduction of the tertiary alcohol of **10** was achieved by ‘ionic hydrogenation’ with triethylsilane and BF_3_·Et_2_O [53], affording **11**. Oxidative cleavage of **11** with KMnO_4_/Bu_4_NBr [54] afforded iso-C_12_ acid **1**. Alternatively, anti-Markovnikov hydration of **11**, using I_2_/NaBH_4_ then hydrogen peroxide [55], afforded the alcohol **12**, and oxidation of **12** with KMnO_4_/Bu_4_NBr afforded iso-C_13_ acid **2**. Alternatively, alcohol **12** could be intercepted and converted to the mesylate **13** using MsCl/Et_3_N [56] and thence the nitrile **14** (KCN in DMSO/THF). Finally, hydrolysis of the nitrile **14** with NaOH in H_2_O/EtOH afforded iso-C_14_ acid **3**.

**Scheme 2:**
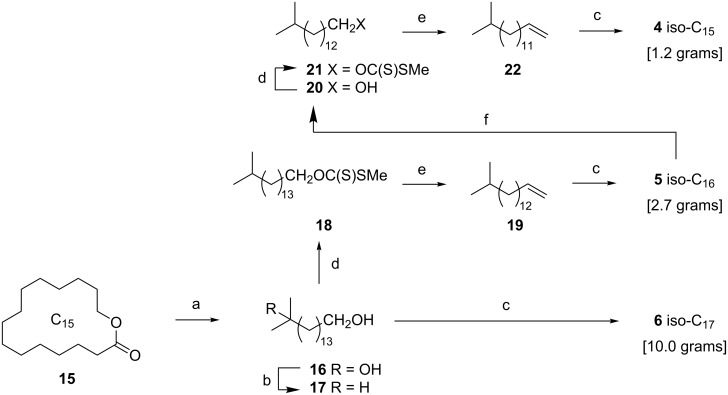
Synthesis of iso-C_15_ **4**, iso-C_16_ **5**, and iso-C_17_ **6** fatty acids from pentadecanolide (**15**). Reagents and conditions: (a) MeMgBr, THF, 98%; (b) BF_3_·Et_2_O, Et_3_SiH, CH_2_Cl_2_, 96%; (c) KMnO_4_, Bu_4_NBr, AcOH, H_2_O, 95% for **6**, 93% for **5**; 86% for **4**; (d) NaH, CS_2_ then MeI; (e) reflux, 89% for **19** over 2 steps from **17**, 98% for **22** over 2 steps from **20**; (f) BH_3_·SMe_2_, THF, 78%.

The iso-C_15_–C_17_ fatty acids **4**–**6** were prepared from the readily available C_15_ lactone pentadecanolide (exaltolide, **15**) [57], a natural product that is produced industrially for use as a musk-odored perfumery fixative. Reaction of **15** with methylmagnesium bromide afforded the tertiary alcohol **16** in 98% yield (Scheme 2). Selective reduction of the tertiary alcohol of **16** was achieved using triethylsilane/BF_3_·Et_2_O [53], yielding **17**. Finally, oxidation of **17** with KMnO_4_/Bu_4_NBr [54] afforded the iso-C_17_ acid **6**. The iso-C_15_ acid **4** and iso-C_16_ acid **5** were prepared by recursive dehomologation through intercepting the alcohol **17**. Preparation of the xanthate ester **18** (NaH, CS_2_, then MeI) [58] followed by Chugaev elimination afforded the terminal alkene **19**. Oxidative cleavage of **19** using KMnO_4_/Bu_4_NBr [54] afforded iso-C_16_ acid **5**. Reduction of **5** (BH_3_·Me_2_S) [59] afforded the alcohol **20** that when subjected to the same transformations as before, via the xanthate ester **21**, delivered the terminal alkene **22**. Finally, oxidative cleavage (KMnO_4_/Bu_4_NBr) [54] of **22** afforded iso-C_15_ acid **4**.

**Scheme 3:**
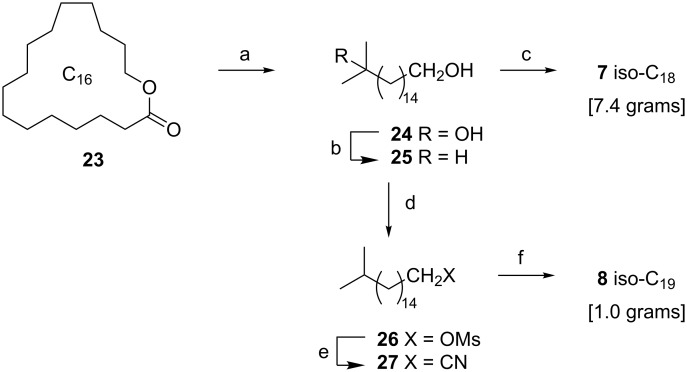
Synthesis of iso-C_18_ **7** and iso-C_19_ **8** fatty acids from hexadecanolide **23**. Reagents and conditions: (a) MeMgBr, THF, 97%; (b) BF_3_·Et_2_O, Et_3_SiH, CH_2_Cl_2_, 95%; (c) KMnO_4_, Bu_4_NBr, AcOH, H_2_O, 82%; (d) MsCl, Et_3_N, 96%; (e) KCN, DMSO, THF, 75%; (f) aq NaOH, EtOH, 86%.

The iso-C_18_ **7** and iso-C_19_ **8** fatty acids were synthesized through similar approaches from the related C_16_ lactone hexadecanolide **23** [60] (Scheme 3). Reaction of **23** with methylmagnesium bromide afforded **24**; triethylsilane/BF_3_·Et_2_O [53] reduction gave **25**; and KMnO_4_/Bu_4_NBr oxidation afforded iso-C_18_ acid **7** (Scheme 3). The iso-C_19_ acid **8** was readily prepared by a three-step homologation through intercepting the alcohol **25**. Thus, mesylation of **25** (MsCl/Et_3_N [56]) afforded **26**; substitution (KCN/DMSO) afforded the nitrile **27**; and hydrolysis (NaOH in H_2_O/EtOH) of **27** afforded **8**.

**Scheme 4:**
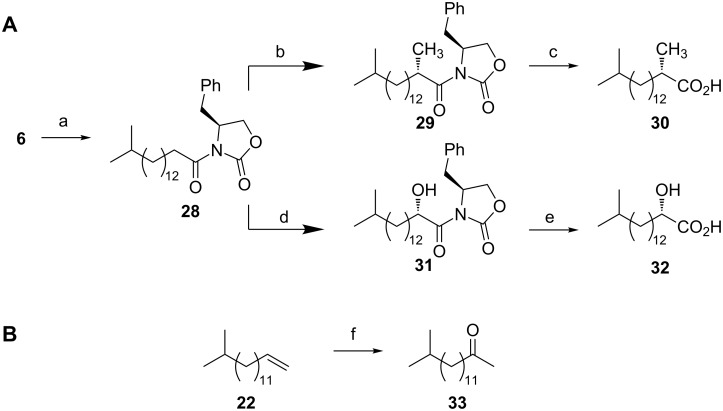
Synthesis of (A) 2-methyl- and 2-hydroxy-iso-fatty acids **30** and **32**, and (B) the ketone **33**. Reagents and conditions: (a) Et_3_N, PivCl, LiCl, DMAP, (*S*)-4-benzyloxazolidinone, 71%; (b) NaHMDS, MeI, THF, 80%; (c) LiOH, H_2_O_2_, THF, H_2_O, 98%; (d) NaHMDS, Davis oxaziridine, THF, 71%; (e) i) iPrMgCl, MeOH, 76%, ii) NaOH, MeOH, 83%; (f) O_2_, PdCl_2_, DMA, H_2_O, 51%.

The above routes enable the acquisition of (multi)gram quantities of the iso-C_12–19_ acids **1**–**8**, and provide opportunities for their use as starting materials for the preparation of more complex fatty acids. To illustrate their potential we undertook the synthesis of several representative natural products (Scheme 4). 2,15-Dimethylpalmitic acid has been isolated from a microaerophilic subsurface microbial community [61], and is a component of human newborn vernix caseosa [31], although the absolute configuration of natural samples has not been determined. Conversion of iso-C_17_ acid **6** to the *N*-acyloxazolidinone **28** was achieved using pivalyl chloride/LiCl [62] and (*S*)-4-benzyloxazolidinone. Diastereoselective methylation [63] of the chelated *Z*-enolate, derived from deprotonation of **28**, using NaHMDS, followed by addition of iodomethane, yielded **29** as a single diastereoisomer (as determined by ^1^H NMR) in 80% yield. Cleavage of the chiral auxiliary using LiOH/H_2_O_2_ (which occurs without racemization at the α-position) [64] afforded (*S*)-2,15-dimethylpalmitic acid (**30**) in 98% yield. 2-Hydroxy-15-methylpalmitic acid has been identified from a range of sources [1] including the myxobacterium *Stigmatella aurantiaca* [21,65], and the oral bacterium *Veillonella parvula* [66], although the absolute configuration has not been reported. Diastereoselective hydroxylation [67] of the chelated *Z*-enolate derived from **28** using the Davis oxaziridine [68] afforded the 2-hydroxy compound **31** as a single diastereoisomer (as determined by ^1^H NMR) in 71% yield. Esterification with MeOMgCl [69] (which has been shown not to cause epimerization at the α-position [67]) and saponification [70] afforded (*S*)-2-hydroxy-15-methylpalmitic acid (**32**). The ketone **33** was isolated from *Xanthomonas campestris* pv. *vesicatoria* 85-10 [71]. A direct one step synthesis of **33** was achieved in 51% yield by Wacker oxidation using Pd/O_2_ [72] of the alkene **22**, intercepted from the synthesis of the iso-C_15_ acid **4**.

## Conclusion

We have accomplished a highly practical synthesis of the homologous iso-fatty acids **1**–**8**. The iso-C_12_ **1**, iso-C_17_ **6** and iso-C_18_ **7** acids were prepared from commercially-available starting materials through three-step sequences and produced more than 1 g of each of the three iso-fatty acids in just 2 days each. The remaining five fatty acids were each prepared on >1 g scale by homologation or dehomologation reactions, or through the elaboration of intermediates in the synthesis of **1**. Underscoring the practicability of this approach, the iso-fatty acids or appropriate intermediates were used for the preparation of three natural products, enantiopure acids **30** and **32**, and the ketone **33**. The simplicity of our approach suggests that it will be of great utility in the preparation of iso-fatty acids for incorporation into more complex molecules.

## Supporting Information

File 1 Experimental part.

File 2 NMR spectra.
