# Supplementary material for: A practical synthesis of long-chain iso-fatty acids (iso-C12–C19) and related natural products
Source: Beilstein J Org Chem. 2013 Sep 4;9:1807–12. doi: 10.3762/bjoc.9.210 (PMC3778388; doi:10.3762/bjoc.9.210)
Supplement: File 1 — Experimental part. [file Beilstein_J_Org_Chem-09-1807-s001.pdf]

Supporting Information

for

A practical synthesis of long-chain iso-fatty acids (iso-C_{12}–C_{19}) and related natural products

Mark B. Richardson and Spencer J. Williams*

Address: School of Chemistry, Bio21 Molecular Science and Biotechnology Institute, University of Melbourne, Parkville, Victoria 3010, Australia

Email: Spencer J. Williams - sjwill@unimelb.edu.au

* Corresponding author

Experimental part

Table of Contents

Experimental S3

General Experimental Methods. S3

11-Hydroxy-11-methyl-dodec-1-ene (**10**)....S3

11-Methyldodec-1-ene (**11**). S4

10-Methylundecanoic acid (**1; iso-C_{12} acid**). S4

11-Methyldodecan-1-ol (**12**). S5

11-Methyldodecanoic acid (**2; iso-C_{13} acid**). S6

11-Methyldodecyl methanesulfonate (**13**). S6

12-Methyltridecanenitrile (**14**). S7

12-Methyltridecanoic acid (**3; iso-C_{14} acid**)....S7

15-Methylhexadecane-1,15-diol (**16**)....S8

15-Methylhexadecan-1-ol (**17**). S8

15-Methylhexadecanoic acid (**6; iso-C_{17} acid**)....S9

15-Methylhexadec-1-ene (**19**)....S9
14-Methylpentadecanoic acid (**5; iso-C_{16} acid**)....S11
14-Methylpentadecan-1-ol (**20**). S11
14-Methylpentadecan-1-ene (**22**)....S12
13-Methyltetradecanoic acid (**4; iso-C_{15} acid**). S12
16-Methylheptadecane-1,16-diol (**24**). S12
16-Methylheptadecan-1-ol (**25**). S13
16-Methylheptadecanoic acid (**7; iso-C_{18} acid**)....S13
16-Methylheptadecyl methanesulfonate (**26**)....S13
17-Methyloctadecanenitrile (**27**)....S14
17-Methyloctadecanoic acid (**8; iso-C_{19} acid**)....S14
(*S*)-4-Benzyl-*N*-(15-methylhexadecanoyl)oxazolidin-2-one (**28**)....S14
(*S*)-2,15-Dimethylhexadecanoic acid (**30**). S15
3-Phenyl-2-(phenylsulfonyl)-1,2-oxaziridine. S16
(*S*)-2-Hydroxy-15-methylhexadecanoic acid (**32**). S17
14-Methylpentadecan-2-one (**33**)....S19
References....S19

Experimental

General experimental methods

Proton nuclear magnetic resonance spectra (^1H NMR, 500 MHz) and proton-decoupled carbon nuclear magnetic resonance spectra (^{13}C NMR, 125 MHz) were obtained in deuterochloroform, methanol-d_4 (CD_3OD) and DMSO-d_6 with residual protonated solvent as internal standard. Abbreviations for multiplicity are s, singlet; d, doublet; t, triplet; q, quartet; p, pentet. Fourier-transform infrared spectra were obtained as neat samples on an attenuated total reflectance instrument using a diamond-coated zinc selenide sample accessory. Flash chromatography was carried out on silica gel 60 according to the procedure of Still et al. [1] Analytical thin layer chromatography (tlc) was conducted on aluminium-backed 2 mm thick silica gel 60 GF_{254} and chromatograms were visualized with ceric ammonium molybdate (Hanessian's stain) or potassium permanganate. High resolution mass spectra (HRMS) were obtained by ionizing samples using electrospray ionization (ESI) and a time-of-flight mass analyzer. Dry DMF was obtained by drying over 4 Å molecular sieves. Hexanes refers to petroleum ether, boiling range 40–60 °C. Dichloromethane and THF were dried over alumina according to the method of Pangborn et al [2].

11-Hydroxy-11-methyl-dodec-1-ene (10)

Methylmagnesium bromide (3.0 M in THF, 101 mL, 303 mmol) was added dropwise to a solution of methyl undecylenate **9** (20.0 g, 101 mmol) in dry THF (200 mL) at –78 °C under nitrogen. After complete addition the reaction mixture was warmed to rt and stirred vigorously under nitrogen for 20 h. The grey solution was cooled to 0 °C, then acidified by slow addition of cold 3.0 M aq HCl (100 mL). The mixture was concentrated in vacuo, then diluted into water and extracted with Et_2O (4 × 75 mL). The combined organic phases were washed with brine (2 × 150 mL), then dried ($MgSO_4$), filtered and evaporated in vacuo to give a brown liquid. Purification by flash chromatography (Et_2O/hexanes, 20 → 50%) afforded **10** as a colorless liquid (19.6 g, 98%); ^1H NMR (500 MHz, $CDCl_3$) δ 1.20 (6H, s), 1.27-1.41 (13H, m), 1.42-1.48 (2H, m), 2.00-2.06 (2H,

m), 4.90-4.94 (1H, m), 4.96-5.01 (1H, m), 5.80 (1H, ddt, J = 17.1, 10.3, 6.7 Hz); ^{13}C NMR (125 MHz, $CDCl_3$) δ 24.5, 29.0, 29.2, 29.4, 29.6, 29.7, 30.3, 33.9, 44.1, 71.2, 114.2, 139.3; IR υ 907.7, 2854, 2926 cm^{-1}; HRMS (ESI$^+$) calcd for $C_{13}H_{26}NaO$ $[M + Na]^+$ m/z 221.1876, found 221.1876.

11-Methyldodec-1-ene (11)

BF_3·Et_2O (24.7 mL, 222 mmol) was added to a solution of **10** (22.0 g, 111 mmol) and triethylsilane (35.4 mL, 222 mmol) in dry CH_2Cl_2 (222 mL) at 0 °C, then the reaction mixture was warmed to rt and stirred under nitrogen for 5 min. The reaction mixture was cooled on an ice bath, then diluted with ice water (500 mL) and extracted with CH_2Cl_2 (3 × 150 mL). The combined organic phases were washed with sat. aq $NaHCO_3$ (2 × 100 mL), then dried ($MgSO_4$), filtered and evaporated in vacuo to give a yellow liquid. Purification by flash chromatography (Et_2O/hexanes, 2 → 10%) afforded **11** as a colorless liquid (20.2 g, 99%); ^{1}H NMR (500 MHz, $CDCl_3$) δ 0.87 (6H, d, J = 6.6 Hz), 1.13-1.20 (2H, m), 1.22-1.35 (12H, m), 1.36-1.43 (2H, m), 1.53 (1H, t of sept, J = 6.6, 6.6 Hz), 2.02-2.08 (2H, m), 4.92-4.95 (1H, m), 4.98-5.02 (1H, m), 5.82 (1H, ddt, J = 17.0, 10.3, 6.7 Hz); ^{13}C NMR (125 MHz, $CDCl_3$) δ 22.8, 27.6, 28.2, 29.1, 29.3, 29.7, 29.8, 30.1, 34.0, 39.2, 114.2, 139.4; IR υ 908.6, 2854, 2924 cm^{-1}; HRMS (ESI$^+$) calcd for $C_{13}H_{26}I$ $[M + I]^+$ m/z 309.1074, found 309.1087.

10-Methylundecanoic acid (1; iso-C_{12} acid)

A two-phase mixture of **11** (2.00 g, 11.0 mmol), tetrabutylammonium bromide (1.92 g, 5.95 mmol), $KMnO_4$ (17.3 g, 110 mmol), AcOH (27 mL), CH_2Cl_2 (110 mL) and water (110 mL) was heated to reflux with vigorous stirring for 36 h. The resulting dark brown and flocculent reaction mixture was cooled to rt, then acidified by addition of 5 M aq HCl (132 mL). Na_2SO_3 (19.4 g, 154 mmol) was added carefully in small portions with vigorous stirring until all the brown sediment had disappeared. The organic phase was collected and the remaining aqueous phase was extracted with

CH_2Cl_2 (2 × 150 mL). The combined organic phases were dried ($MgSO_4$), filtered and evaporated in vacuo to give a brown liquid. Purification by flash chromatography (hexanes/EtOAc/AcOH, 78:20:2) afforded **1** as a lustrous white solid (1.94 g, 88%); mp 40–41 °C (lit. [3] mp 41.2 °C); ^{1}H NMR (500 MHz, $CDCl_3$) δ 0.87 (6H, d, *J* = 6.7 Hz), 1.15 (2H, m), 1.23-1.39 (10H, m), 1.52 (1H, t of sept, *J* = 6.6, 6.6 Hz), 1.64 (2H, tt, *J* = 7.5, 7.5 Hz), 2.36 (2H, t, *J* = 7.5 Hz); ^{13}C NMR (125 MHz, $CDCl_3$) δ 22.8, 24.8, 27.5, 28.1, 29.2, 29.4, 29.6, 30.0, 34.2, 39.2, 180.2; IR υ 1708, 2855, 2924 cm^{-1}; HRMS (ESI^-) calcd for $C_{12}H_{23}O_2$ $[M – H]^-$ *m/z* 199.1704, found 199.1707.

11-Methyldodecan-1-ol (12)

A solution of I_2 (13.8 g, 54.3 mmol) in dry THF (148 mL) was added to a solution of $NaBH_4$ (1.87 g, 49.4 mmol) in THF (148 mL) and the brown solution was heated at reflux under a dry nitrogen atmosphere for 40 min, and the resulting cloudy and colorless solution was cooled to 0 °C. A solution of **11** (18.0 g, 98.7 mmol) in dry THF (10.0 mL) was added and the resulting reaction mixture was warmed to rt and stirred under nitrogen for 3 h. The reaction mixture was cooled to 0 °C, then 30% aq H_2O_2/3 N aq NaOH (1:1, 8 × 50 mL aliquots) were separately added to the reaction mixture, with each addition resulting in a strongly exothermic reaction and production of a large volume of gas. After cooling, the mixture was concentrated in vacuo, then diluted into water and extracted with Et_2O (4 × 125 mL). The combined organic phases were washed with brine (2 × 150 mL), then dried ($MgSO_4$), filtered and evaporated in vacuo to give a yellow liquid. Purification by flash chromatography (Et_2O/hexanes, 20 → 40%) afforded **12** as a colorless liquid (18.8 g, 95%); ^{1}H NMR (500 MHz, $CDCl_3$) δ 0.86 (6H, d, *J* = 6.6 Hz), 1.10-1.18 (2H, m), 1.21-1.38 (15H, m), 1.46-1.59 (3H, m), 3.63 (2H, t, *J* = 6.7 Hz); ^{13}C NMR (125 MHz, $CDCl_3$) δ 22.8, 25.9, 27.6, 28.1, 29.6, 29.8, 29.8, 29.8, 30.1, 32.9, 39.2, 63.2; IR υ 1466, 2853, 2923 cm^{-1}; HRMS (ESI^+) calcd for $C_{13}H_{28}NaO$ $[M + Na]^+$ *m/z* 223.2032, found 223.2032.

11-Methyldodecanoic acid (2; iso-C_{13} acid)

A mixture of **12** (3.00 g, 15.0 mmol), tetrabutylammonium bromide (2.62 g, 8.12 mmol), $KMnO_4$ (7.38 g, 46.7 mmol), AcOH (23.0 mL), CH_2Cl_2 (180 mL) and water (60 mL) was heated under reflux with vigorous stirring for 24 h. The resulting dark brown flocculent reaction mixture was cooled to rt, then acidified by addition of 5 M aq HCl (180 mL). Na_2SO_3 (8.34 g, 66.2 mmol) was added carefully in small portions with vigorous stirring until all the brown sediment dissolved. The organic phase was collected and the aqueous phase was extracted with CH_2Cl_2 (2 × 100 mL). The combined organic phases were dried ($MgSO_4$), filtered and evaporated in vacuo to give a yellow liquid. Purification by flash chromatography (hexanes/EtOAc/AcOH, 78:20:2) afforded **2** as a lustrous white solid (2.87 g, 96%); mp 40–41 °C (lit. [4] mp 41.3 °C); ^{1}H NMR (500 MHz, $CDCl_3$) δ 0.87 (6H, d, *J* = 6.6 Hz), 1.12-1.18 (2H, m), 1.24-1.39 (12H, m), 1.52 (1H, t of sept, *J* = 6.6, 6.6 Hz), 1.64 (2H, tt, J = 7.5, 7.5 Hz), 2.35 (2H, t, *J* = 7.5 Hz); ^{13}C NMR (125 MHz, $CDCl_3$) δ 22.8, 24.8, 27.5, 28.1, 29.2, 29.4, 29.6, 29.8, 30.0, 34.2, 39.2, 180.1; IR υ 1707, 2855, 2925 cm^{-1}; HRMS (ESI$^-$) calcd for $C_{13}H_{25}O_2$ $[M – H]^-$ *m/z* 213.1860, found 213.1862.

11-Methyldodecyl methanesulfonate (13)

MsCl (6.95 mL, 89.8 mmol) was added dropwise to a stirred solution of **12** (15.0 g, 74.9 mmol) and Et_3N (15.7 mL, 112 mmol) in dry CH_2Cl_2 (375 mL) at −10 °C with stirring under nitrogen. After 15 min the reaction mixture was diluted with ice-water (300 mL) and extracted with CH_2Cl_2 (4 × 75 mL). The combined organic phases were washed sequentially with cold 10% aq HCl (100 mL), $NaHCO_3$ (100 mL), brine (100 mL), dried ($MgSO_4$), filtered and evaporated in vacuo to give a yellow liquid. Purification by flash chromatography (Et_2O/hexanes, 10 → 40%) afforded **13** as a colorless liquid (20.5 g, 98%); ^{1}H NMR (500 MHz, $CDCl_3$) δ 0.85 (6H, d, *J* = 6.6 Hz), 1.10-1.18 (2H, m), 1.19-1.35 (12H, m), 1.35-1.42 (2H, m), 1.50 (1H, t of sept, *J* = 6.6, 6.6 Hz), 1.70-1.76 (2H, m), 2.99 (3H, s), 4.21 (2H, t, *J* = 6.6 Hz); ^{13}C NMR (125 MHz, $CDCl_3$) δ 22.8, 25.5, 27.5, 28.1,

29.1, 29.2, 29.5, 29.6, 29.7, 30.0, 37.4, 39.1, 70.3; IR υ 1173, 1352, 2854, 2924 cm^{-1}; HRMS (ESI^+) calcd for $C_{14}H_{30}NaO_3S$ $[M + Na]^+$ *m/z* 301.1808, found 301.1808.

12-Methyltridecanenitrile (14)

A mixture of **13** (20.5 g, 73.7 mmol), KCN (6.00 g, 92.1 mmol), dry DMSO (221 mL) and dry THF (221 mL) was stirred at 85 °C under nitrogen for 24 h. The bright yellow reaction mixture was evaporated in vacuo, and the residue was dissolved in a mixture of Et_2O (100 mL) and water (100 mL). The organic phase was separated and the aqueous phase was extracted with Et_2O (3 × 50 mL). The combined organic phases were washed with brine (2 × 200 mL), dried ($MgSO_4$), filtered and evaporated in vacuo to give a dark red liquid. Purification by flash chromatography (Et_2O/hexanes, 10 → 20%) afforded **14** as a bright yellow liquid (11.1 g, 72%); ^1H NMR (500 MHz, $CDCl_3$) δ 0.86 (6H, d, *J* = 6.6 Hz), 1.11-1.18 (2H, m), 1.22-1.36 (12H, m), 1.40-1.48 (2H, m), 1.51 (1H, t of sept, *J* = 6.6, 6.6 Hz), 1.62-1.68 (2H, m), 2.33 (2H, t, *J* = 7.2 Hz); ^{13}C NMR (125 MHz, $CDCl_3$) δ 17.2, 22.8, 25.5, 27.5, 28.1, 28.8, 28.9, 29.4, 29.6, 29.7, 30.0, 39.2, 120.0; IR υ 1466, 2247, 2854, 2923 cm^{-1}; HRMS (ESI^+) calcd for $C_{14}H_{28}N$ $[M + H]^+$ *m/z* 210.2216, found 210.2216.

12-Methyltridecanoic acid (3; iso-C_{14} acid)

8 N aq NaOH solution (200 mL) was added to a solution of **14** (11.0 g, 52.5 mmol) in EtOH (200 mL), and the resulting cloudy reaction mixture was stirred at reflux under nitrogen for 48 h. The resulting bright yellow, biphasic mixture was then cooled to rt and a white precipitate appeared. The reaction mixture was diluted with water (250 mL) and then acidified to pH < 2 by careful addition of conc HCl at 0 °C. The mixture was extracted with Et_2O (4 × 75 mL) and the combined organic phases were dried ($MgSO_4$), filtered and evaporated in vacuo. The residue was crystallized from acetone at –20 °C, affording **3** as a lustrous white solid with a strong odor (11.5 g, 96%); mp 53−54 °C (lit. [3] mp 53.3 °C); ^1H NMR (500 MHz, $CDCl_3$) δ 0.87 (6H, d, *J* = 6.6 Hz), 1.12-1.18

(2H, m), 1.20-1.40 (14H, m), 1.52 (1H, t of sept, *J* = 6.6, 6.6 Hz), 1.64 (2H, tt, *J* = 7.4, 7.4 Hz), 2.35 (2H, t, *J* = 7.4 Hz); ^{13}C NMR (125 MHz, $CDCl_3$) δ 22.8, 24.8, 27.6, 28.1, 29.2, 29.4, 29.6, 29.7, 29.8, 30.1, 34.2, 39.2, 180.0; IR υ 1697, 2852, 2917 cm^{-1}; HRMS (ESI$^-$) calcd for $C_{14}H_{27}O_2$ [M – H]$^-$ *m/z* 227.2017, found 227.2024.

15-Methylhexadecane-1,15-diol (16)

Methylmagnesium bromide (3.0 M in THF, 83.2 mL, 250 mmol) was added dropwise to a solution of 15-pentadecanolide **15** (20.0 g, 83.2 mmol) in dry THF (166 mL) at –78 °C under nitrogen. After complete addition the reaction mixture was warmed to rt and stirred vigorously under nitrogen for 16 h. Ice water (500 mL) was carefully added to the resulting solid reaction mixture and the solid was broken up with a glass rod. The suspension was diluted with CH_2Cl_2 (500 mL) and acidified by addition of AcOH until the suspension had dissolved. The organic phase was collected and the remaining aqueous phase was extracted with CH_2Cl_2 (2 × 100 mL). The combined organic phases were washed with brine (150 mL), then dried ($MgSO_4$), filtered and evaporated in vacuo to give a yellow solid. The solid was crystallized from 9:1 hexanes/EtOAc to afford **16** as a lustrous white solid (22.1 g, 98%); mp 68−69 °C (lit. [5] mp 62 °C); ^{1}H NMR (500 MHz, DMSO-d_6) δ 1.04 (6H, s), 1.20-1.35 (24H, m), 1.35-1.43 (2H, m), 3.33-3.39 (2H, m), 4.03 (1H, s), 4.31 (1H, t, *J* = 5.2 Hz); ^{13}C NMR (125 MHz, DMSO-d_6) δ 23.9, 25.5, 28.9, 29.01, 29.02, 29.03 (2C), 29.09, 29.12, 29.25, 29.26, 29.8, 32.5, 43.7, 60.7, 68.7; HRMS (ESI$^+$) calcd for $C_{17}H_{36}NaO_2$ [M + Na]$^+$ *m/z* 295.2608, found 295.2614.

15-Methylhexadecan-1-ol (17)

$BF_3 \cdot Et_2O$ (89.5 mL, 804 mmol) was added to a solution of **16** (21.9 g, 80.4 mmol) and triethylsilane (25.7 mL, 161 mmol) in dry CH_2Cl_2 (160 mL) at 0 °C, then the reaction mixture was warmed to rt and stirred under nitrogen for 45 min. The reaction mixture was diluted with ice-water (300 mL) and extracted with CH_2Cl_2 (3 × 150 mL). The combined organic phases were washed with sat. aq

$NaHCO_3$ (2 × 100 mL), then dried ($MgSO_4$), filtered and evaporated in vacuo to give a yellow liquid. The liquid was crystallized from 9:1 hexanes/EtOAc affording **17** as a lustrous white solid (19.8 g, 96%); mp 39−40 °C (lit. [6] mp 42−44 °C; ^{1}H NMR (500 MHz, $CDCl_3$) δ 0.87 (6H, d, *J* = 6.6 Hz), 1.11-1.18 (2H, m), 1.24-1.40 (23H, m), 1.48-1.60 (3H, m), 3.64 (2H, td, *J* = 6.6, 5.2 Hz); ^{13}C NMR (125 MHz, $CDCl_3$) δ 22.8, 25.9, 27.6, 28.1, 29.6, 29.75, 29.77, 29.81, 29.83, 29.84, 29.85, 29.9, 30.1, 33.0, 39.2, 63.3; HRMS (ESI^+) calcd for $C_{17}H_{36}NaO$ $[M + Na]^+$ *m/z* 279.2658, found 279.2666.

15-Methylhexadecanoic acid (6; iso-C_{17} acid)

Compound **17** (10.0 g, 39.0 mmol) was subjected to the same conditions as compound **12** (in the preparation of **2**). Following work-up and evaporation of solvent the residue was crystallized from acetone at −20 °C to afford **6** as a lustrous white solid with a strong odor (10.0 g, 95%); mp 58−60 °C (lit. [3] mp 60.2 °C); ^{1}H NMR (500 MHz, $CDCl_3$) δ 0.87 (6H, d, *J* = 6.6 Hz), 1.12-1.18 (2H, m), 1.24-1.39 (20H, m), 1.52 (1H, t of sept, J = 6.6, 6.6 Hz), 1.64 (2H, tt, J = 7.5, 7.5 Hz), 2.35 (2H, t, *J* = 7.5 Hz); ^{13}C NMR (125 MHz, $CDCl_3$) δ 22.8, 24.9, 27.6, 28.1, 29.2, 29.4, 29.6, 29.7, 29.79, 29.82, 29.84, 29.9, 30.10, 34.14, 39.2, 179.5; IR υ 1698, 2850, 2919 cm^{-1}; HRMS (ESI^-) calcd for $C_{17}H_{33}O_2$ $[M - H]^-$ *m/z* 269.2486, found 269.2480.

15-Methylhexadec-1-ene (19)

(a) *S*-Methyl-*O*-15-methylhexadecyl dithiocarbonate (**18**). Sodium hydride (60% in mineral oil, 3.61 g, 90.2 mmol) was added in one portion to a solution of **17** (9.25 g, 36.1 mmol) in dry THF (115 mL) under nitrogen at 0 °C, and the mixture was stirred at rt for 1 h. CS_2 (3.25 mL, 54.1 mmol) in dry THF (5 mL) was added to the resulting white suspension at 0 °C, and the mixture was stirred at rt for 1 h. MeI (3.37 mL, 54.1 mmol) in dry THF (5 mL) was added to the resulting yellow suspension at 0 °C and the mixture was stirred at rt for 5 h. The bright yellow reaction mixture was then diluted with sat. NH_4Cl (120 mL) and extracted with EtOAc (3 × 150 mL). The

combined organic phases were dried ($MgSO_4$), filtered and evaporated in vacuo to give an orange liquid. The liquid was partially purified by passage through a short silica plug (hexanes/EtOAc, 95%). Evaporation of the eluant afforded crude **18** (16.0 g) as a bright yellow liquid that was used immediately without further purification; ^1H NMR (500 MHz, $CDCl_3$) δ 0.87 (6H, d, *J* = 6.6 Hz), 1.12-1.19 (2H, m), 1.24-1.36 (20H, m), 1.37-1.44 (2H, m), 1.52 (1H, t of sept, *J* = 6.6, 6.6 Hz), 1.80 (2H, tt, J = 7.1, 7.1 Hz), 2.56 (3H, s), 4.60 (2H, t, *J* = 6.7 Hz); ^{13}C NMR (125 MHz, $CDCl_3$) δ 19.0, 22.8, 26.0, 27.6, 28.1, 28.4, 29.4, 29.6, 29.7, 29.78, 29.81, 29.83, 29.84, 29.9, 30.1, 39.2, 74.4, 216.1.

(b) 15-Methylhexadec-1-ene (**19**). A 500 mL flask equipped with a reflux condenser, nitrogen line, and bleach trap, was charged with the crude dithiocarbonate ester **18** (16.0 g). The flask was heated over a Bunsen flame under a flow of nitrogen for 5 min resulting in deposition of a small amount of black powder in the flask. During this time, gas was vigorously evolved and was carried by the nitrogen flow through the bleach trap. After cooling, the reflux condenser was rinsed with hexanes back into the flask, and the collected washings were evaporated in vacuo. The heating and rinsing procedure was repeated two more times with the residue, then the reflux condenser was replaced with a short-path distillation head and the residue was distilled under reduced pressure. The bright yellow and foul smelling distillate was further purified by flash chromatography (100% hexanes), affording **19** as a colorless and odorless liquid (7.65 g, 89% over two steps); ^1H NMR (500 MHz, $CDCl_3$) δ 0.87 (6H, d, *J* = 6.6 Hz), 1.12-1.19 (2H, m), 1.24-1.32 (18H, m), 1.34-1.42 (2H, m), 1.53 (1H, t of sept, *J* = 6.6, 6.6 Hz), 2.02-2.08 (2H, m), 4.92-4.96 (1H, m), 4.97-5.03 (1H, m), 5.82 (1H, ddt, *J* = 17.0, 10.3, 6.7 Hz); ^{13}C NMR (125 MHz, $CDCl_3$) δ 22.8, 27.6, 28.2, 29.1, 29.3, 29.7, 29.79, 29.84, 29.857, 29.863, 29.9, 30.1, 34.0, 39.2, 114.2, 139.4; HRMS (ESI^+) calcd for $C_{17}H_{34}Ag$ $[M + Ag]^+$ *m*/*z* 345.1706, found 345.1716.

14-Methylpentadecanoic acid (5; iso-C_{16} acid)

Compound **19** (2.70 g, 11.3 mmol) was subjected to the same conditions as compound **11** (in the preparation of **1**), to afford **5** as a lustrous white solid with a strong odor (2.71 g, 93%); mp 61−63 °C (lit.[3] mp 62.4 °C); ^{1}H NMR (500 MHz, $CDCl_3$) δ 0.87 (6H, d, *J* = 6.6 Hz), 1.12-1.19 (2H, m), 1.24-1.34 (18 H), 1.52 (1H, t of sept, *J* = 6.6, 6.6 Hz), 1.64 (2H, tt, *J* = 7.4, 7.4 Hz), 2.35 (2H, t, *J* = 7.4 Hz); ^{13}C NMR (125 MHz, $CDCl_3$) δ 22.80, 24.82, 27.6, 28.1, 29.2, 29.4, 29.6, 29.7, 29.79, 29.81, 29.9, 30.1, 34.2, 39.2, 180.1; IR υ 1697, 2851, 2917 cm^{-1}; HRMS (ESI^-) calcd for $C_{16}H_{31}O_2$ $[M – H]^-$ *m/z* 255.2330, found 255.2322.

14-Methylpentadecan-1-ol (20)

$BH_3{\cdot}SMe_2$ (1.21 mL, 12.8 mmol) in dry THF (9 mL) was added dropwise to a stirring solution of **5** (2.71 g, 10.6 mmol) in dry THF (4.5 mL) at 0 °C under nitrogen. A steady gas evolution occurred over 10 min. The reaction mixture was warmed to rt and stirred for 12 h under nitrogen. A mixture of MeOH (2.20 mL) and water (9.00 mL) was then added dropwise to the clear and colorless reaction mixture at 0 °C, again resulting in steady gas evolution, and the mixture was stirred vigorously for 30 min, then warmed to rt and diluted with toluene (200 mL) and evaporated to dryness. The residue was dissolved in a mixture of Et_2O (100 mL) and water (100 mL), and extracted with Et_2O (2 × 100 mL), and the combined organic extracts were washed with brine (150 mL), then dried ($MgSO_4$), filtered and evaporated. Purification of the residue by flash chromatography (Et_2O/hexanes, 10 → 40%) afforded **20** as a lustrous white solid (1.98 g, 78%); mp 30−32 °C (lit. [7] 30 °C); ^{1}H NMR (500 MHz, $CDCl_3$) δ 0.87 (6H, d, *J* = 6.6 Hz), 1.11-1.19 (2H, m), 1.24-1.39 (21H, m), 1.47-1.62 (3H, m), 3.64 (2H, t, *J* = 6.6 Hz); ^{13}C NMR (125 MHz, $CDCl_3$) δ 22.8, 25.9, 27.6, 28.1, 29.6, 29.75, 29.77, 29.81, 29.83, 29.84, 29.9, 30.1, 33.0, 39.2, 63.3. HRMS (ESI^+) calcd for $C_{16}H_{34}NaO$ $[M + Na]^+$ *m/z* 265.2502, found 265.2508.

14-Methylpentadec-1-ene (22)

The primary alcohol **20** (1.98 g, 8.17 mmol) was converted into the corresponding dithiocarbonate ester **21**, then eliminated in the same manner as for **18**, to afford **22** as a colorless and odorless liquid (1.81 g, 98% over two steps); ^{1}H NMR (500 MHz, $CDCl_3$) δ 0.87 (6H, d, *J* = 6.6 Hz), 1.12-1.20 (2H, m), 1.25-1.32 (16H, m), 1.34-1.42 (2H, m), 1.53 (1H, t of sept, *J* = 6.6, 6.6 Hz), 2.02-2.08 (2H, m), 4.92-4.96 (1H, m), 4.97-5.02 (1H, m), 5.82 (1H, ddt, *J* = 17.0, 10.3, 6.7 Hz); ^{13}C NMR (125 MHz, $CDCl_3$) δ 22.8, 27.6, 28.2, 29.1, 29.3, 29.7, 29.8, 29.85, 29.86, 29.90, 30.1, 34.0, 39.2, 114.2, 139.4; HRMS (ESI^+) calcd for $C_{16}H_{32}Ag$ $[M + Ag]^+$ *m*/*z* 331.1550, found 331.1558.

13-Methyltetradecanoic acid (4; iso-C_{15} acid)

The alkene **22** (1.30 g, 5.79 mmol) was subjected to the same AcOH/$KMnO_4$ oxidative cleavage as for **11** (in the preparation of **1**), to afford **4** as a lustrous white solid with a strong odor (1.21 g, 86%); mp 49−50 °C (lit. [8] mp 52 °C); ^{1}H NMR (500 MHz, $CDCl_3$) δ 0.87 (6H, d, *J* = 6.6 Hz), 1.15 (2H, m), 1.20-1.39 (16H, m), 1.51 (1H, t of sept, *J* = 6.6, 6.6 Hz), 1.63 (2H, tt, *J* = 7.4, 7.4 Hz), 2.34 (2H, t, *J* = 7.4 Hz); ^{13}C NMR (125 MHz, $CDCl_3$) δ 22.8, 24.9, 27.6, 28.1, 29.2, 29.4, 29.6, 29.75, 29.79, 29.85, 30.1, 34.2, 39.2, 179.9; IR υ 1698, 2849, 2918 cm^{-1}; HRMS (ESI^-) calcd for $C_{15}H_{29}O_2$ $[M – H]^-$ *m*/*z* 241.2173, found 241.2168.

16-Methylheptadecane-1,16-diol (24)

16-Hexadecanolide **23** (10.0 g, 39.3 mmol) was subjected to the same conditions as described for compound **15**, affording **24** as a lustrous white solid (10.9 g, 97%); mp 73−75 °C; ^{1}H NMR (500 MHz, DMSO-d_6) δ 1.04 (6H, s), 1.20-1.35 (26H, m), 1.35-1.40 (2H, m), 3.34-3.38 (2H, m), 4.03 (1H, s), 4.32 (1H, t, *J* = 5.2 Hz); ^{13}C NMR (125 MHz, $CDCl_3$) δ 24.5, 25.9, 29.6, 29.4, 29.6, 29.72, 29.74, 29.75, 29.76-29.8 (4C), 30.3, 33.0, 44.2, 63.2, 71.2; HRMS (ESI^+) calcd for $C_{18}H_{38}NaO_2$ $[M + Na]^+$ *m*/*z* 309.2764, found 309.2768.

16-Methylheptadecan-1-ol (25)

The diol **24** (11.0 g, 38.4 mmol) was subjected to the same conditions as described for compound **16**, affording **25** as a lustrous white solid (9.83 g, 95%); mp 44−46 °C (lit. [9] mp 40.1-40.3 °C); ^{1}H NMR (500 MHz, $CDCl_3$) δ 0.87 (6H, d, *J* = 6.6 Hz), 1.12-1.18 (2H, m), 1.20-1.40 (25H, m), 1.48-1.60 (3H, m), 3.64 (2H, t, *J* = 6.7 Hz); ^{13}C NMR (125 MHz, $CDCl_3$) δ 22.8, 25.9, 27.6, 28.1, 29.6, 29.76, 29.77, 29.81, 29.83, 29.84, 29.85 (2C), 29.88, 30.1, 33.0, 39.2, 63.3; HRMS (ESI^+) calcd for $C_{18}H_{38}NaO$ $[M + Na]^+$ *m/z* 293.28149, found 293.28225.

16-Methylheptadecanoic acid (7; iso-C_{18} acid)

The alcohol **25** (8.50 g, 31.4 mmol) was subjected to the same conditions as described for compound **12** (in the preparation of **2**), affording **7** as a lustrous white solid with a strong odor (7.39 g, 82%); mp 68−69 °C (lit. [3] mp 69.5 °C); ^{1}H NMR (500 MHz, $CDCl_3$) δ 0.87 (6H, d, *J* = 6.6 Hz), 1.12-1.19 (2H, m), 1.24-1.39 (22H, m), 1.52 (1H, t of sept, *J* = 6.6, 6.6 Hz), 1.64 (2H, tt, *J* = 7.5, 7.5 Hz), 2.35 (2H, t, *J* = 7.5 Hz); ^{13}C NMR (125 MHz, $CDCl_3$) δ 22.8, 24.8, 27.6, 28.1, 29.2, 29.4, 29.6, 29.75, 29.79, 29.82, 29.84, 29.85, 29.9, 30.1, 34.2, 39.2, 179.7; IR υ 1698, 2851, 2917 cm^{-1}; HRMS (ESI^-) calcd for $C_{18}H_{35}O_2$ $[M - H]^-$ *m/z* 283.2643, found 283.2636.

16-Methylheptadecyl methanesulfonate (26)

Compound **25** (600 mg, 2.34 mmol) was subjected to the same conditions as compound **12** (in the preparation of **13**). Following work-up and evaporation of the solvent, the residue was purified by flash chromatography (Et_2O/hexanes, 50 → 70%) affording **26** as a colorless waxy solid (750 mg, 96%); ^{1}H NMR (500 MHz, $CDCl_3$) δ 0.87 (6H, d, *J* = 6.6 Hz), 1.12-1.20 (2H, m), 1.22-1.36 (22H, m), 1.36-1.43 (2H, m), 1.52 (1H, t of sept, *J* = 6.6, 6.6 Hz), 1.71-1.79 (2H, m), 3.01 (3H, s), 4.23 (2H, t, *J* = 6.6 Hz); ^{13}C NMR (125 MHz, $CDCl_3$) δ 22.8, 25.6, 27.6, 28.1, 29.2, 29.3, 29.6, 29.7, 29.76, 29.80, 29.83, 29.84, 29.85, 29.9, 30.1, 37.5, 39.2, 70.3. HRMS (ESI^+) calcd for $C_{19}H_{40}NaO_3S$ $[M + Na]^+$ *m/z* 371.2590, found 371.2599.

17-Methyloctadecanenitrile (27)

Compound **26** (659 mg, 1.89 mmol) was subjected to the same conditions as compound **13**. Following work-up and evaporation of the solvent the residue was purified by flash chromatography (Et_2O/hexanes, 50 → 70%) affording **27** as a colorless waxy solid (394 mg, 75%); ^1H NMR (500 MHz, $CDCl_3$) δ 0.86 (6H, d, *J* = 6.6 Hz), 1.11-1.18 (2H, m), 1.22-1.36 (22H, m), 1.40-1.47 (2H, m), 1.52 (1H, t of sept, *J* = 6.6, 6.6 Hz), 1.60-1.68 (2H, m), 2.32 (2H, t, *J* = 7.1 Hz); ^{13}C NMR (125 MHz, $CDCl_3$) δ 17.2, 22.7, 25.5, 27.5, 28.1, 28.8, 28.9, 29.4, 29.6, 29.68, 29.73, 29.76, 29.78, 29.79, 29.82, 30.0, 39.2, 119.9. HRMS (ESI^+) calcd for $C_{19}H_{38}N$ $[M + H]^+$ *m/z* 280.2999, found 280.3014.

17-Methyloctadecanoic acid (8; iso-C_{19} acid)

Compound **27** (1.10 g, 3.94 mmol) was subjected to the same conditions as compound **14**, to afford **8** as a lustrous white solid with a strong odor (1.01 g, 86%); mp 66−68 °C (lit. [10] mp 67.3-67.8 °C); ^1H NMR (500 MHz, $CDCl_3$) δ 0.86 (6H, d, *J* = 6.6 Hz), 1.10-1.19 (2H, m), 1.20-1.36 (24H, m), 1.52 (1H, t of sept, *J* = 6.6, 6.6 Hz), 1.64 (2H, tt, *J* = 7.5, 7.5 Hz), 2.35 (2H, t, *J* = 7.5 Hz); ^{13}C NMR (125 MHz, $CDCl_3$) δ 22.8, 24.8, 27.6, 28.1, 29.2, 29.4, 29.6, 29.7, 29.79, 29.82, 29.83, 29.85 (2C), 29.9, 30.1, 34.0, 39.2, 179.0; IR υ 1699, 2849, 2917 cm^{-1}; HRMS (ESI^-) calcd for $C_{19}H_{37}O_2$ $[M - H]^-$ *m/z* 297.2799, found 297.2795.

(S)*-4-Benzyl-*N-(15-methylhexadecanoyl)oxazolidin-2-one (28)

Et_3N (1.80 mL, 12.9 mmol), then pivalyl chloride (501 μL, 4.07 mmol) were added to a solution of **6** (1.00 g, 3.70 mmol) in dry THF (37 mL) at −30 °C and the mixture was stirred under nitrogen for 1 h. Dried LiCl (784 mg, 18.5 mmol), DMAP (45.2 mg, 370 μmol) and (*S*)-4-benzyloxazolidin-2-one (721 mg, 4.07 mmol) were added in one portion at −30 °C, then the reaction mixture was warmed to rt and stirred under nitrogen for 16 h. The reaction mixture was filtered and the filtrate

was evaporated to dryness in vacuo, then the orange solid residue was dissolved in a mixture of water (100 mL) and Et_2O (100 mL). The organic phase was collected and the remaining aqueous phase was extracted with Et_2O (2 × 50 mL). The combined organic phases were dried ($MgSO_4$), filtered and evaporated in vacuo. Purification of the residue by flash chromatography (Et_2O/hexane, 5 → 50%) afforded **28** as a white solid (1.13 g, 71%); mp 46−48 °C; $[\alpha]^{22}_{D}$ +48.9 (*c* 1.0, CH_2Cl_2); ^{1}H NMR (500 MHz, $CDCl_3$) δ 0.87 (6H, d, *J* = 6.6 Hz), 1.12-1.19 (2H, m), 1.24-1.41 (20H, m), 1.52 (1H, t of sept, *J* = 6.6, 6.6 Hz), 1.65-1.74 (2H, m), 2.77 (1H, dd, *J* = 13.4, 9.6 Hz), 2.85-3.01 (2H, m), 3.30 (1H, dd, *J* = 13.3, 3.4 Hz), 4.15-4.21 (2H, m), 4.64-4.70 (1H, m), 7.19-7.24 (2H, m), 7.25-7.30 (1H, m), 7.31-7.37 (2H, m); ^{13}C NMR (125 MHz, $CDCl_3$) δ 22.8, 24.4, 27.6, 28.1, 29.3, 29.5, 29.6, 29.75, 29.80, 29.817, 29.824, 29.9, 30.1, 35.7, 38.1, 39.2, 55.3, 66.3, 127.4, 129.1, 129.6, 135.5, 153.6, 173.6; HRMS (ESI^+) calcd for $C_{27}H_{43}NNaO_3$ $[M + Na]^+$ *m*/*z* 452.3135, found 452.3146.

(S)-2,15-Dimethylhexadecanoic acid (30)

(a) (*S*)-4-Benzyl-*N*-((*S*)-2,15-dimethylhexadecanoyl)oxazolidin-2-one (**29**). NaHMDS (1 M in THF, 2.81 mL, 2.81 mmol) was added dropwise to a solution of **28** (240 mg, 561 μmol) in dry THF (2.0 mL) at −78 °C and the resulting mixture was stirred under nitrogen for 90 min. MeI (175 μL, 2.81 mmol) was then added in one portion at −78 °C, and the temperature was maintained for a further 2 h with stirring under nitrogen. The reaction mixture was acidified by addition of sat. aq NH_4Cl (60 mL) and warmed to rt, then extracted with Et_2O (3 × 25 mL). The combined bright yellow organic phases were washed with brine (2 × 25 mL), then dried ($MgSO_4$), filtered and evaporated in vacuo. Purification of the residue by flash chromatography (Et_2O/hexanes, 0 → 25%) afforded **29** as white solid (119 mg, 80%); $[\alpha]^{25}_{D}$ +54.8 (*c* 1.0, CH_2Cl_2); ^{1}H NMR (500 MHz, $CDCl_3$) δ 0.87 (6H, d, *J* = 6.6 Hz), 1.12-1.18 (2H, m), 1.22 (3H, d, *J* = 6.9 Hz), 1.24-1.35 (20H, m), 1.37-1.46 (1H, m), 1.52 (1H, t of sept, *J* = 6.6, 6.6 Hz), 1.69-1.79 (1H, m), 2.77 (1H, dd, *J* = 9.7, 13.4 Hz), 3.27 (1H, dd, *J* = 3.2, 13.4 Hz), 3.71 (1H, tq, *J* = 6.8, 6.8 Hz), 4.15-4.21 (2H, m), 4.65-

4.71 (1H, m), 7.20-7.24 (2H, m), 7.25-7.29 (1H, m), 7.30-7.35 (2H, m); ^{13}C NMR (125 MHz, $CDCl_3$) δ 17.5, 22.8, 27.4, 27.5, 28.1, 29.6, 29.7, 29.77, 29.79 (2C), 29.81, 29.85, 30.1, 33.6, 37.8, 38.1, 39.2, 55.5, 66.1, 127.4, 129.0, 129.6, 135.5, 153.2, 177.5.

(b) (*S*)-2,15-Dimethylhexadecanoic acid (**30**). LiOH·H_2O (79.9 mg, 1.90 mmol) and 30% aq H_2O_2 (389 µL, 3.81 mmol) were added to a solution of **29** (169 mg, 381 µmol) in a mixture of THF (3.8 mL) and water (1.9 mL) at 0 °C, then the reaction mixture was warmed to rt and stirred under nitrogen for 12 h. The reaction mixture was diluted into water (10 mL) and then acidified to pH 2 by slow addition of 5 M aq HCl at 0 °C. The cloudy white suspension was extracted with Et_2O (3 × 15 mL) and the combined organic phases were dried ($MgSO_4$), filtered and evaporated in vacuo to give a clear, pale yellow liquid. The liquid was purified by flash chromatography (hexanes/EtOAc/AcOH, 78:20:2), affording **30** as a waxy yellow solid (106 mg, 98%); mp 45−46 °C; $[\alpha]^{25}_D$ +10.4 (*c* 2.0, $CHCl_3$); ^1H NMR (500 MHz, $CDCl_3$) δ 0.87 (6H, d, *J* = 6.6 Hz), 1.13-1.19 (5H, m), 1.22-1.36 (20H, m), 1.39-1.47 (1H, m), 1.52 (1H, t of sept, *J* = 6.6, 6.6 Hz), 1.64-1.74 (1H, m), 2.46 (1H, tq, *J* = 6.9, 6.9 Hz); ^{13}C NMR (125 MHz, $CDCl_3$) δ 17.0, 22.8, 27.3, 27.6, 28.1, 29.6, 29.7, 29.76, 29.80, 29.83, 29.85, 29.9, 30.1, 33.7, 39.2, 39.5, 183.5; IR υ 1692, 2850, 2915 cm^{-1}; HRMS (ESI^-) calcd for $C_{18}H_{35}O_2$ $[M – H]^-$ *m*/*z* 283.2643, found 283.2637.

3-Phenyl-2-(phenylsulfonyl)-1,2-oxaziridine (Davis oxaziridine)

A solution of K_2CO_3 (4.75 g, 34.4 mmol) in water (25 mL), then a solution of Oxone® (3.13 g, 5.10 mmol) in water (25 mL), were added sequentially to a vigorously stirring solution of *N*-benzylidenebenzenesulfonamide (1.00 g, 4.08 mmol) in toluene (20 mL) at rt. Reaction progress was followed by thin layer chromatography of the organic phase (Et_2O/hexanes 40:60). The reaction mixture was then diluted into water (100 mL), the organic phase was collected and the remaining aqueous phase was extracted with toluene (2 × 100 mL). The combined organic phases were washed with 10% aq Na_2SO_3 (2 × 20 mL), then dried ($MgSO_4$), filtered and evaporated in vacuo. The residue was purified by flash chromatography (Et_2O/hexanes, 10 → 20%), affording the

product as a lustrous white solid (1.06 g, 99%); ^{1}H NMR (500 MHz, $CDCl_3$) δ 5.50 (1H, s), 7.40-7.50 (5H, m), 7.62-7.67 (2H, m), 7.74-7.79 (1H, m), 8.04-8.08 (2H, m); ^{13}C NMR (125 MHz, $CDCl_3$) δ 78.45, 128.40, 128.91, 129.53, 129.54, 130.62, 131.60, 134.91, 135.16.

(S)-2-Hydroxy-15-methylhexadecanoic acid (32)

(a) (*S*)-4-Benzyl-*N*-((*S*)-2-hydroxy-15-methylhexadecanoyl)oxazolidin-2-one (**31**). NaHMDS (1 M in THF, 935 μL, 935 μmol) was added dropwise to a solution of **28** (100 mg, 234 μmol) in dry THF (800 μL) at −78 °C and the resulting mixture was stirred under nitrogen for 90 min. A solution of 3-phenyl-2-(phenylsulfonyl)-1,2-oxaziridine [11] (153 mg, 585 μmol) in dry THF (3.51 mL) was pre-cooled to −78 °C, then rapidly transferred via cannula into the reaction mixture, followed immediately by the addition of a pre-cooled solution of anhydrous 10-camphorsulfonic acid (2.72 mg, 1.17 mmol) in dry THF (1.99 mL) via cannula. The reaction mixture was warmed to rt and diluted with water (50 mL), then extracted with Et_2O (3 × 30 mL). The combined organic phases were dried ($MgSO_4$), filtered and evaporated in vacuo, then the residue was purified by flash chromatography (EtOAc/hexanes, 5 → 15%), affording **31** as a white solid (73.8 mg, 71%); $[\alpha]^{25}_D$ +30.4 (*c* 0.5, $CHCl_3$); ^{1}H NMR (500 MHz, $CDCl_3$) δ 0.87 (6H, d, *J* = 6.6 Hz), 1.11-1.19 (2H, m), 1.23-1.40 (18H, m), 1.47-1.63 (4H, m), 1.76-1.86 (1H, m), 2.85 (1H, dd, *J* = 13.5, 9.4 Hz), 3.32 (1H, dd, *J* = 13.6, 3.3 Hz), 3.44 (1H, d, *J* = 8.0 Hz), 4.21-4.33 (2H, m), 4.63-4.71 (1H, m), 5.00 (1H, td, *J* = 8.0, 3.5 Hz), 7.19-7.24 (2H, m), 7.26-7.32 (1H, m), 7.32-7.38 (2H, m); ^{13}C NMR (125 MHz, $CDCl_3$) δ 22.8, 25.4, 27.6, 28.1, 29.4, 29.66, 29.72, 29.79, 29.82, 29.83, 29.87, 30.1, 34.4, 37.7, 39.2, 55.7, 67.0, 71.0, 127.7, 129.2, 129.6, 135.0, 153.3, 175.2.

(b) Methyl (*S*)-2-Hydroxy-15-methylhexadecanoate. Isopropylmagnesium chloride (2.0 M in THF, 224 μL, 449 μmol) was added dropwise to dry MeOH (718 μL) at 0 °C under nitrogen. A solution of **31** (100 mg, 224 μmol) in dry MeOH (1.44 mL) was then added in one portion, and the reaction mixture was stirred vigorously at 0 °C under nitrogen for 15 min. The reaction was quenched by addition of sat. aq NH_4Cl (5 mL), causing precipitation of a white material. The solvent was

evaporated in vacuo, and the residue was dissolved into a mixture of water (15 mL) and Et_2O (15 mL). The organic phase was collected and the remaining aqueous phase was extracted with Et_2O (2 × 10 mL). The combined organic phases were washed with brine (15 mL), then dried ($MgSO_4$), filtered and evaporated in vacuo. The residue was purified by flash chromatography (EtOAc/hexanes, 10 → 20%) to afford methyl (*S*)-2-Hydroxy-15-methylhexadecanoate as a colorless liquid (51.0 mg, 76%); $[\alpha]^{27}_D$ +5.1 (*c* 2.5, $CHCl_3$); ^{1}H NMR (500 MHz, $CDCl_3$) δ 0.87 (6H, d, *J* = 6.6 Hz), 1.11-1.18 (2H, m), 1.22-1.46 (20H, m), 1.51 (1H, t of sept, *J* = 6.6, 6.6 Hz), 1.59-1.68 (1H, m), 1.74-1.83 (1H, m), 2.71 (1H, d, *J* = 5.8 Hz), 3.79 (3H, s), 4.16-4.22 (1H, m); ^{13}C NMR (125 MHz, $CDCl_3$) δ 22.8, 24.9, 27.6, 28.1, 29.4, 29.6, 29.7, 29.77, 29.81, 29.83, 29.9, 30.1, 34.6, 39.2, 52.6, 70.6, 176.0.

(c) (*S*)-2-Hydroxy-15-methylhexadecanoic acid (**32**). A solution of NaOH in MeOH (3 M, 200 μL) was added to a solution of methyl (*S*)-2-hydroxy-15-methylhexadecanoate (49.0 mg, 163 μmol) in CH_2Cl_2 (2.9 mL) and MeOH (320 μL) and the resulting mixture was stirred under nitrogen at rt for 12 h. The cloudy white reaction mixture was evaporated to dryness and the residue was suspended in water (15 mL), then acidified by addition of conc HCl until pH < 1 was observed by low pH indicator paper. The mixture was extracted with CH_2Cl_2 (3 × 15 mL), and the combined organic phases were dried ($MgSO_4$), filtered and evaporated in vacuo. The residue was purified by flash chromatography (hexanes/EtOAc/TFA, 49:49:2), affording **32** as a white powder (39.0 mg, 83%); mp 65 °C (dec.); $[\alpha]^{25}_D$ +2.79 (*c* 1.6, 2% TFA in CH_2Cl_2); ^{1}H NMR (500 MHz, $CDCl_3/CD_3OD$, 1:1) δ 0.83 (6H, d, *J* = 6.6 Hz), 1.07-1.16 (2H, m), 1.19-1.34 (18H, m), 1.36-1.44 (2H, br m), 1.48 (1H, t of sept, *J* = 6.6, 6.6 Hz), 1.56-1.69 (1H, br m), 1.69-1.84 (1H, br m), 4.05-4.16 (1H, br m); ^{13}C NMR (125 MHz, 2% TFA in DMSO-d_6) δ 22.5, 24.8, 26.8, 27.4, 28.9, 29.03, 29.04, 29.09, 29.10, 29.13, 29.4, 34.0, 38.5, 42.9, 69.7, 176.0; IR υ 1138, 1195, 1676, 3419 cm^{-1}; HRMS (ESI$^-$) calcd for $C_{17}H_{33}O_3$ $[M - H]^-$ *m/z* 285.2429, found 285.2425.

14-Methylpentadecan-2-one (33)

A mixture of $PdCl_2$ (11.8 mg, 66.8 μmol), dimethylacetamide (4.5 mL) and water (270 μL) was stirred at 90 °C under oxygen for 4 h. A solution of **22** (100 mg, 456 μmol) in dimethylacetamide (200 μL) was then added, and the reaction mixture quickly became dark in color. The reaction mixture was stirred at 90 °C under oxygen for a further 5 d, and the resulting orange reaction mixture was then evaporated in vacuo. The residue was diluted with water (25 mL) and extracted with Et_2O (3 × 25 mL). The combined organic phases were washed with brine (25 mL), then dried ($MgSO_4$), filtered and evaporated. Purification of the residue by flash chromatography (Et_2O/hexanes, 5 → 15%) afforded **33** as a colorless liquid (55.0 mg, 51%); ^{1}H NMR (500 MHz, $CDCl_3$) δ 0.86 (6H, d, J = 6.6 Hz), 1.10-1.18 (2H, m), 1.21-1.31 (16H, m), 1.47-1.59 (3H, m), 2.13 (3H, s), 2.41 (2H, t, J = 7.5 Hz); ^{13}C NMR (125 MHz, $CDCl_3$) δ 22.8, 24.0, 27.6, 28.1, 29.3, 29.5, 29.6, 29.75, 29.79, 29.84, 30.0, 30.1, 39.2, 44.0, 209.5; IR υ 1718, 2853, 2923 cm^{-1}; HRMS (ESI^+) calcd for $C_{16}H_{32}NaO$ $[M + Na]^+$ m/z 263.2345, found 263.2349.

References

(1) Still, W. C.; Kahn, M.; Mitra, A. M. *J Org Chem* **1978**, *43*, 2923.

(2) Pangborn, A. B.; Giardello, M. A.; Grubbs, R. H.; Rosen, R. K.; Timmers, F. J. *Organometallics* **1996**, *15*, 1518.

(3) Weitkamp, A. W. *J. Am. Chem. Soc.* **1945**, *67*, 447.

(4) Shorland, F. B.; Gerson, T.; Hansen, R. P. *Biochem. J.* **1955**, *61*, 702.

(5) Yasuda, M.; Yamasaki, S.; Onishi, Y.; Baba, A. *J. Am. Chem. Soc.* **2004**, *126*, 7186.

(6) Garcia, J.; López, M.; Romeu, J. *Tetrahedron: Asymmetry* **1999**, *10*, 2617.

(7) Milburn, A. H.; Truter, E. V. *J. Chem. Soc.* **1954**, 3344.

(8) Hansen, R. P.; Shorland, F. B.; Cooke, N. J. *Biochem. J.* **1954**, *57*, 297.

(9) Stallberg-Stenhagen, S.; Stenhagen, E. *Arkiv foer Kemi, Mineralogi och Geologi* **1944**, *19A*, 1.

(10) Cason, J. *J. Am. Chem. Soc.* **1942**, *64*, 1106.

(11) Davis, F. A.; Chattopadhyay, S.; Towson, J. C.; Lal, S.; Reddy, T. *J. Org. Chem.* **1988**, *53*, 2087.
