# Supplementary material for: A practical synthesis of long-chain iso-fatty acids (iso-C12–C19) and related natural products
Source: Beilstein J Org Chem. 2013 Sep 4;9:1807–12. doi: 10.3762/bjoc.9.210 (PMC3778388; doi:10.3762/bjoc.9.210)

Supporting Information

for

A practical synthesis of long-chain iso-fatty acids (iso-C_{12}–C_{19}) and related natural products

Mark B. Richardson and Spencer J. Williams*

Address: School of Chemistry, Bio21 Molecular Science and Biotechnology Institute, University of Melbourne, Parkville, Victoria 3010, Australia

Email: Spencer J. Williams - sjwill@unimelb.edu.au

* Corresponding author

NMR spectra

Table of contents

^{1}H NMR spectrum of 10-methylundecanoic acid (**1**) ………….. S4
^{13}C NMR spectrum of 10-methylundecanoic acid (**1**) ……….… S5
^{1}H NMR spectrum of 11-methyldodecanoic acid (**2**) ……….…..S6
^{13}C NMR spectrum of 11-methyldodecanoic acid (**2**) ...….…….. S7
^{1}H NMR spectrum of 12-methyltridecanoic acid (**3**) ………….. S8
^{13}C NMR spectrum of 12-methyltridecanoic acid (**3**) ………….. S9
^{1}H NMR spectrum of 13-methyltetradecanoic acid (**4**) ..……….. S10
^{13}C NMR spectrum of 13-methyltetradecanoic acid (**4**) ..……….. S11
^{1}H NMR spectrum of 14-methylpentadecanoic acid (**5**) ………… S12
^{13}C NMR spectrum of 14-methylpentadecanoic acid (**5**) ………… S13
^{1}H NMR spectrum of 15-methylhexadecanoic acid (**6**) ………… S14

^{13}C NMR spectrum of 15-methylhexadecanoic acid (**6**) S15
^{1}H NMR spectrum of 16-methylheptadecanoic acid (**7**) S16
^{13}C NMR spectrum of 16-methylheptadecanoic acid (**7**) S17
^{1}H NMR spectrum of 17-methyloctatadecanoic acid (**8**) S18
^{13}C NMR spectrum of 17-methyloctatadecanoic acid (**8**) S19
^{1}H NMR spectrum of 2-methyldodec-11-en-2-ol (**10**) S20
^{13}C NMR spectrum of 2-methyldodec-11-en-2-ol (**10**) S21
^{1}H NMR spectrum of 11-methyldodec-1-ene (**11**) S22
^{13}C NMR spectrum of 11-methyldodec-1-ene (**11**) S23
^{1}H NMR spectrum 11-methyldodecan-1-ol (**12**) S24
^{13}C NMR spectrum of 11-methyldodecan-1-ol (**12**) S25
^{1}H NMR spectrum of 11-methyldodecyl methanesulfonate (**13**) S26
^{13}C NMR spectrum of 11-methyldodecyl methanesulfonate (**13**) S27
^{1}H NMR spectrum of 12-methyltridecanenitrile (**14**) S28
^{13}C NMR spectrum of 12-methyltridecanenitrile (**14**) S29
^{1}H NMR spectrum of 15-methylhexadecane-1,15-diol (**16**) S30
^{13}C NMR spectrum of 15-methylhexadecane-1,15-diol (**16**) S31
^{1}H NMR spectrum of 15-methylhexadecan-1-ol (**17**) S32
^{13}C NMR spectrum of 15-methylhexadecan-1-ol (**17**) S33
^{1}H NMR spectrum of *S*-methyl-*O*-15-methylhexadecyl dithiocarbonate (**18**) S34
^{13}C NMR spectrum of *S*-methyl-*O*-15-methylhexadecyl dithiocarbonate (**18**) S35
^{1}H NMR spectrum of 15-methylhexadecan-1-ene (**19**) S36
^{13}C NMR spectrum of 15-methylhexadecan-1-ene (**19**) S37
^{1}H NMR spectrum of 14-methylpentadecan-1-ol (**20**) S38
^{13}C NMR spectrum of 14-methylpentadecan-1-ol (**20**) S39
^{1}H NMR spectrum of 14-methylpentadec-1-ene (**22**) S40
^{13}C NMR spectrum of 14-methylpentadec-1-ene (**22**) S41
^{1}H NMR spectrum of 16-methylheptadecane-1,16-diol (**24**) S42
^{13}C NMR spectrum of 16-methylheptadecane-1,16-diol (**24**) S43
^{1}H NMR spectrum of 16-methylheptadecan-1-ol (**25**) S44
^{13}C NMR spectrum of 16-methylheptadecan-1-ol (**25**) S45
^{1}H NMR spectrum of 16-methylheptadecyl methanesulfonate (**26**) S46
^{13}C NMR spectrum of 16-methylheptadecyl methanesulfonate (**26**) S47

^{1}H NMR spectrum of 17-methyloctadecanenitrile (**27**) ………… S48
^{13}C NMR spectrum of 17-methyloctadecanenitrile (**27**) ………… S49
^{1}H NMR spectrum of (*S*)-4-benzyl-3-(15-methylhexadecanoyl)oxazolidin-2-one (**28**) ………… S50
^{13}C NMR spectrum of (*S*)-4-benzyl-3-(15-methylhexadecanoyl)oxazolidin-2-one (**28**) ………… S51
^{1}H NMR spectrum of (*S*)-4-benzyl-3-((*S*)-2,15-dimethylhexadecanoyl)oxazolidin-2-one (**29**) ………… S52
^{13}C NMR spectrum of (*S*)-4-benzyl-3-((*S*)-2,15-dimethylhexadecanoyl)oxazolidin-2-one (**29**) ………… S53
^{1}H NMR spectrum of (*S*)-2,15-dimethylhexadecanoic acid (**30**) ………… S54
^{13}C NMR spectrum of (*S*)-2,15-dimethylhexadecanoic acid (**30**) ………… S55
^{1}H NMR spectrum of 3-Phenyl-2-(phenylsulfonyl)-1,2-oxaziridine ………… S56
^{13}C NMR spectrum of 3-Phenyl-2-(phenylsulfonyl)-1,2-oxaziridine ………… S57
^{1}H NMR spectrum of (*S*)-4-benzyl-3-((*S*)-2-hydroxy-15-methylhexadecanoyl)oxazolidin-2-one (**31**) ………… S58
^{13}C NMR spectrum of (*S*)-4-benzyl-3-((*S*)-2-hydroxy-15-methylhexadecanoyl)oxazolidin-2-one (**31**) ………… S59
^{1}H NMR spectrum of (*S*)-methyl 2-hydroxy-15-methylhexadecanoate ………… S60
^{13}C NMR spectrum of (*S*)-methyl 2-hydroxy-15-methylhexadecanoate ………… S61
^{1}H NMR spectrum of (*S*)-2-hydroxy-15-methylhexadecanoic acid (**32**) ………… S62
^{13}C NMR spectrum of (*S*)-2-hydroxy-15-methylhexadecanoic acid (**32**) ………… S63
^{1}H NMR spectrum of 14-methylpentadecan-2-one (**33**) ………… S64
^{13}C NMR spectrum of 14-methylpentadecan-2-one (**33**) ………… S65

^{1}H NMR (500 MHz, $CDCl_3$) – 10-Methylundecanoic acid (**1**)

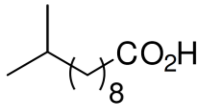

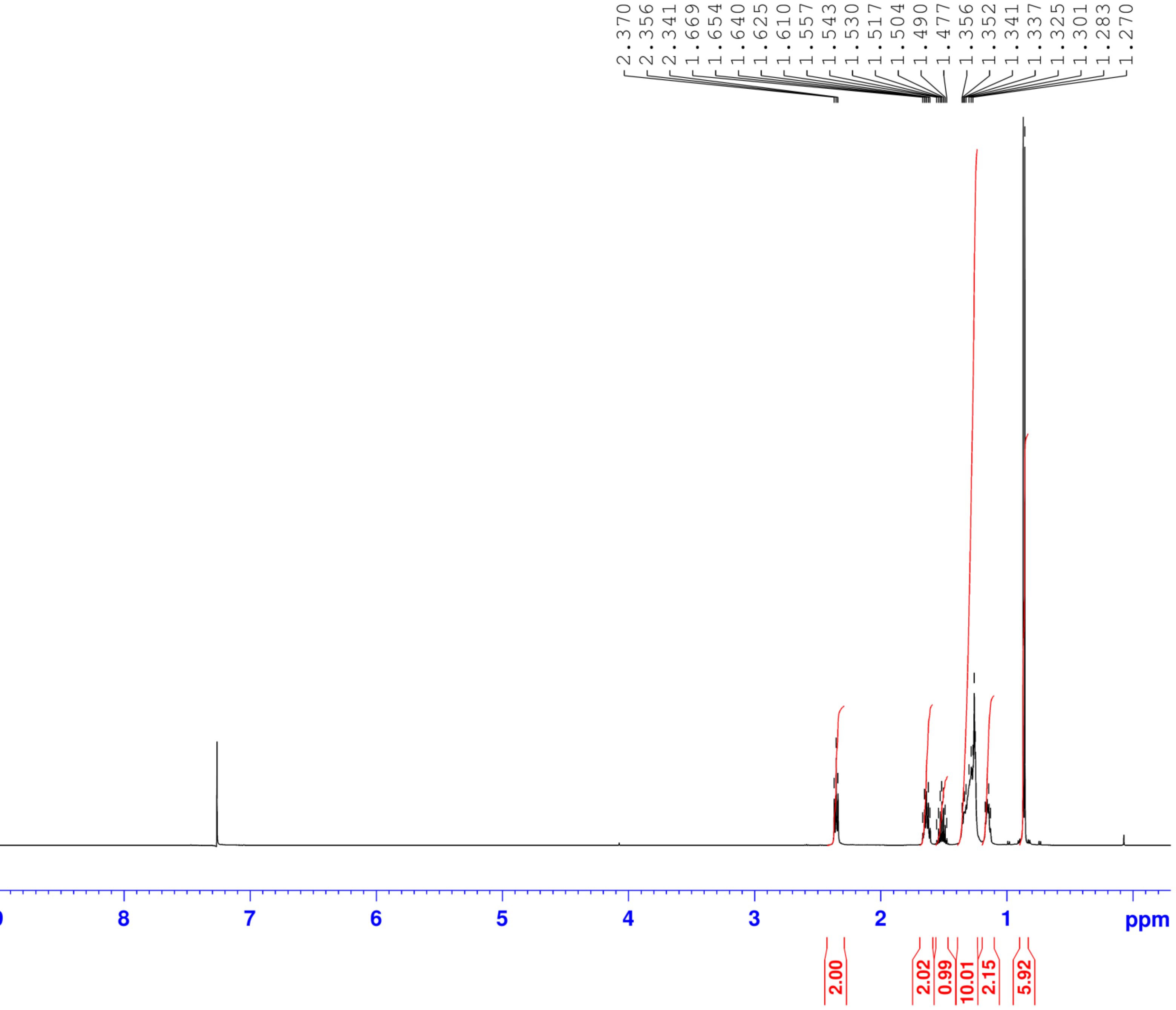

^{13}C NMR (125 MHz, $CDCl_3$) – 10-Methylundecanoic acid (**1**)

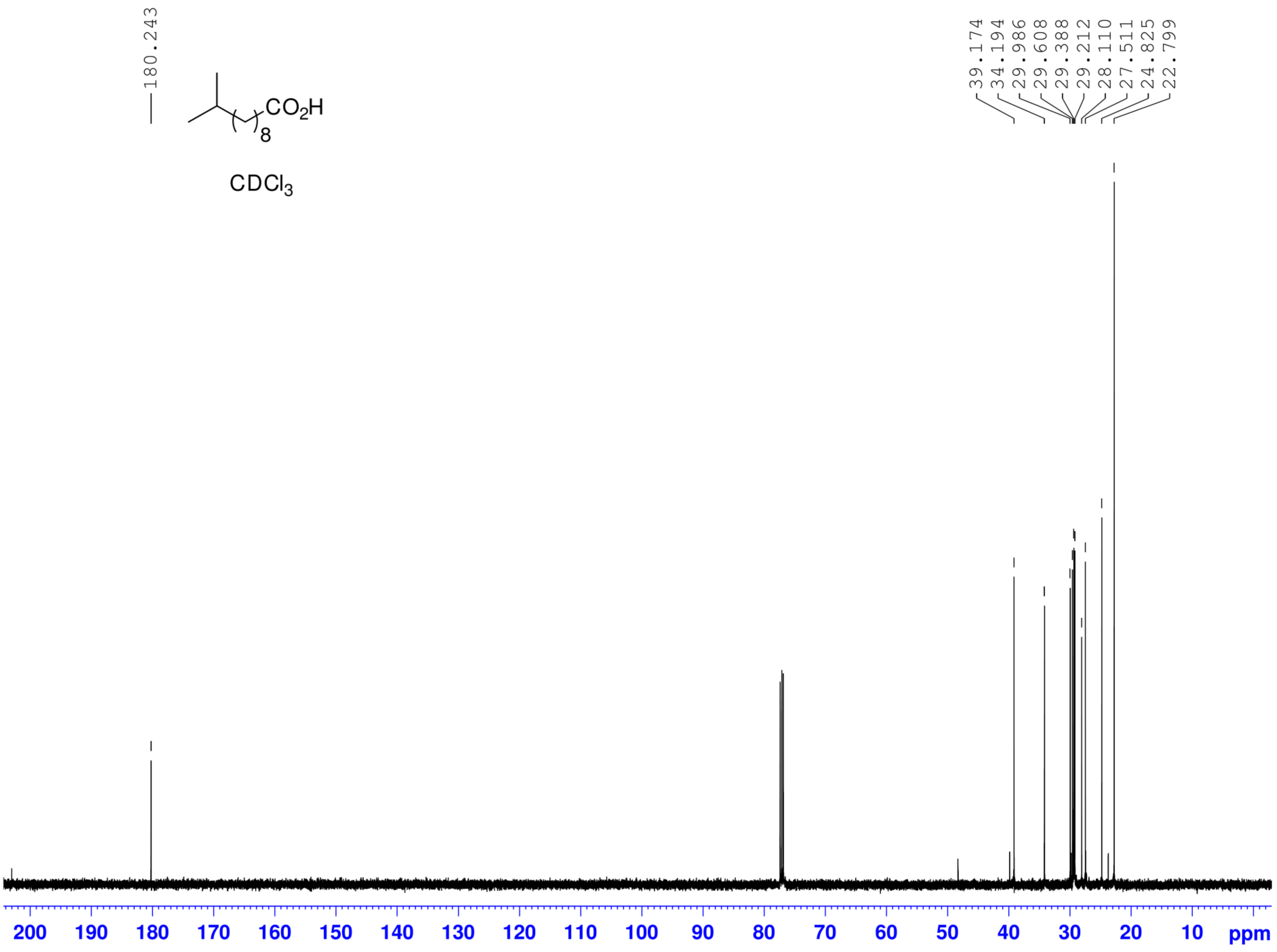

^{1}H NMR (500 MHz, $CDCl_3$) – 11-Methyldodecanoic acid (**2**)

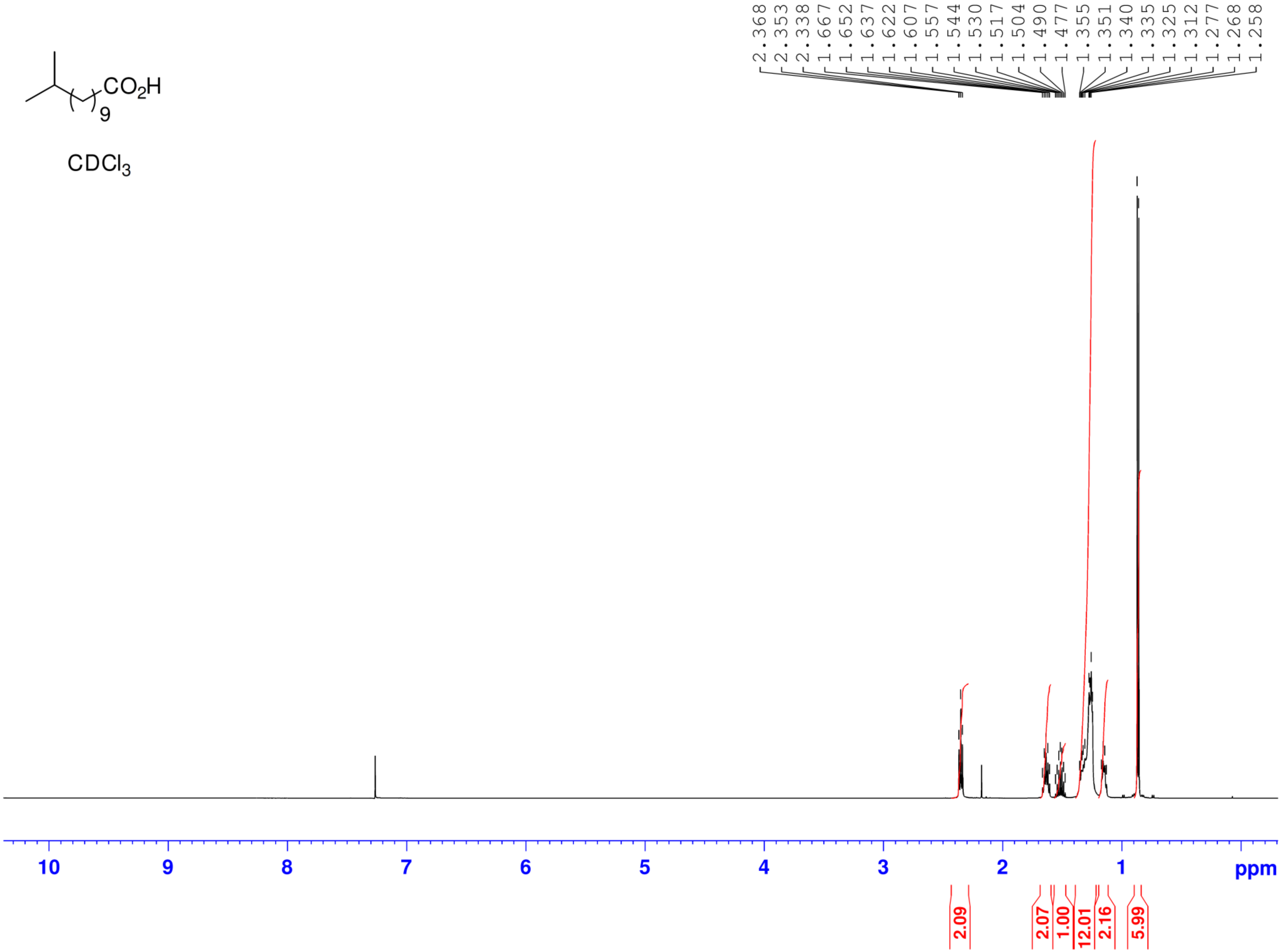

^{13}C NMR (125 MHz, $CDCl_3$) – 11-Methyldodecanoic acid (**2**)

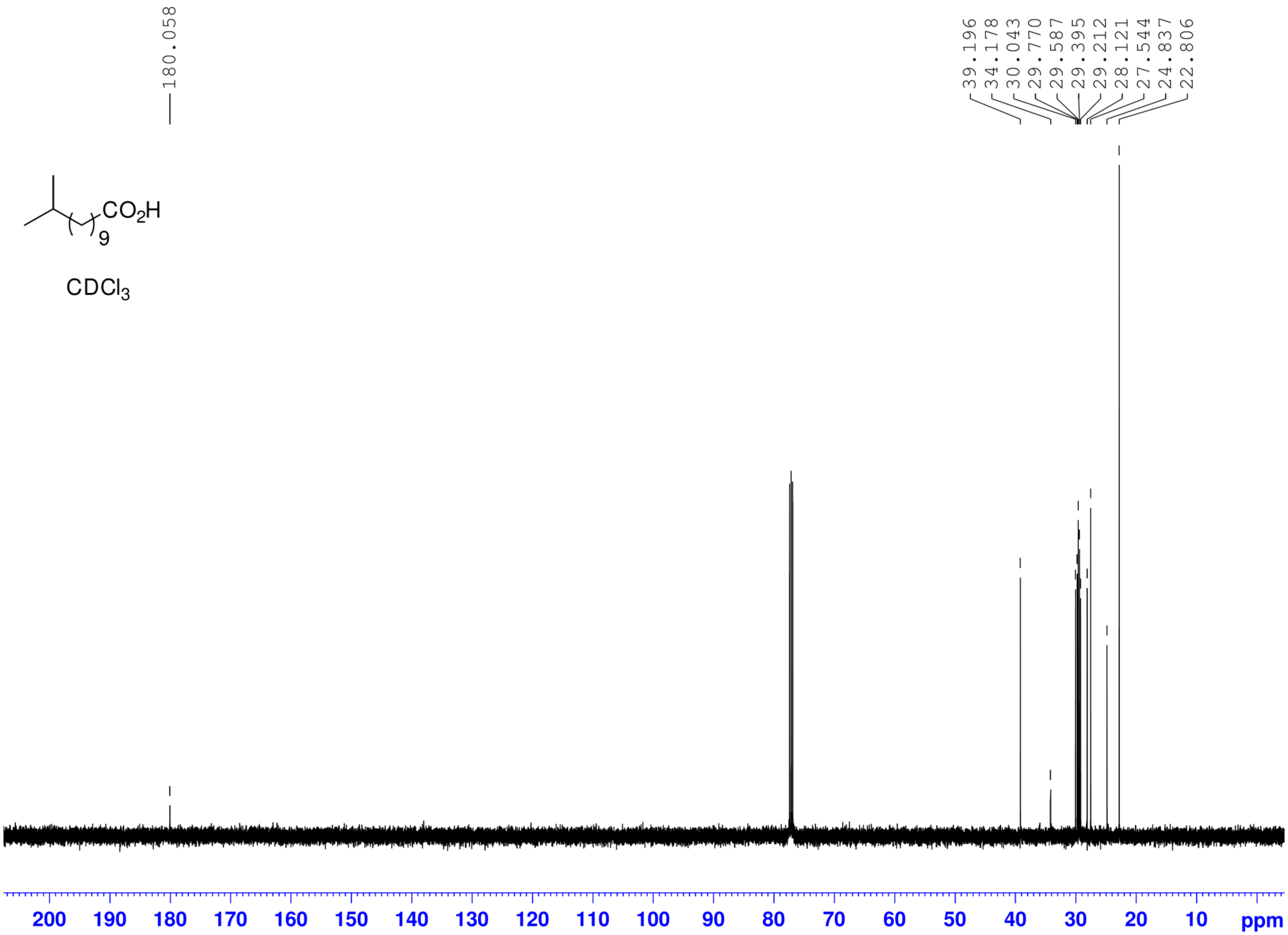

^{1}H NMR (500 MHz, $CDCl_3$) – 12-Methyltridecanoic acid (**3**)

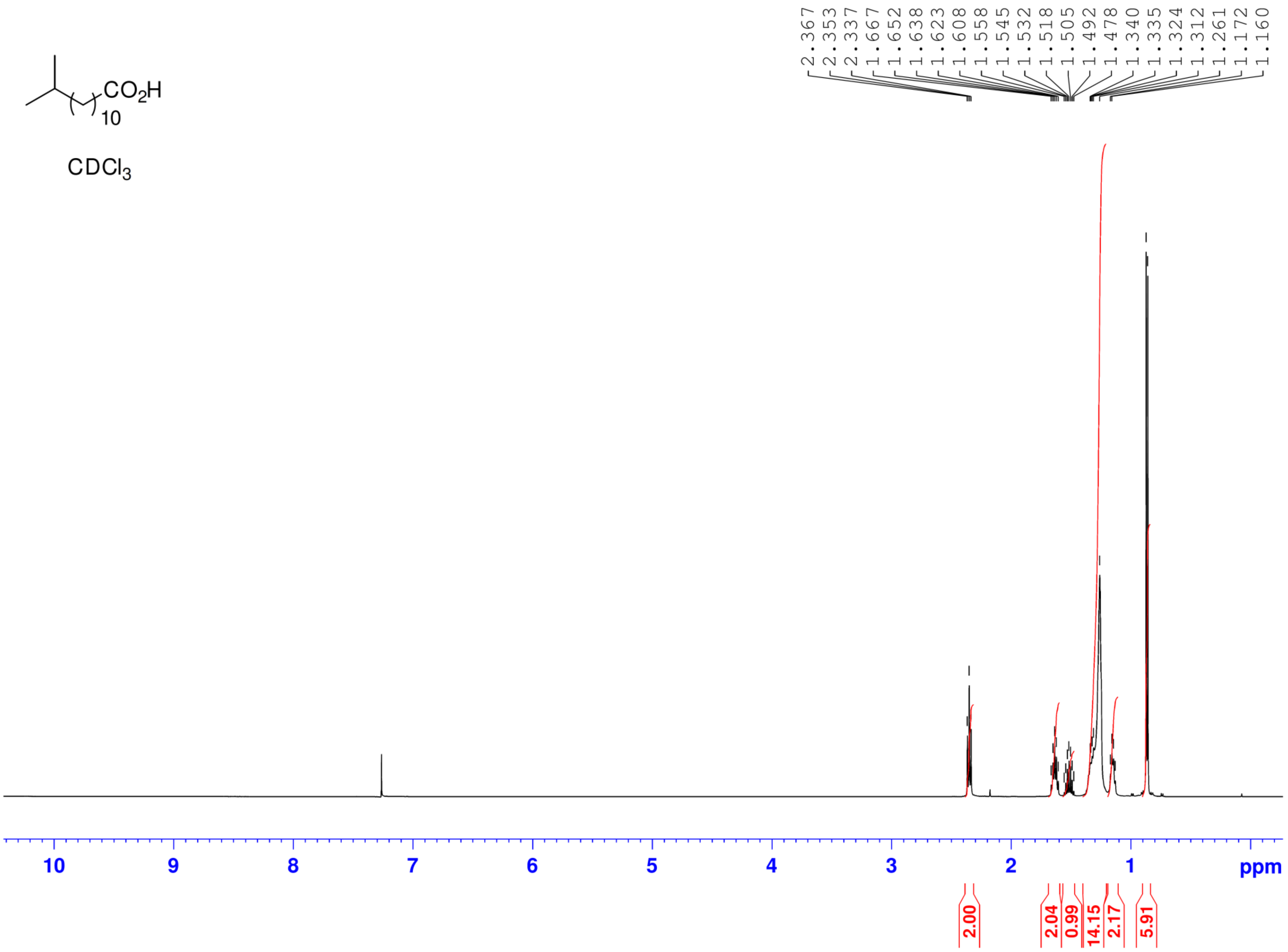

^{13}C NMR (125 MHz, $CDCl_3$) – 12-Methyltridecanoic acid (**3**)

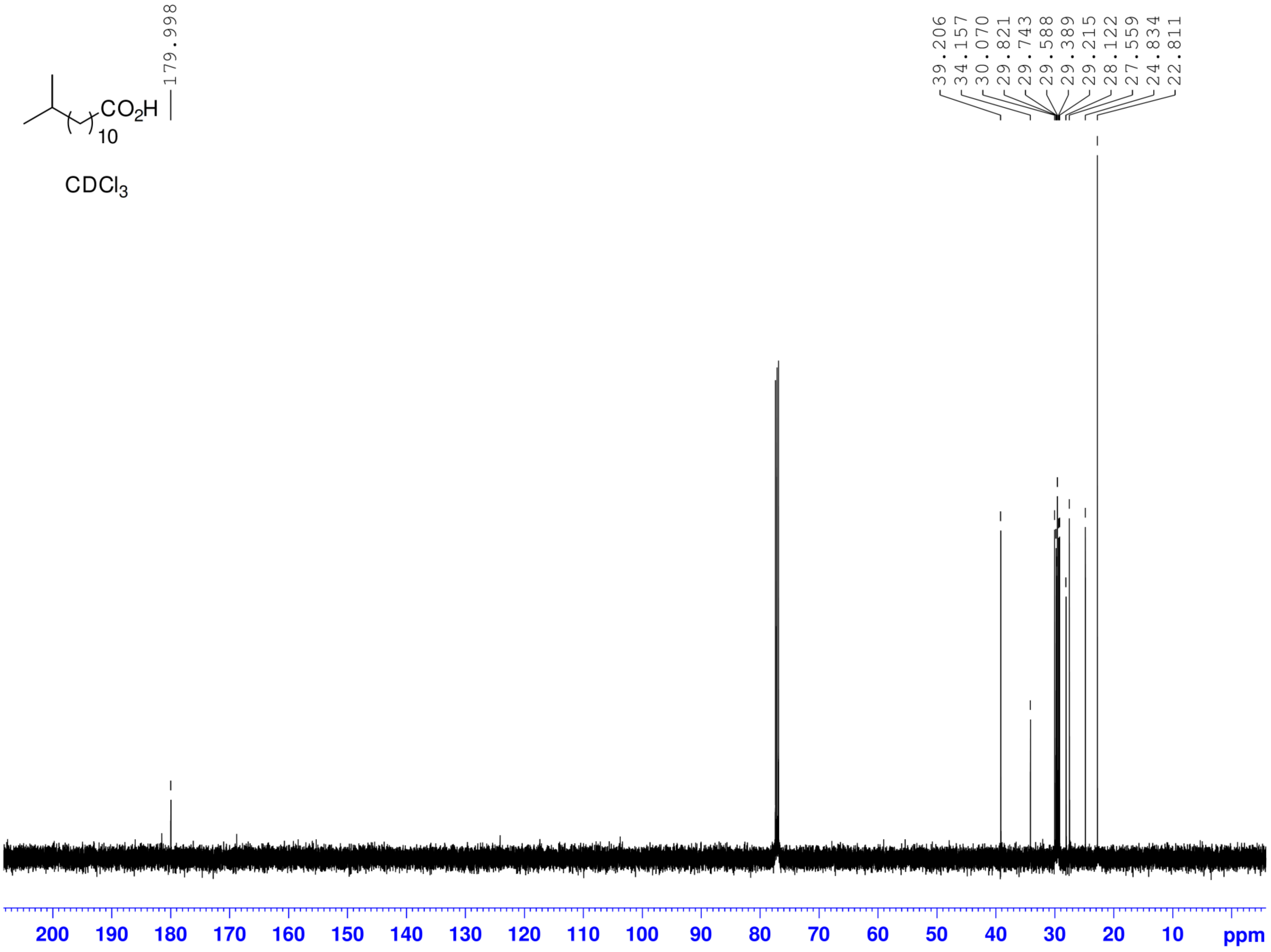

^{1}H NMR (500 MHz, $CDCl_3$) – 13-Methyltetradecanoic acid (**4**)

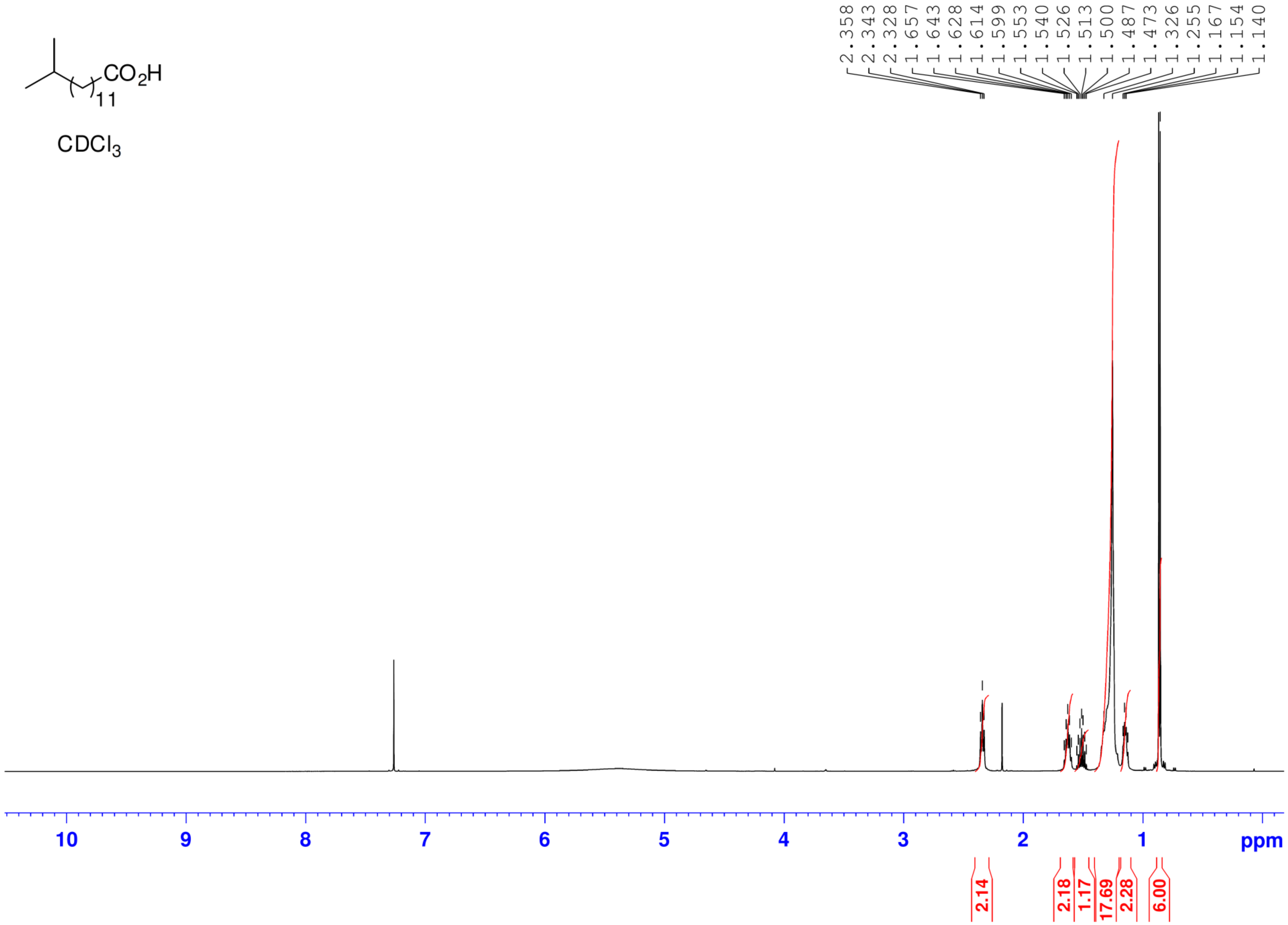

^{13}C NMR (125 MHz, $CDCl_3$) – 13-Methyltetradecanoic acid (**4**)

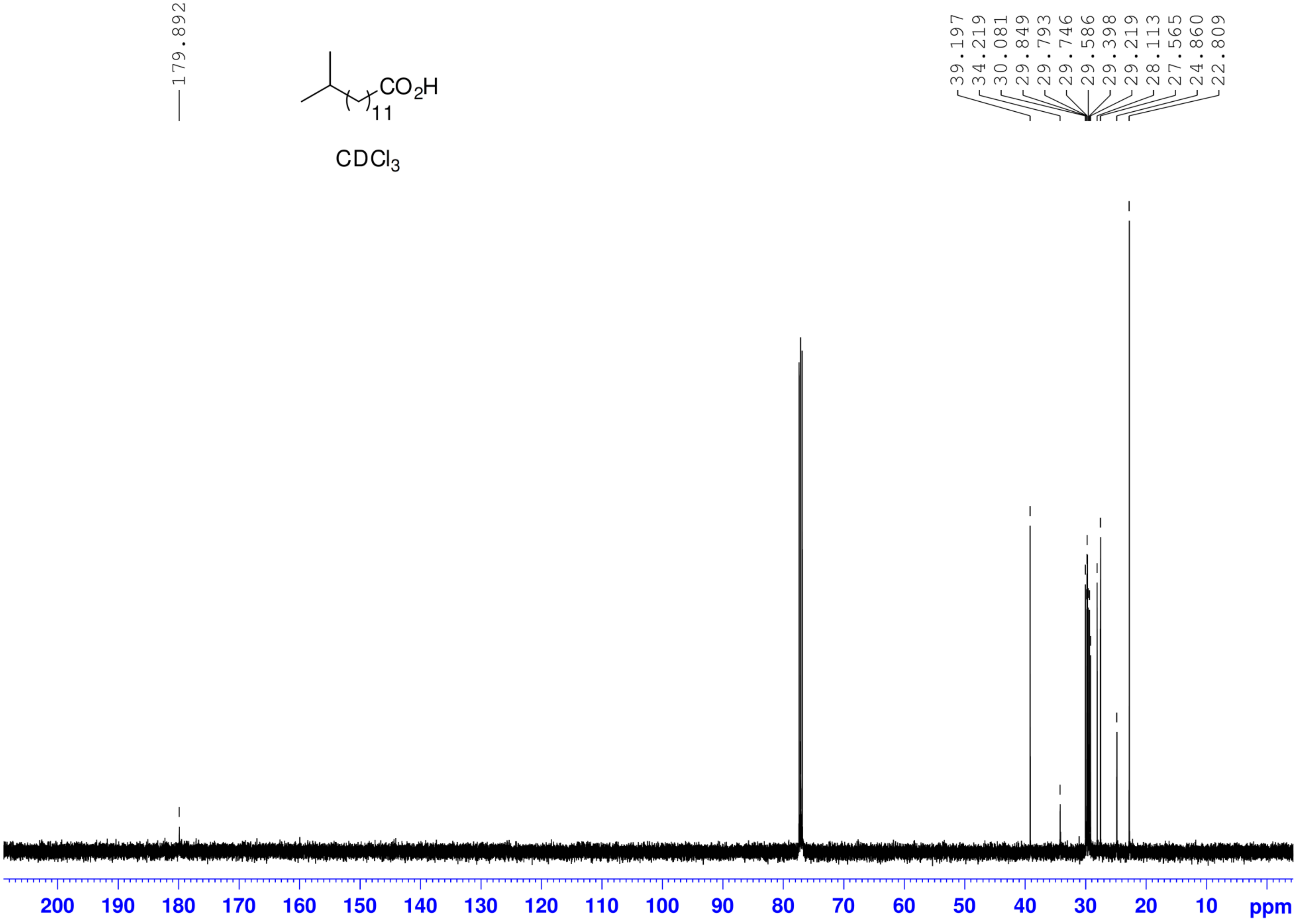

^{1}H NMR (500 MHz, $CDCl_3$) – 14-Methylpentadecanoic acid (**5**)

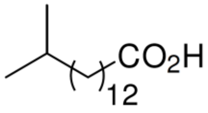

$CDCl_3$

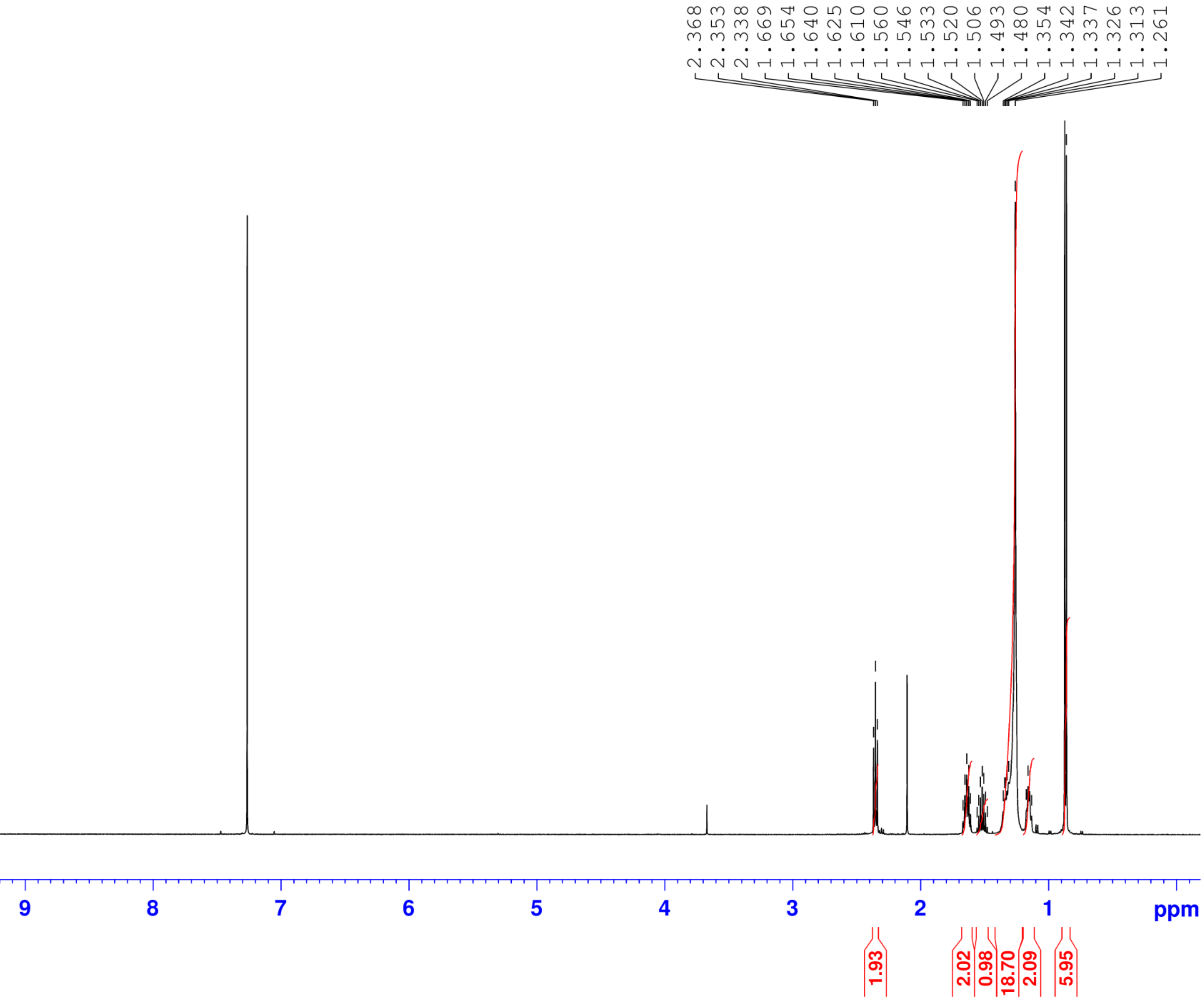

^{13}C NMR (125 MHz, $CDCl_3$) – 14-Methylpentadecanoic acid (**5**)

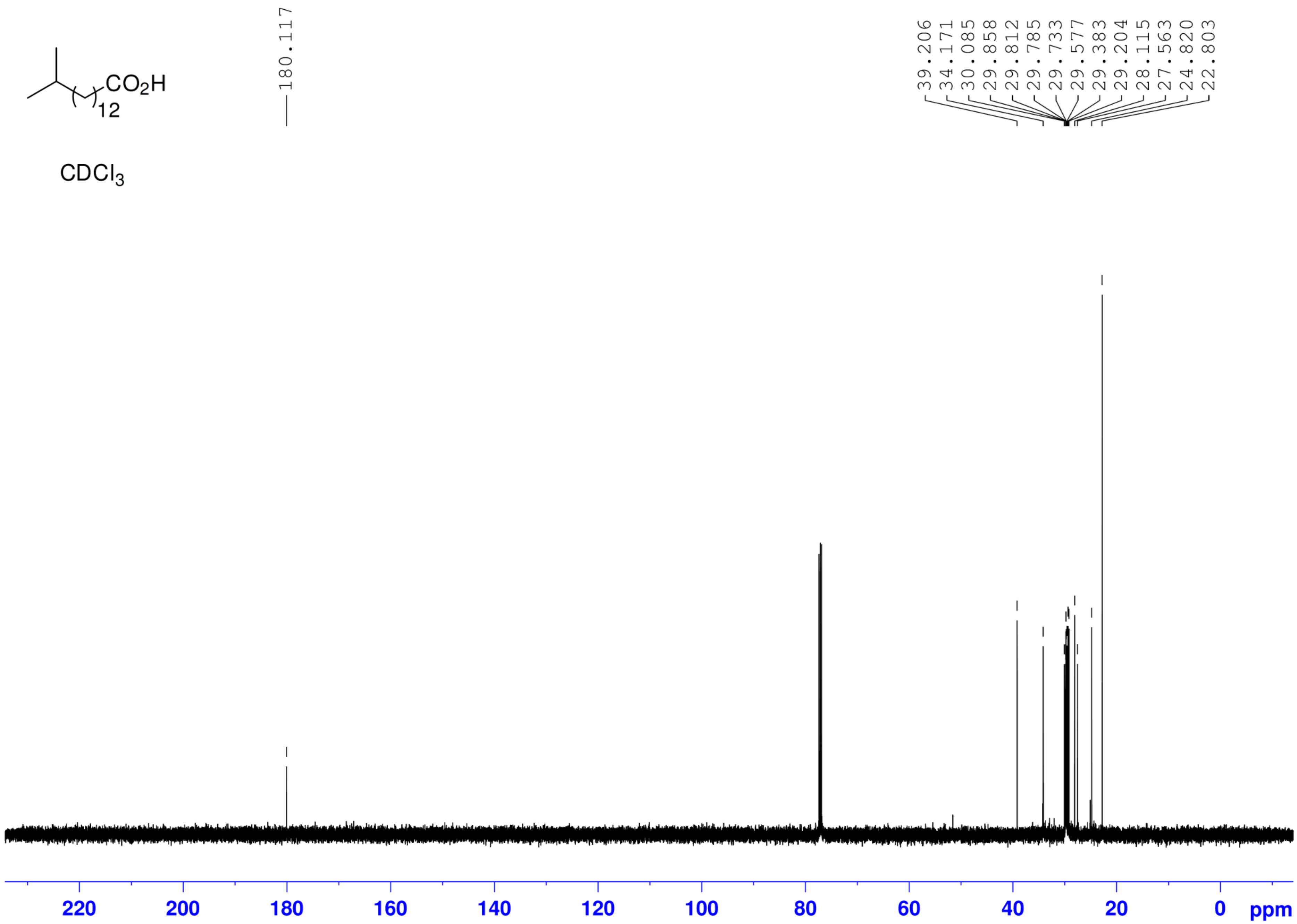

^{1}H NMR (500 MHz, $CDCl_3$) – 15-Methylhexadecanoic acid (**6**)

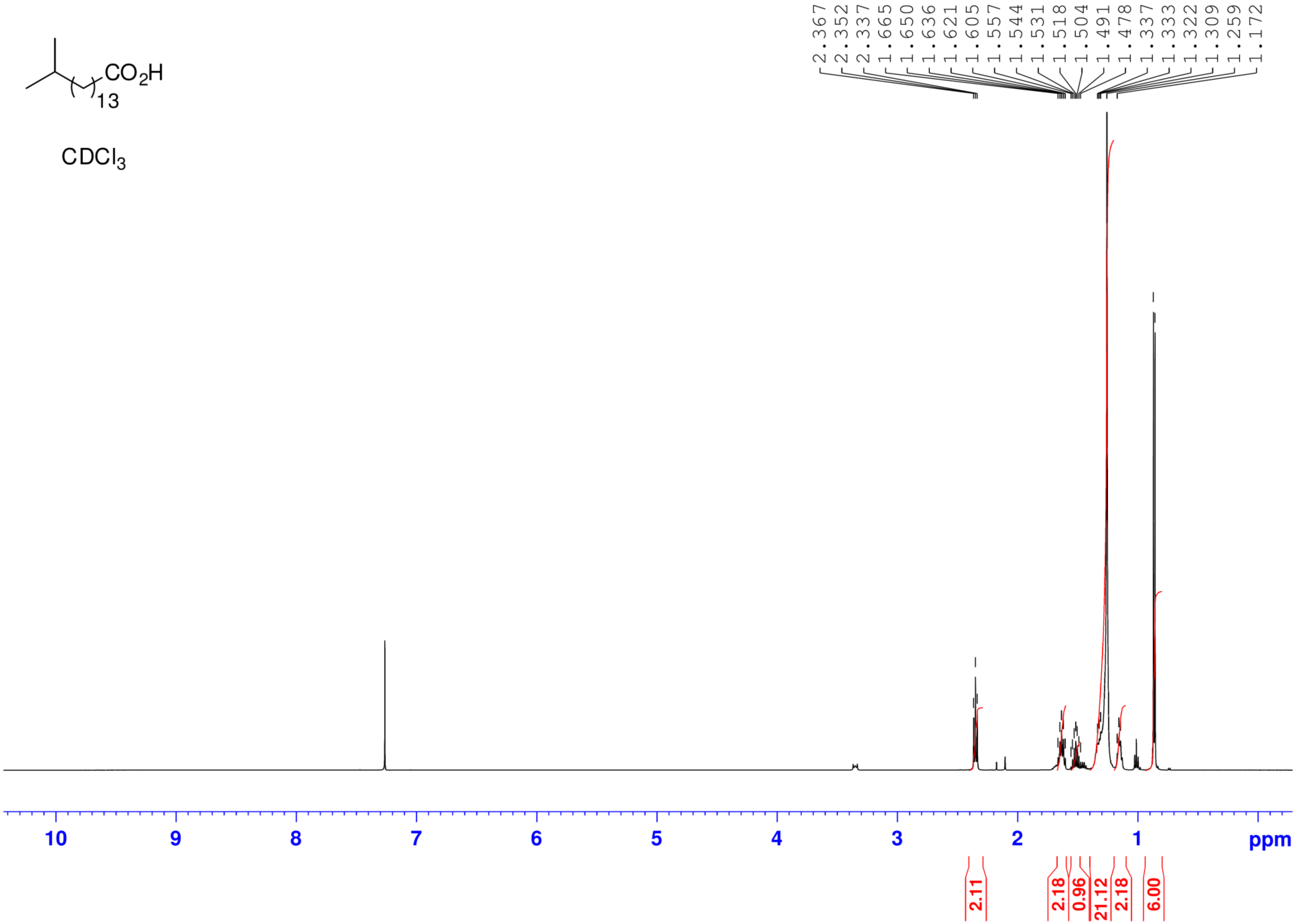

^{13}C NMR (125 MHz, $CDCl_3$) – 15-Methylhexadecanoic acid (**6**)

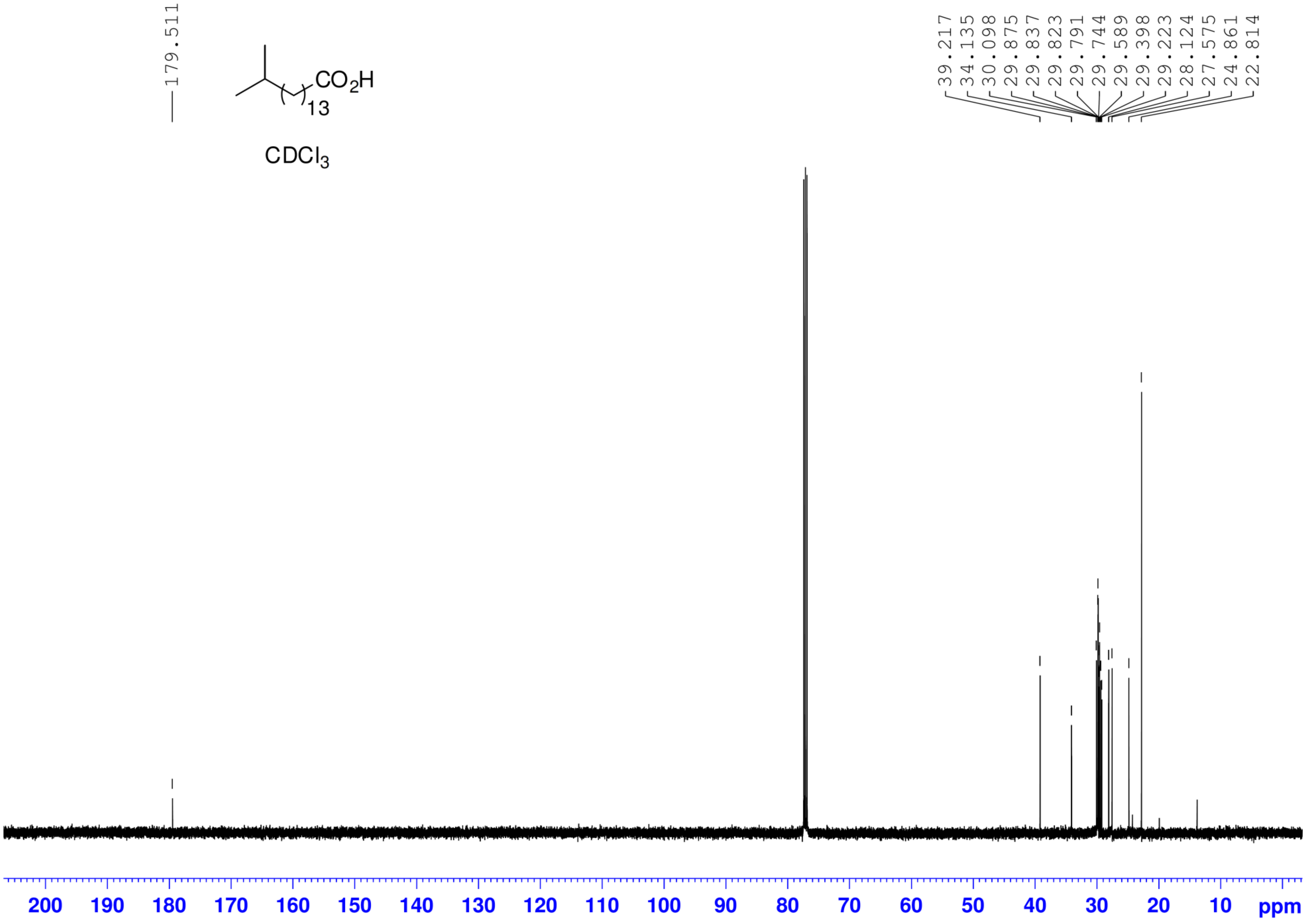

^{1}H NMR (500 MHz, $CDCl_3$) – 16-Methylheptadecanoic acid (**7**)

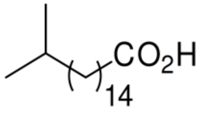

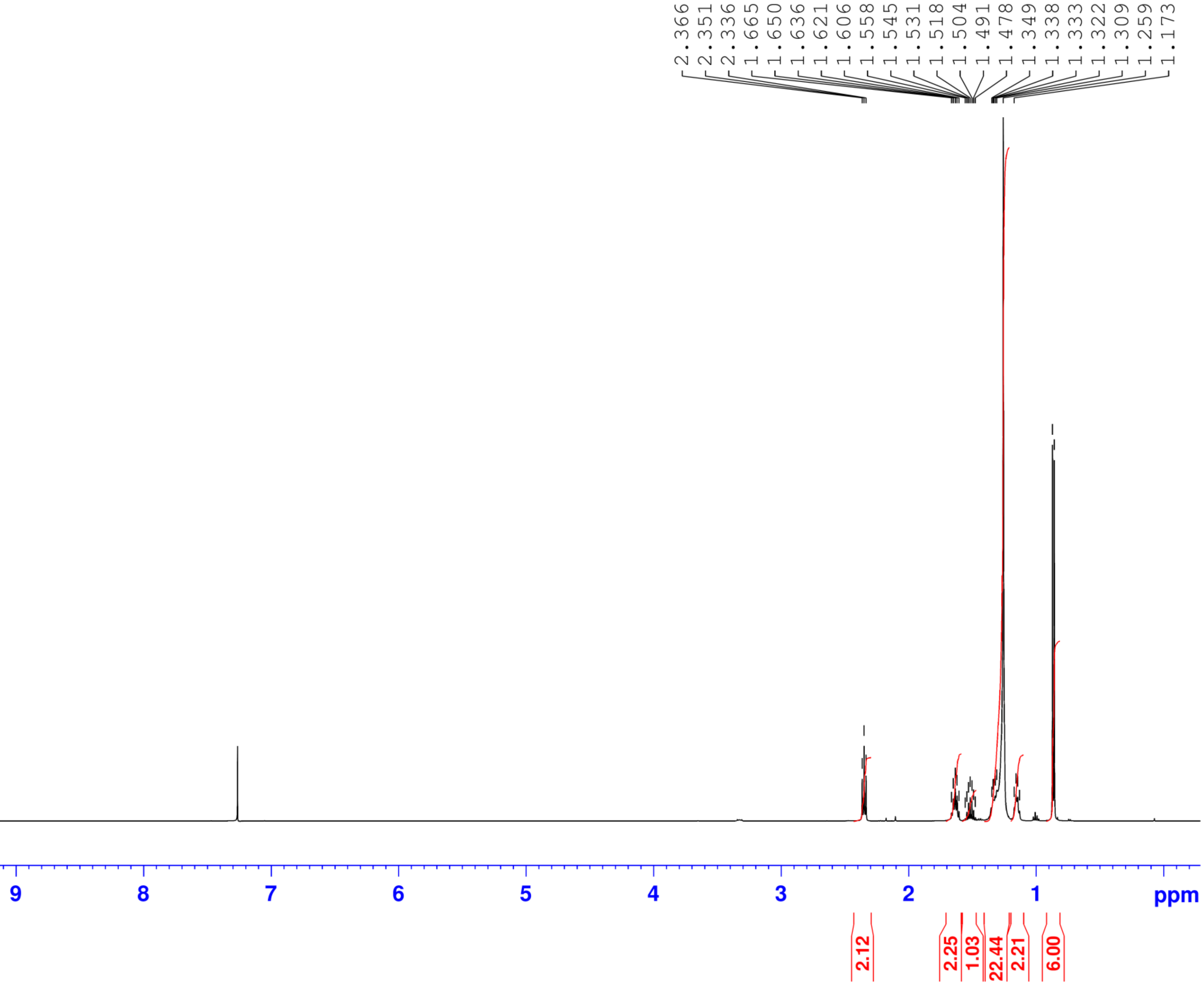

^{13}C NMR (125 MHz, $CDCl_3$) – 16-Methylheptadecanoic acid (**7**)

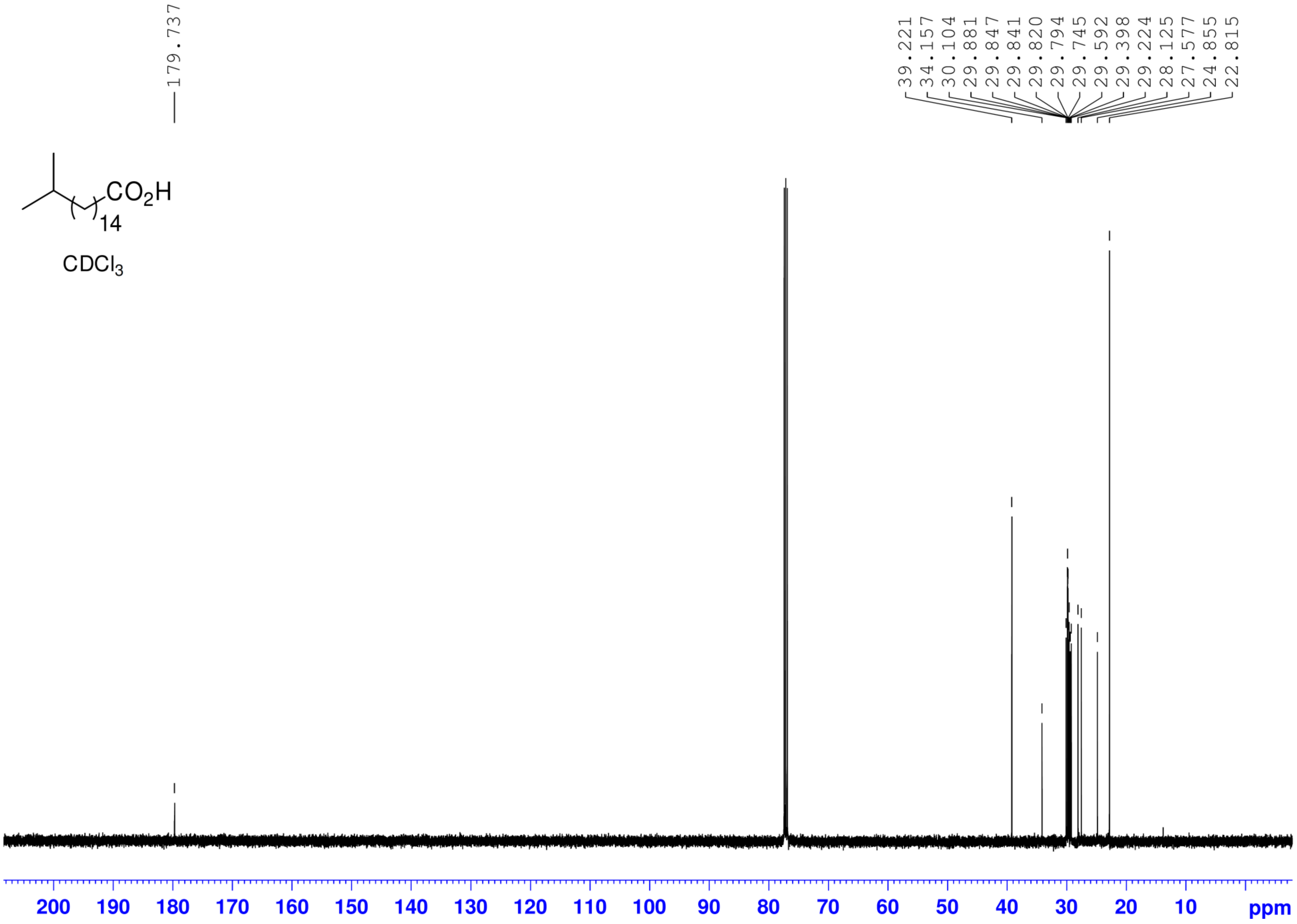

^{1}H NMR (500 MHz, $CDCl_3$) – 17-Methyloctatadecanoic acid (**8**)

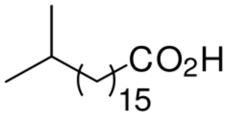

$CDCl_3$

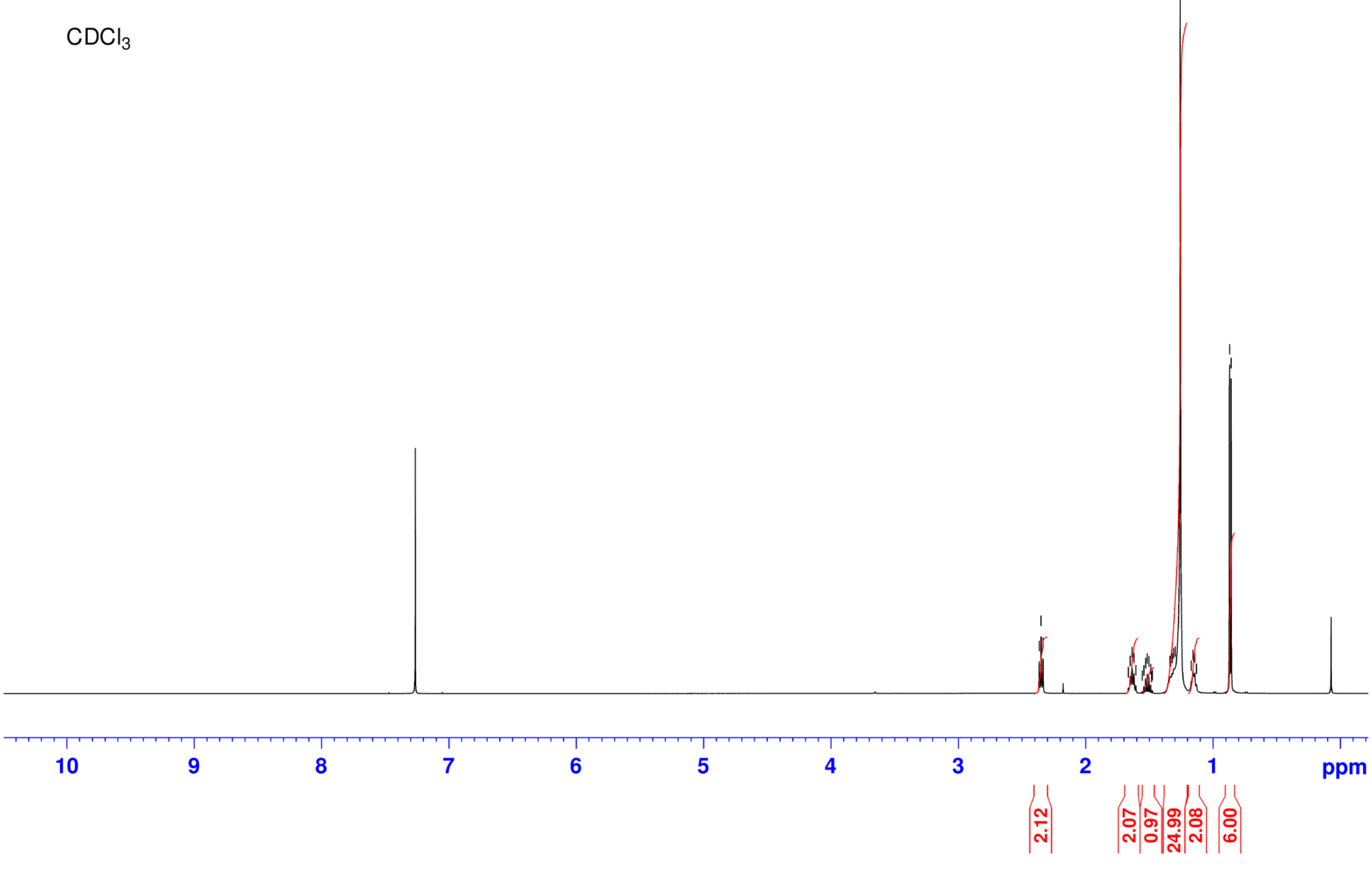

^{13}C NMR (125 MHz, $CDCl_3$) – 17-Methyloctatadecanoic acid (**8**)

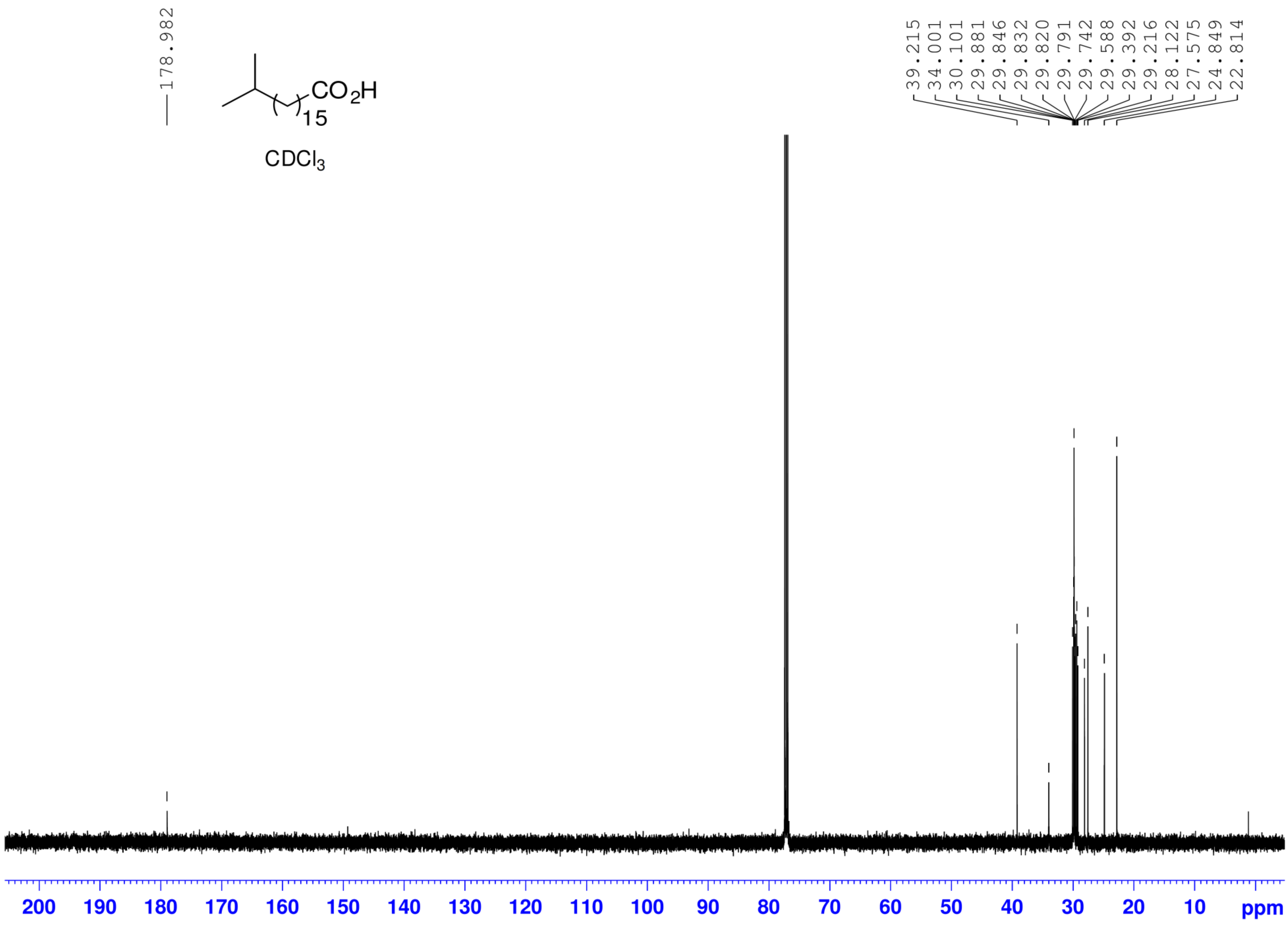

^{1}H NMR (500 MHz, $CDCl_3$) – 2-Methyldodec-11-en-2-ol (**10**)

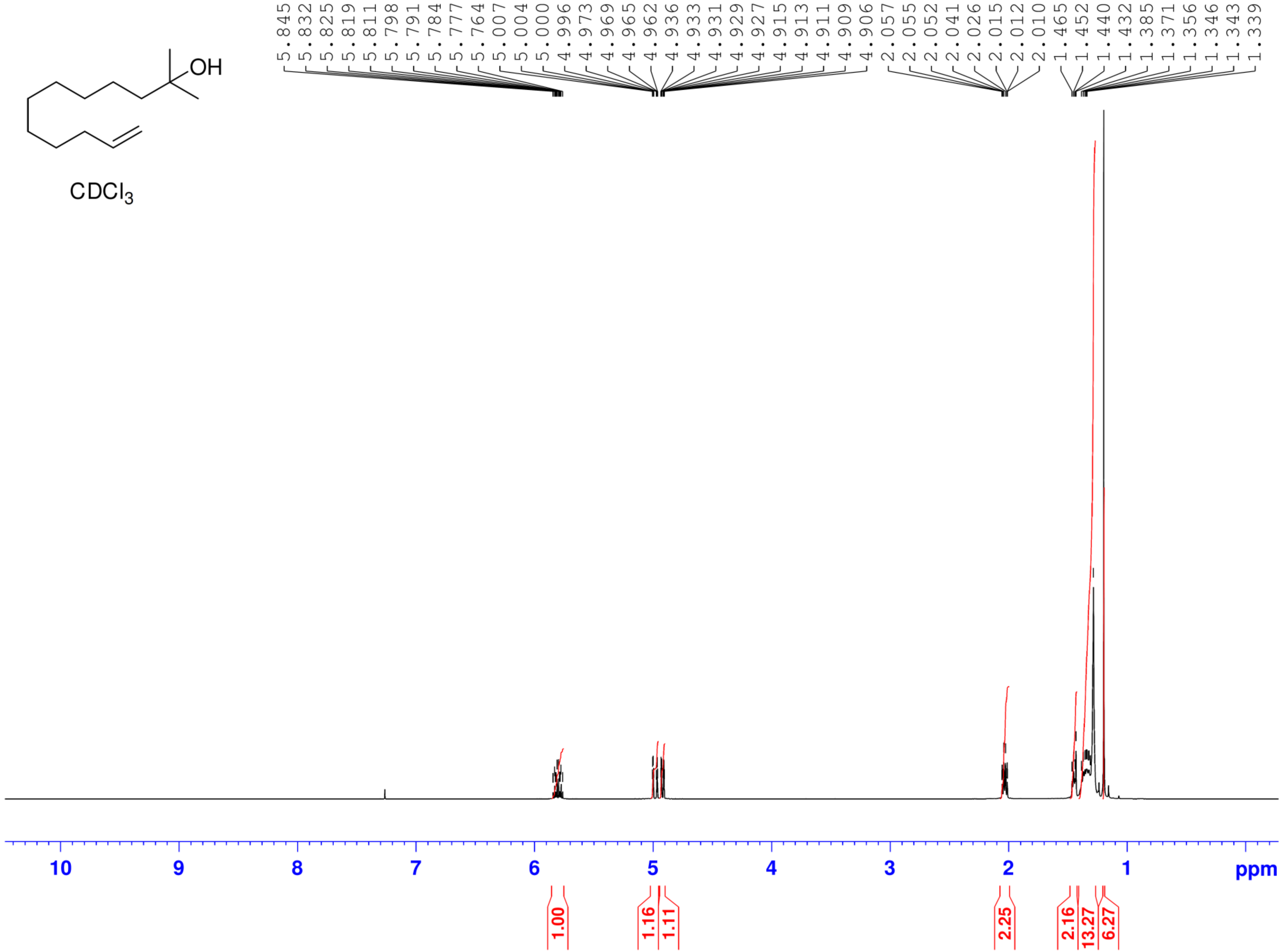

^{13}C NMR (125 MHz, $CDCl_3$) – 2-Methyldodec-11-en-2-ol (**10**)

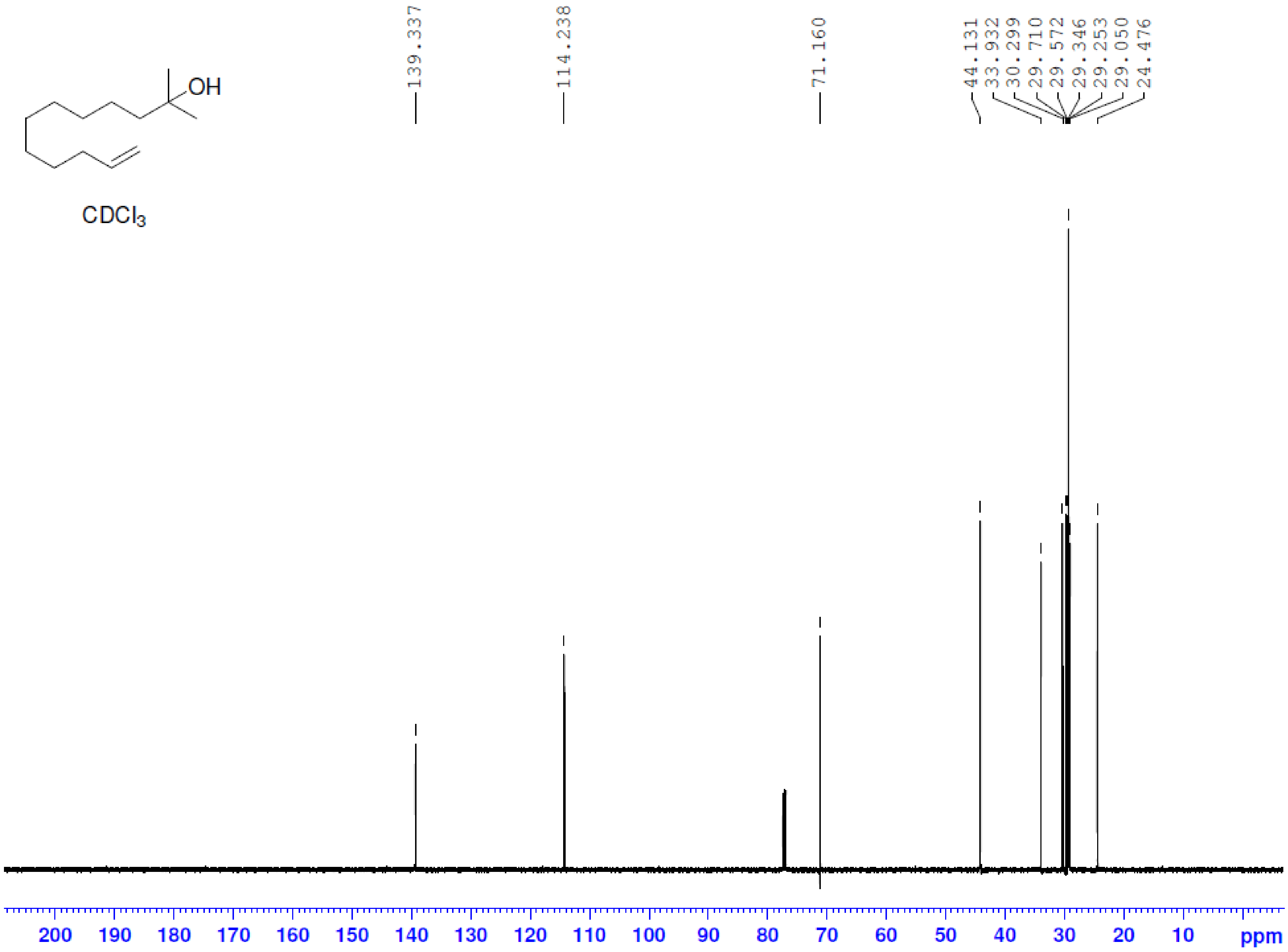

^{1}H NMR (500 MHz, $CDCl_3$) – 11-Methyldodec-1-ene (**11**)

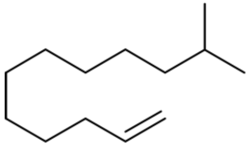

$CDCl_3$

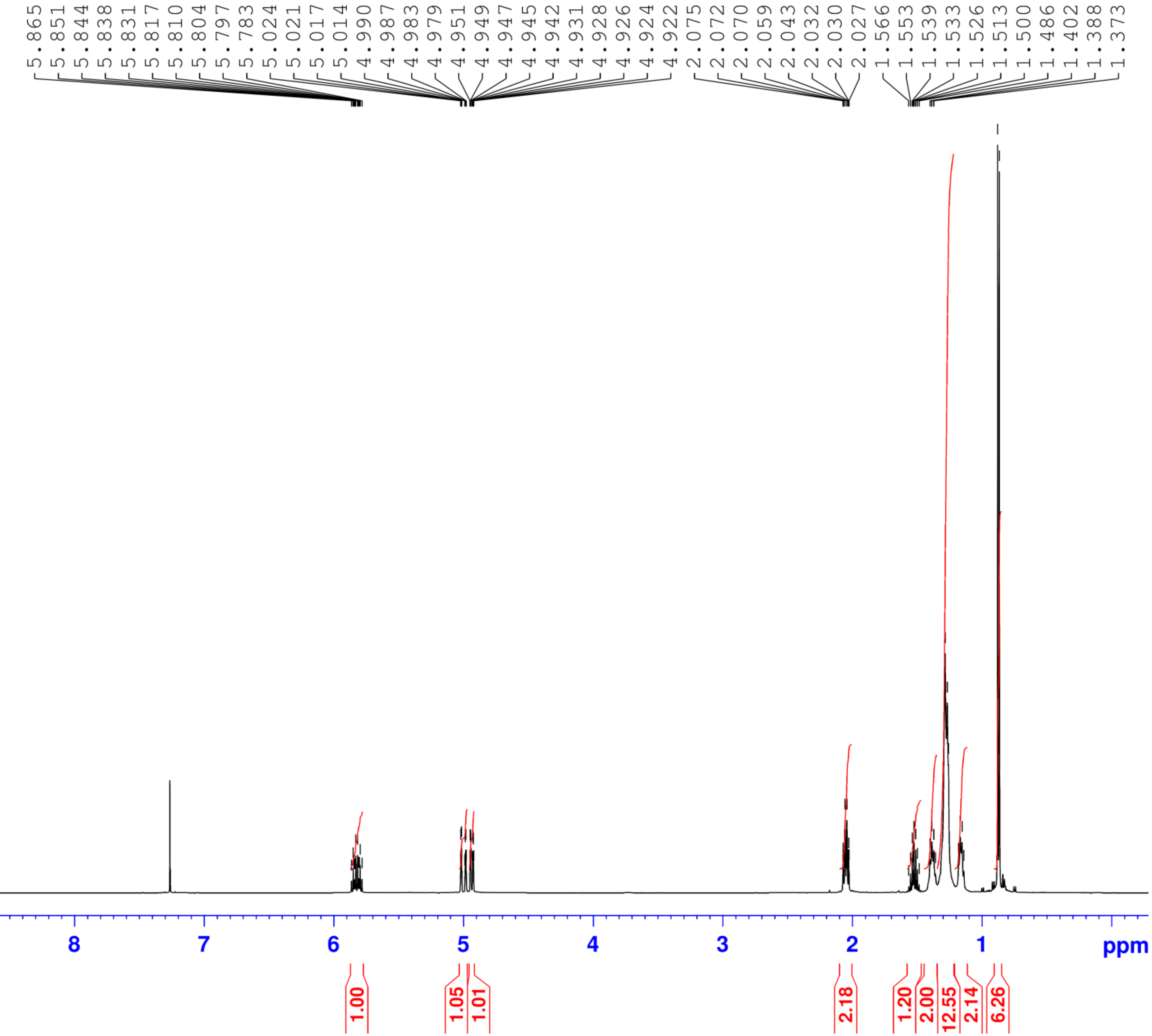

^{13}C NMR (125 MHz, $CDCl_3$) – 11-Methyldodec-1-ene (**11**)

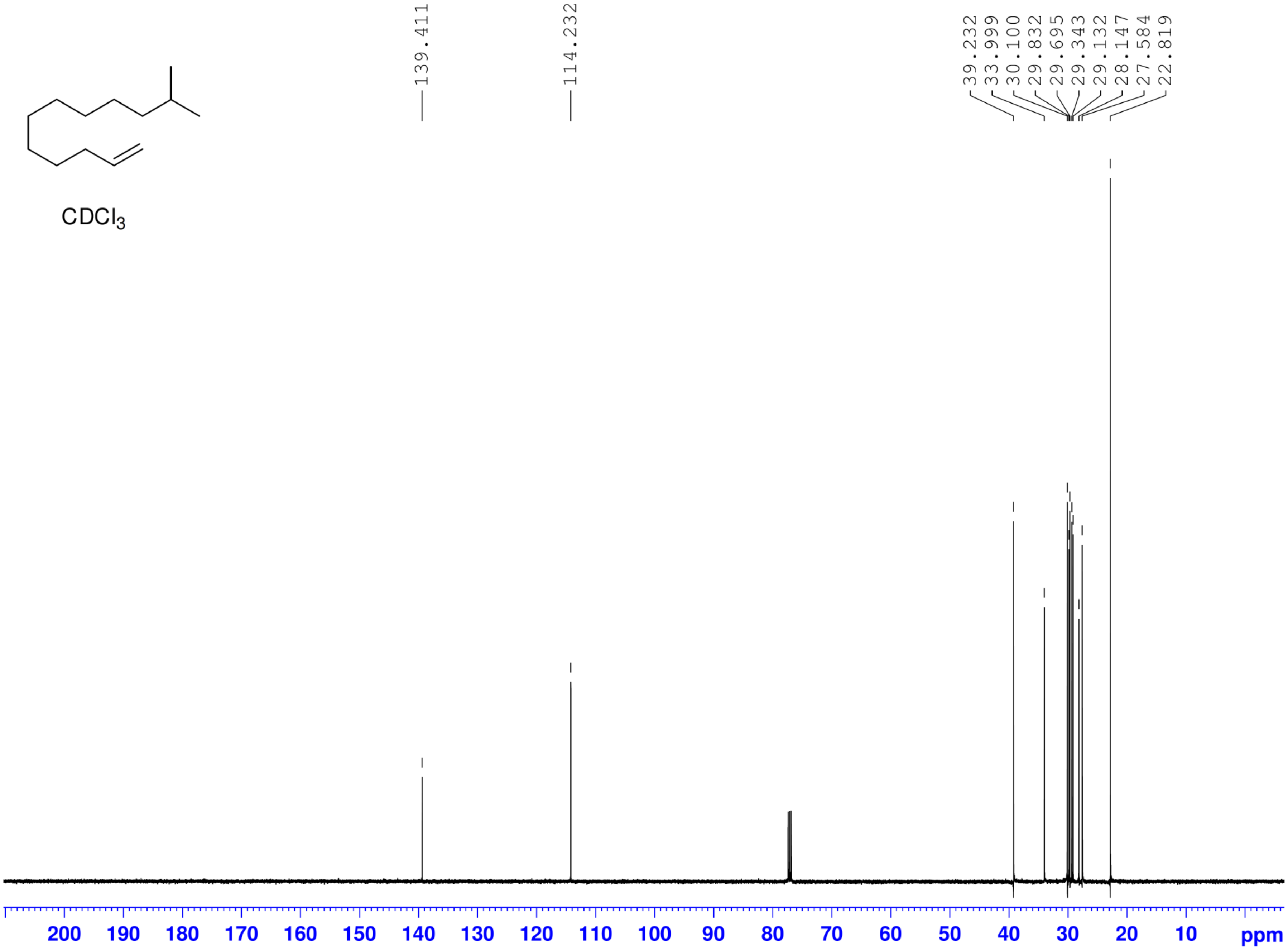

^{1}H NMR (500 MHz, $CDCl_3$) – 11-Methyldodecan-1-ol (**12**)

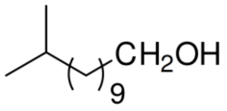

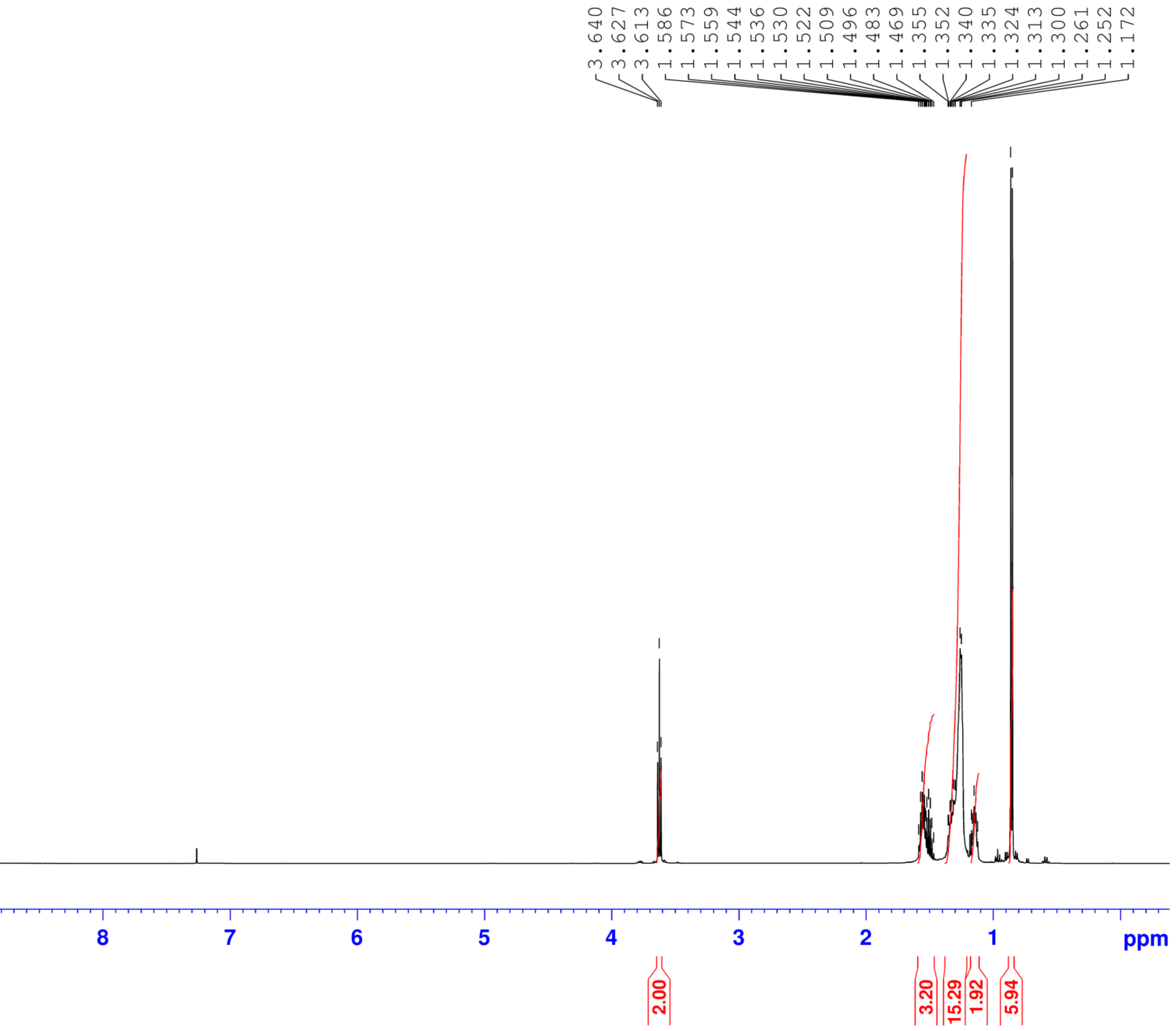

^{13}C NMR (125 MHz, $CDCl_3$) – 11-Methyldodecan-1-ol (**12**)

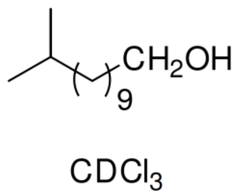

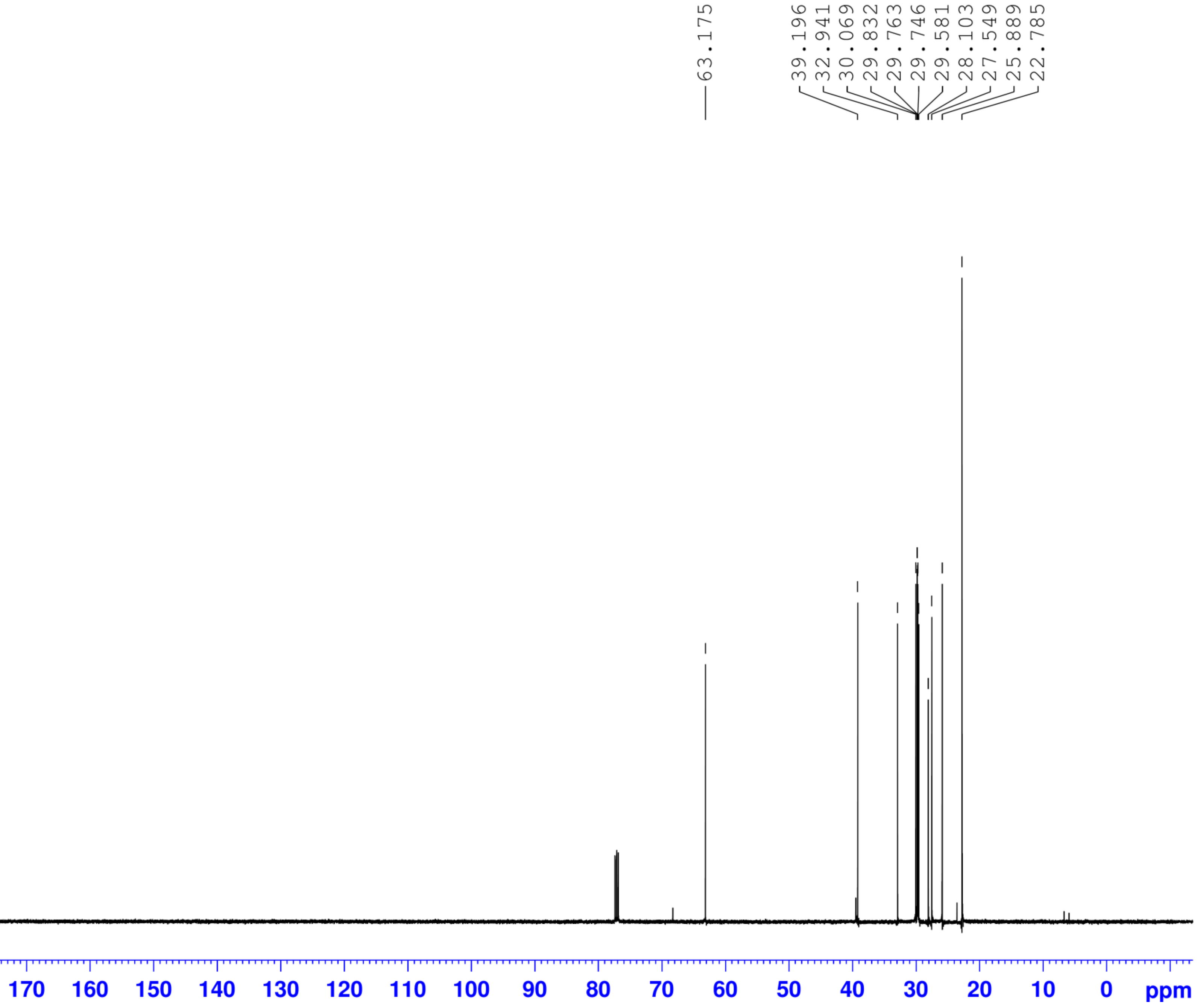

^{1}H NMR (500 MHz, $CDCl_3$) – 11-Methyldodecyl methanesulfonate (**13**)

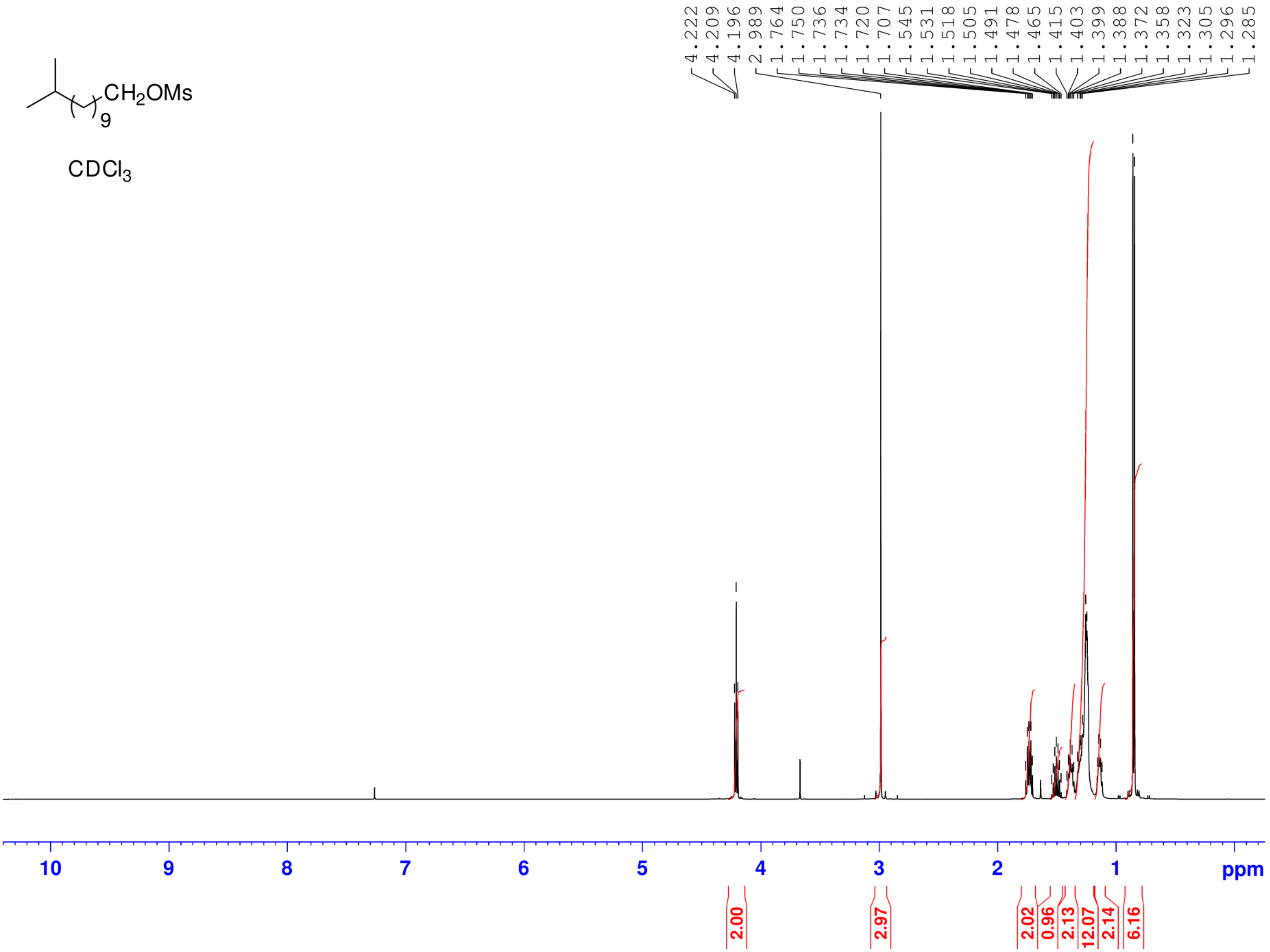

^{13}C NMR (125 MHz, $CDCl_3$) – 11-Methyldodecyl methanesulfonate (**13**)

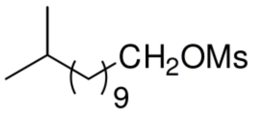

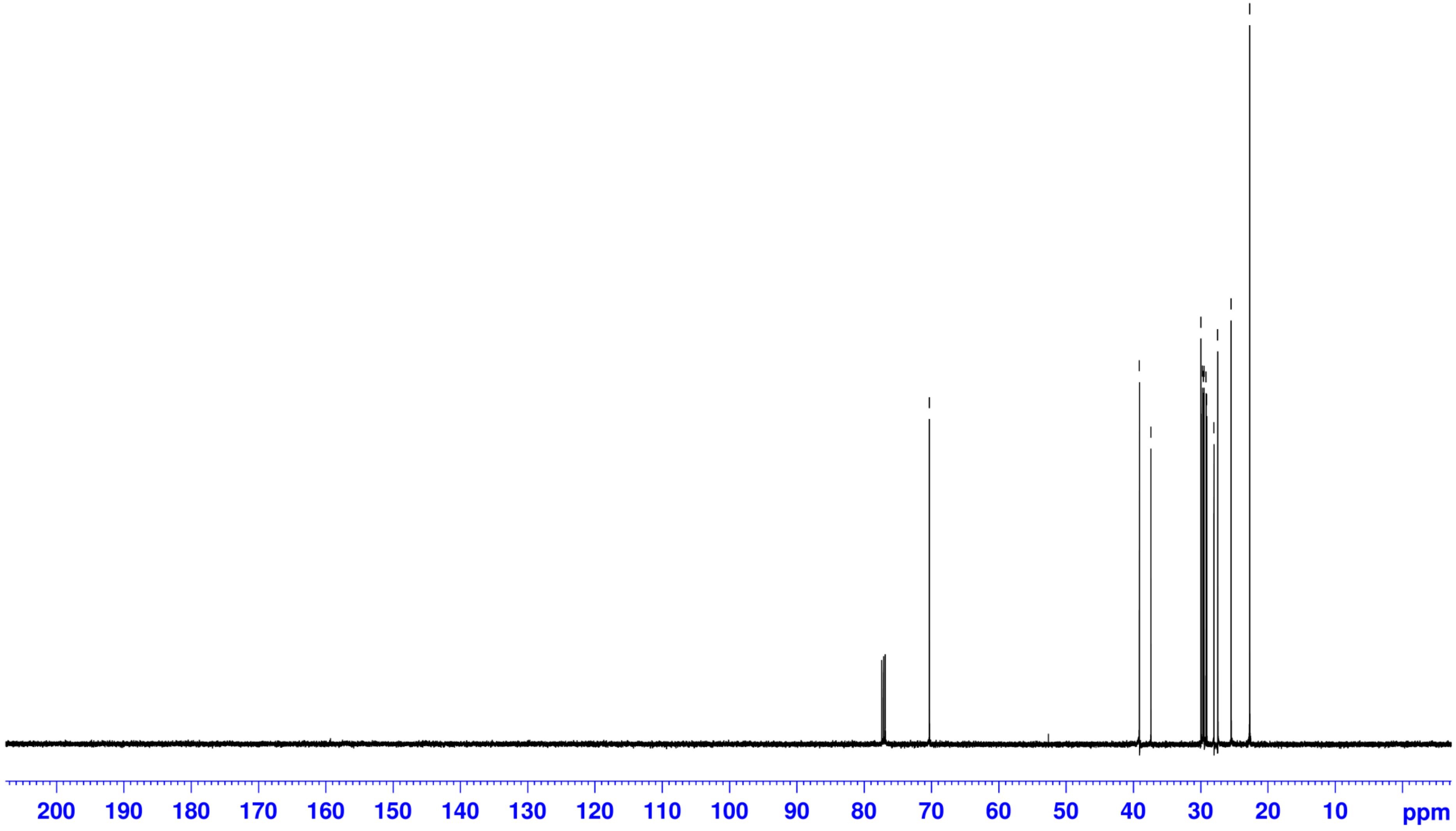

^{1}H NMR (500 MHz, $CDCl_3$) – 12-Methyltridecanenitrile (**14**)

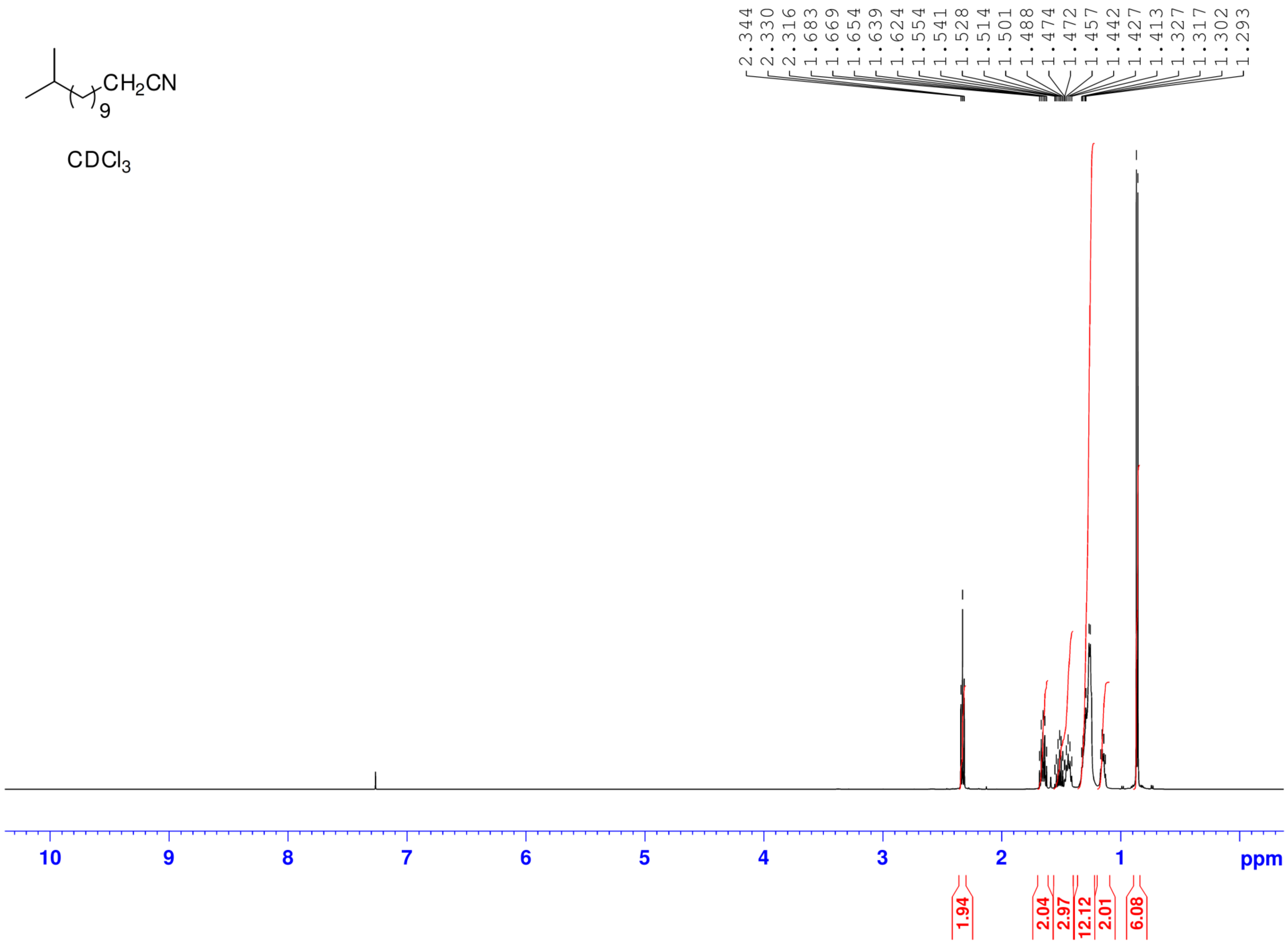

^{13}C NMR (125 MHz, $CDCl_3$) – 12-Methyltridecanenitrile (**14**)

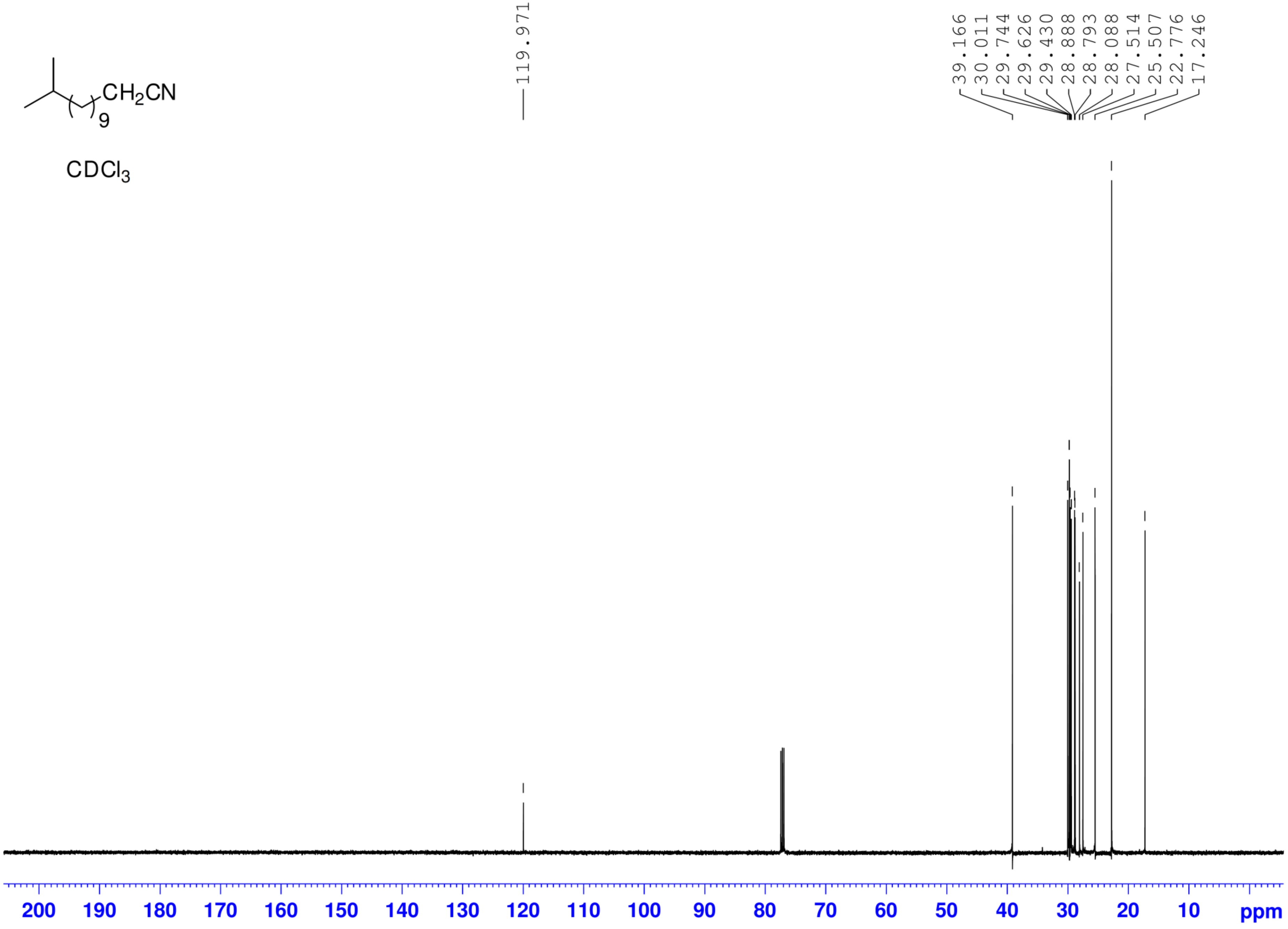

^{1}H NMR (500 MHz, DMSO-d_6) – 15-Methylhexadecane-1,15-diol (**16**)

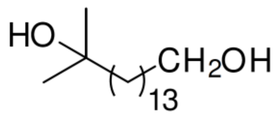

DMSO

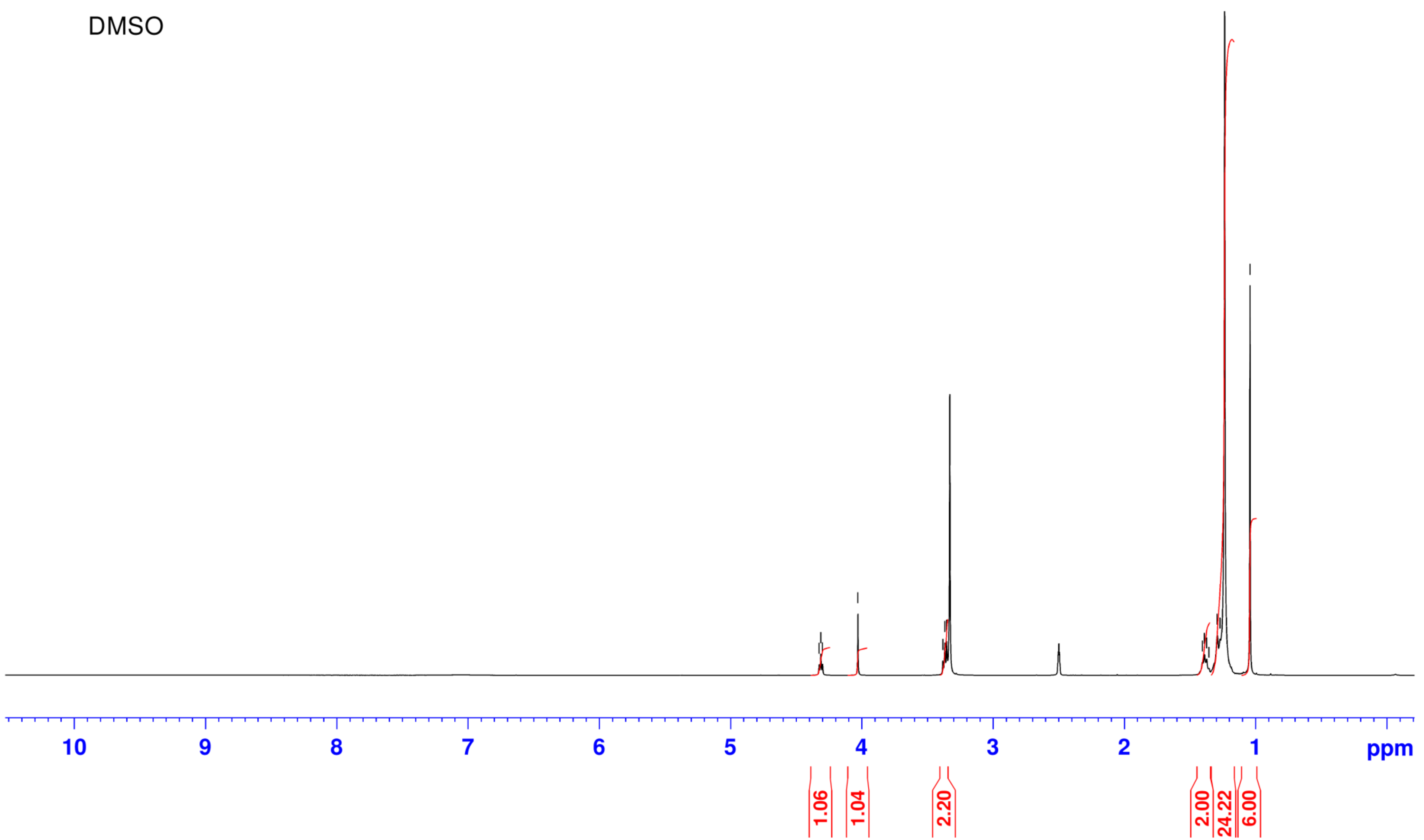

^{13}C NMR (125 MHz, $CDCl_3$) – 15-Methylhexadecane-1,15-diol (**16**)

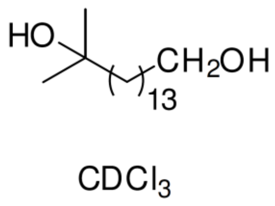

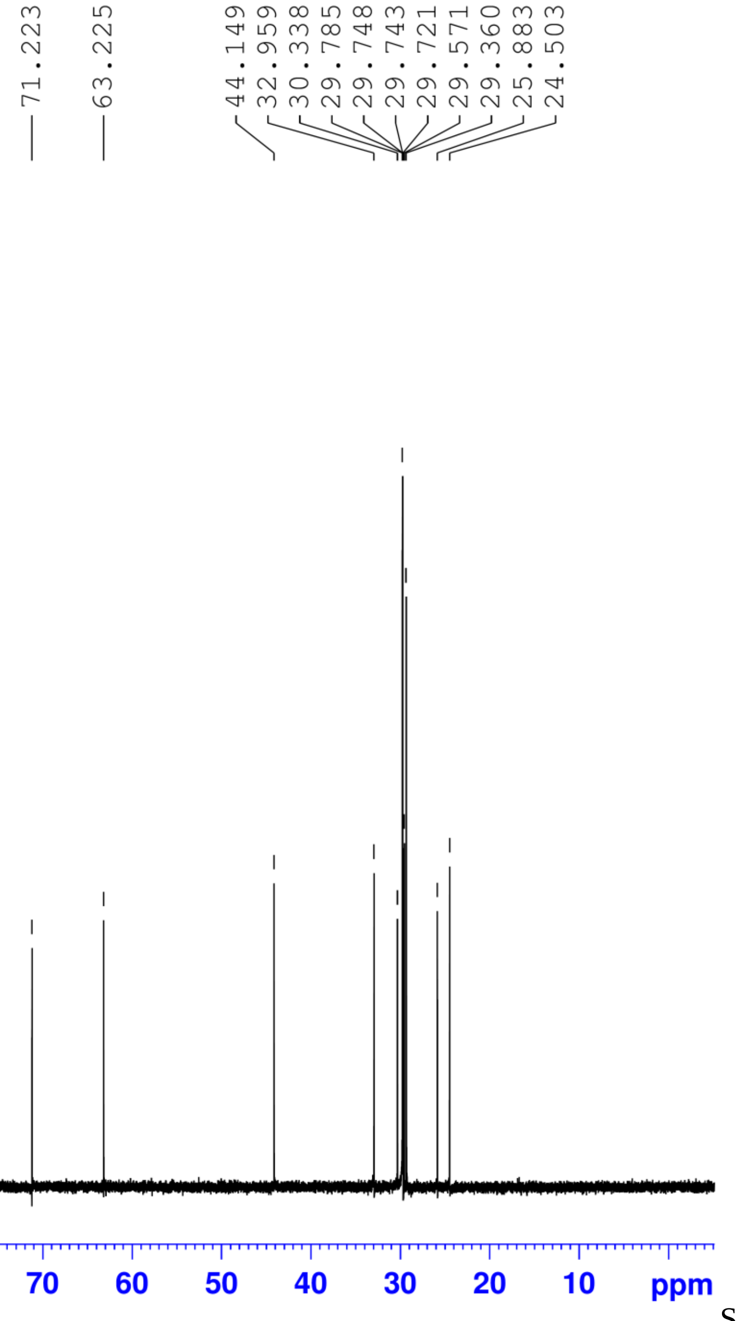

^{1}H NMR (500 MHz, $CDCl_3$) – 15-Methylhexadecan-1-ol (**17**)

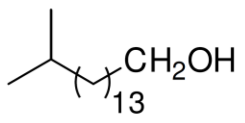

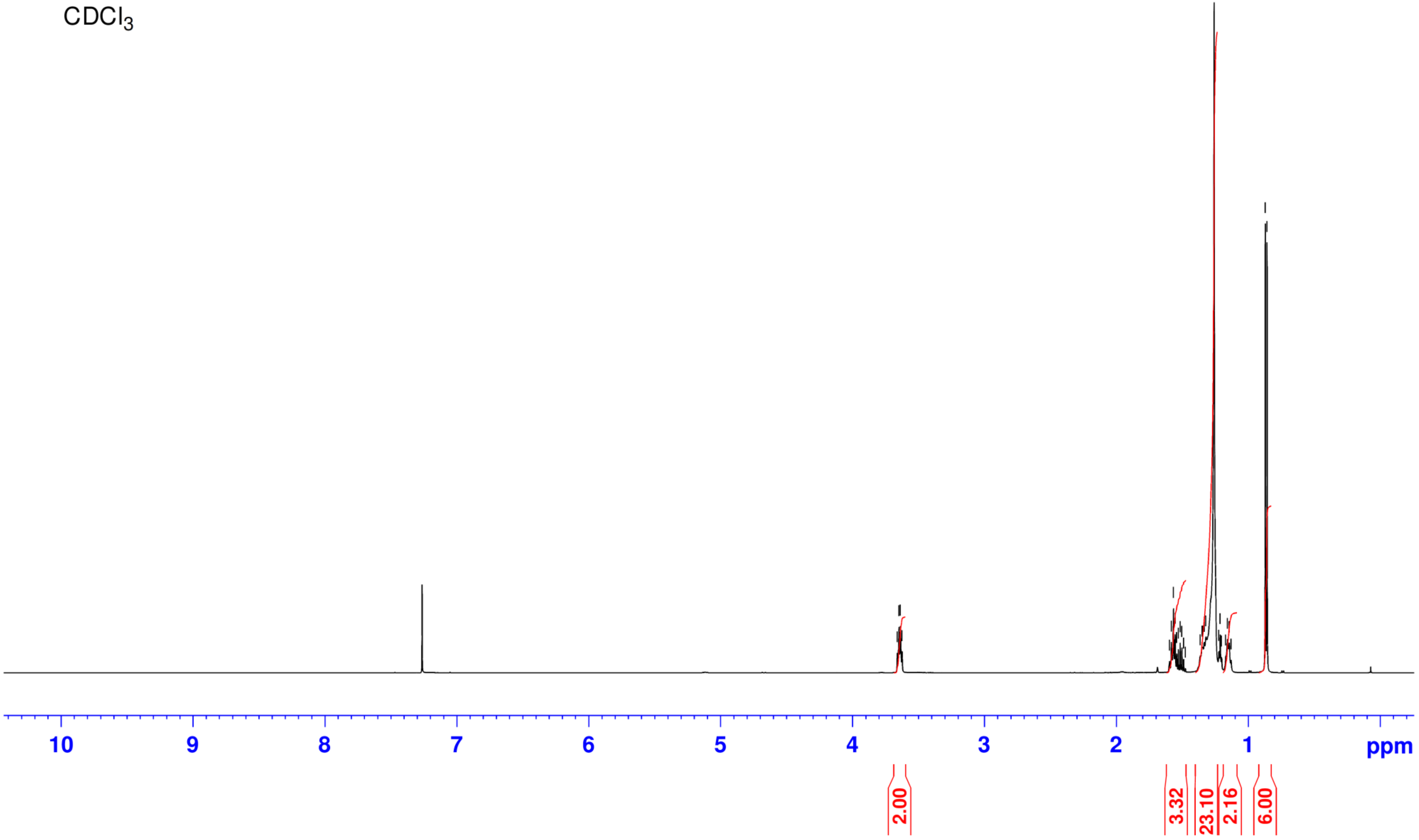

^{13}C NMR (125 MHz, $CDCl_3$) – 15-Methylhexadecan-1-ol (**17**)

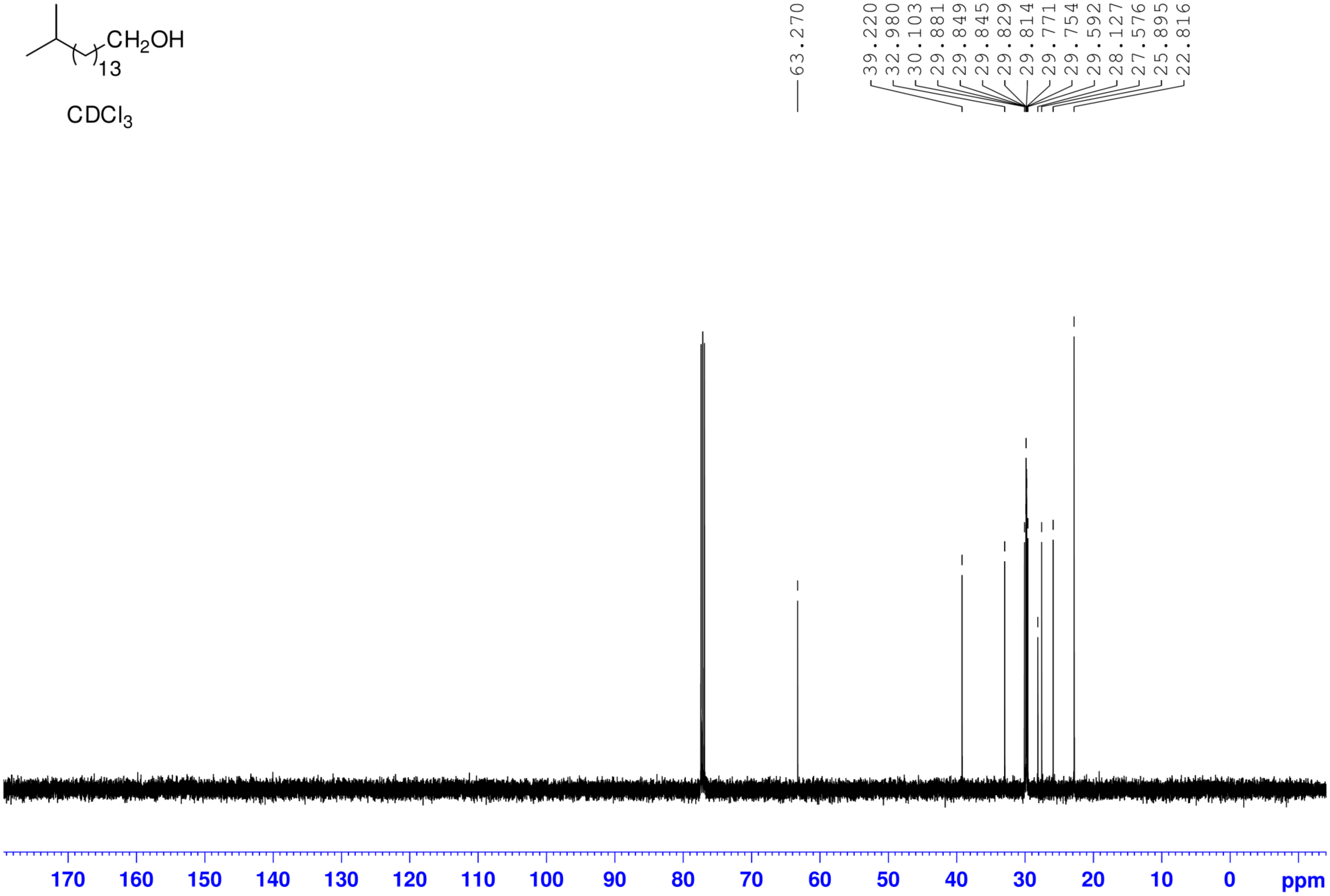

^{1}H NMR (500 MHz, $CDCl_3$) – *S*-Methyl-*O*-15-methylhexadecyl dithiocarbonate (**18**)

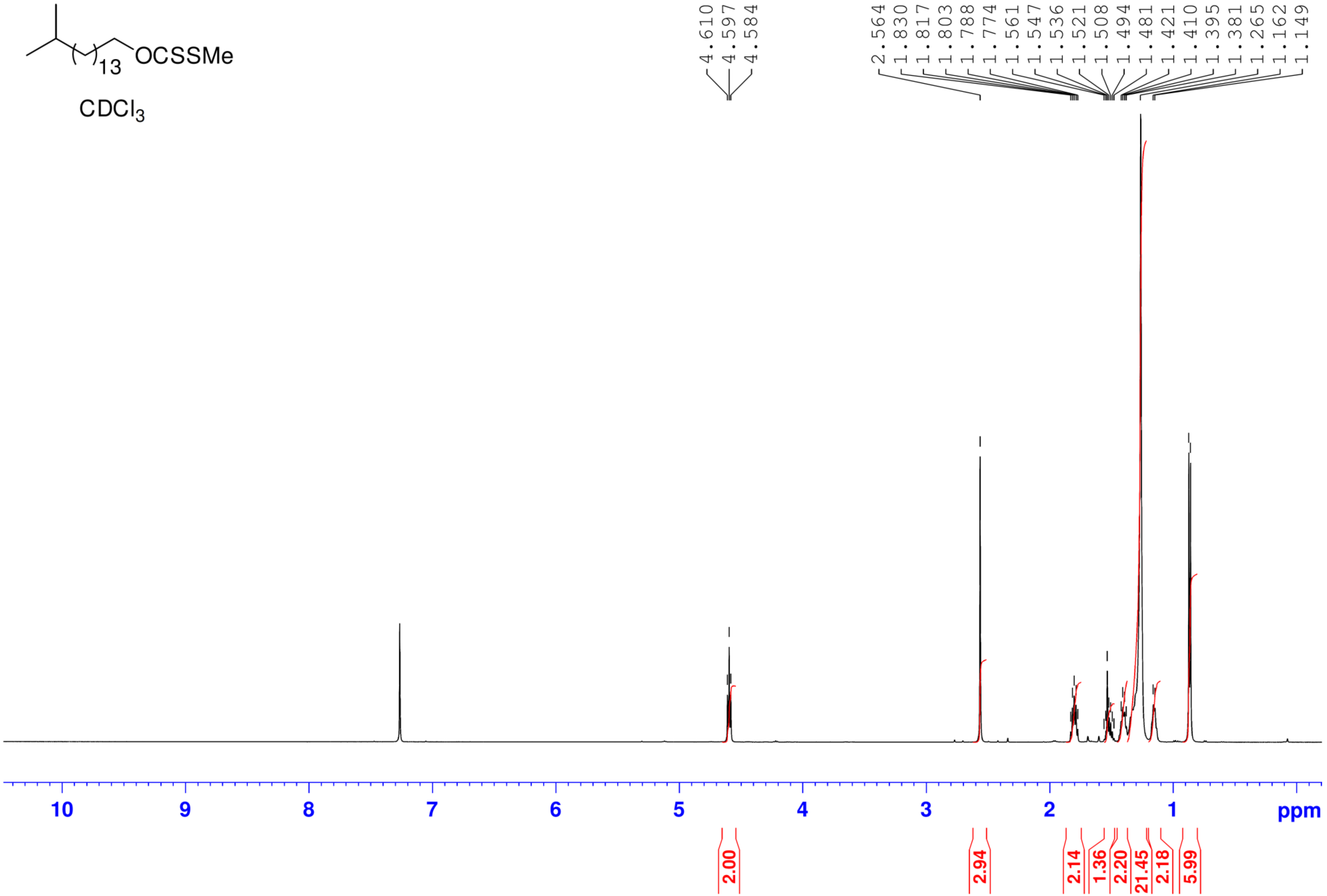

^{13}C NMR (125 MHz, $CDCl_3$) – *S*-Methyl-*O*-15-methylhexadecyl dithiocarbonate (**18**)

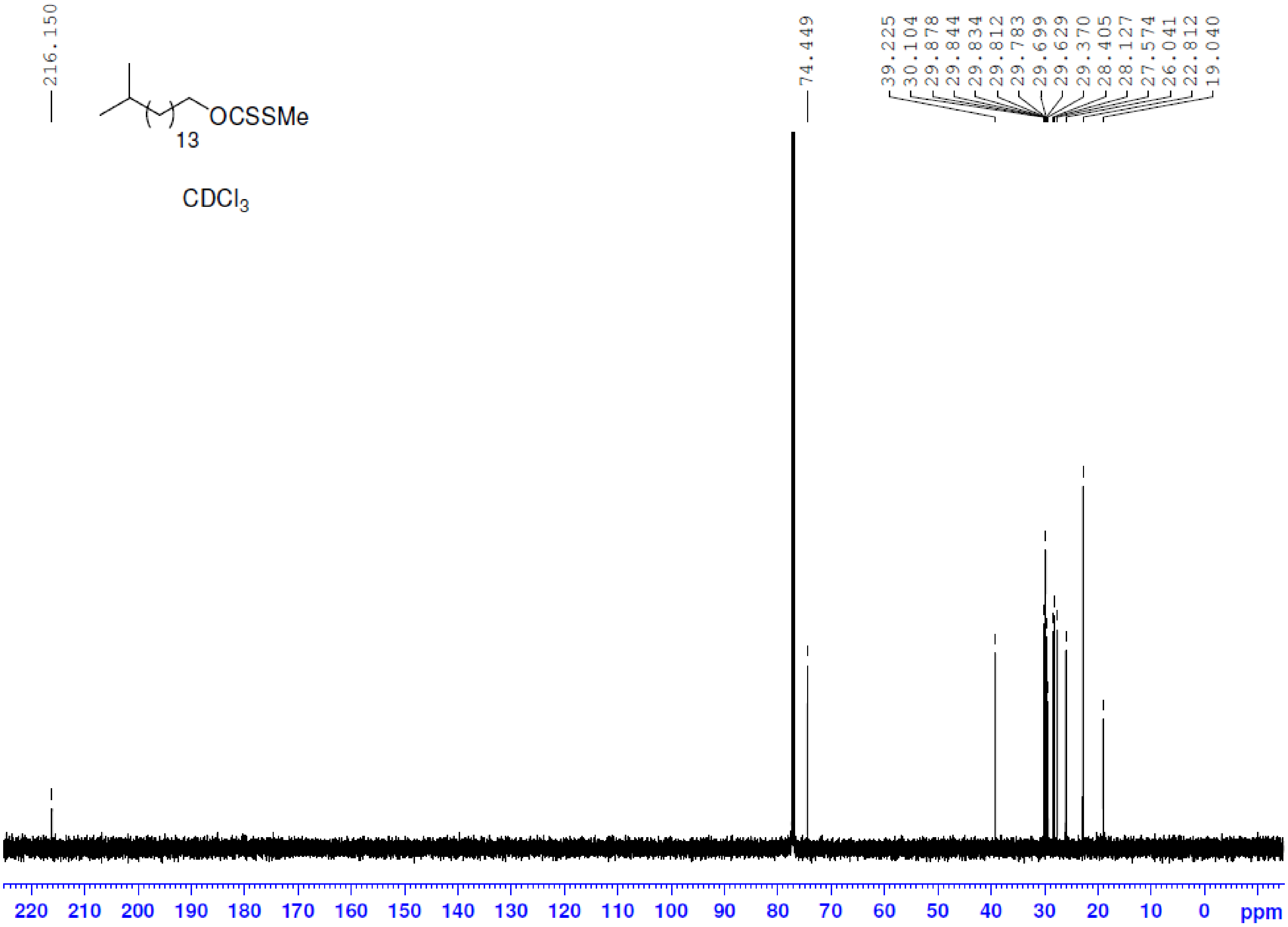

^{1}H NMR (500 MHz, $CDCl_3$) – 15-Methylhexadecan-1-ene (**19**)

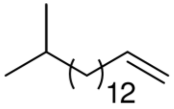

CDCl3

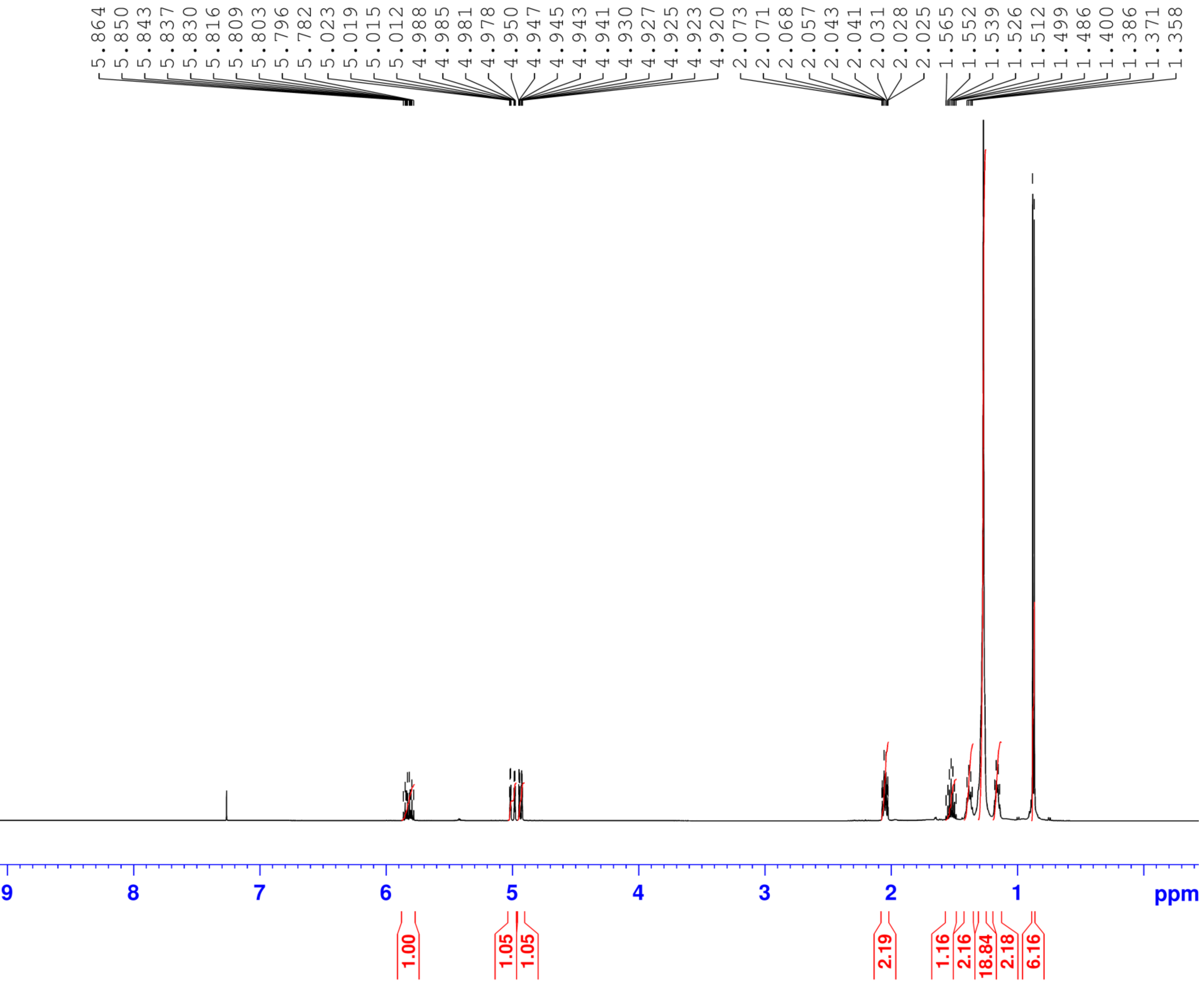

^{13}C NMR (125 MHz, $CDCl_3$) – 15-Methylhexadecan-1-ene (**19**)

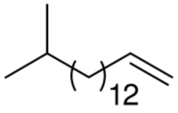

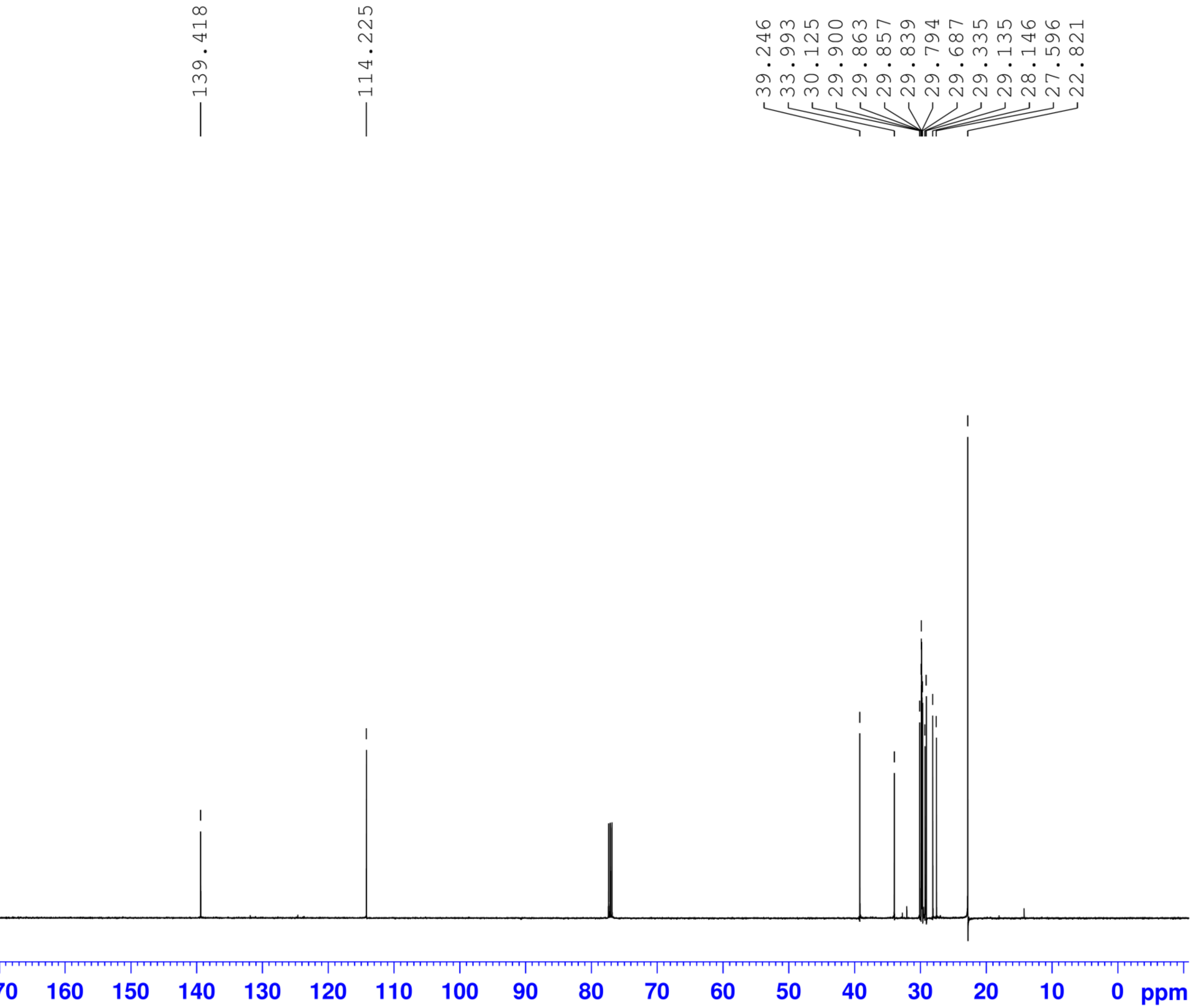

^1H NMR (500 MHz, $CDCl_3$) – 14-Methylpentadecan-1-ol (**20**)

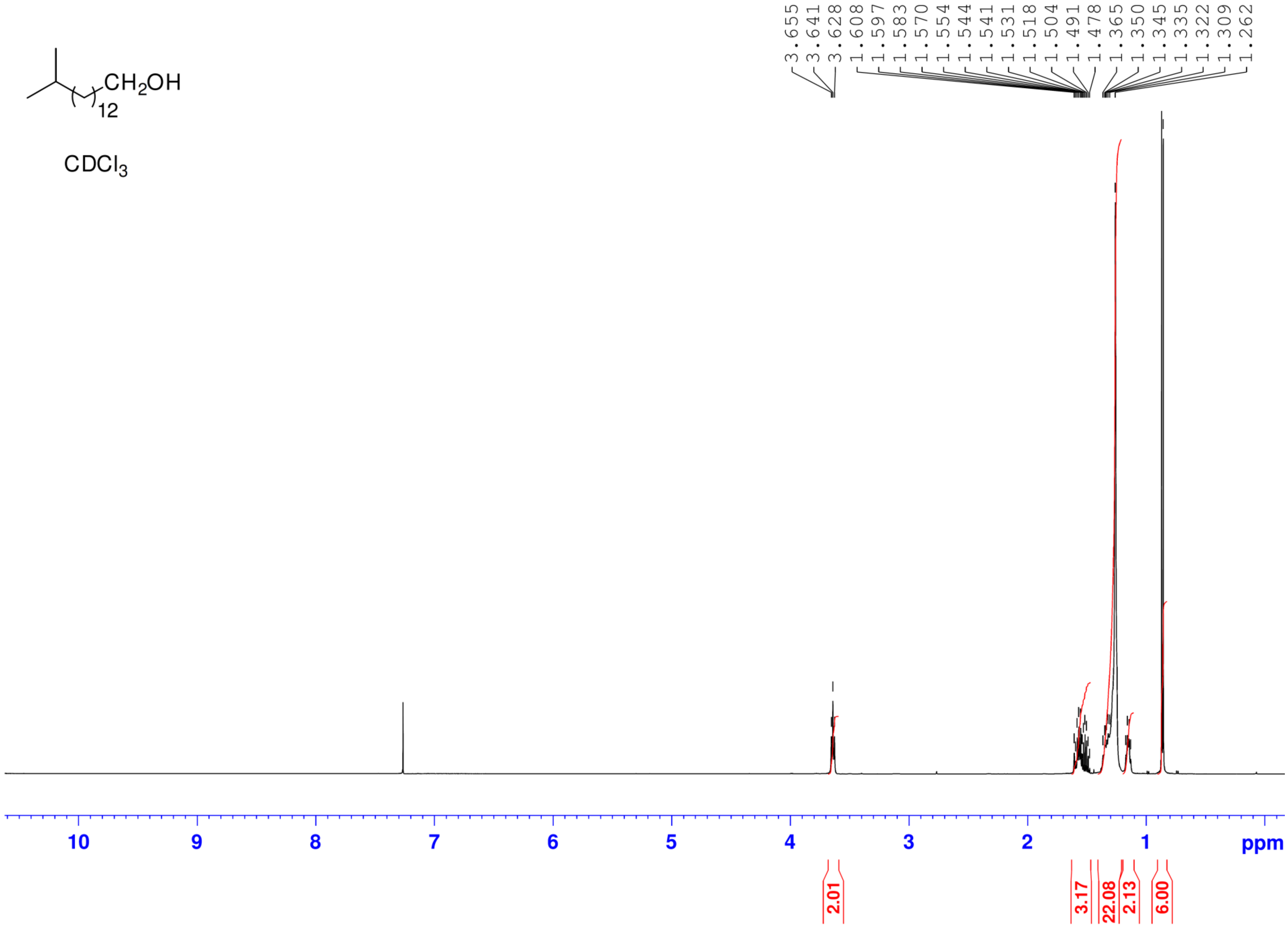

^{13}C NMR (125 MHz, $CDCl_3$) – 14-Methylpentadecan-1-ol (**20**)

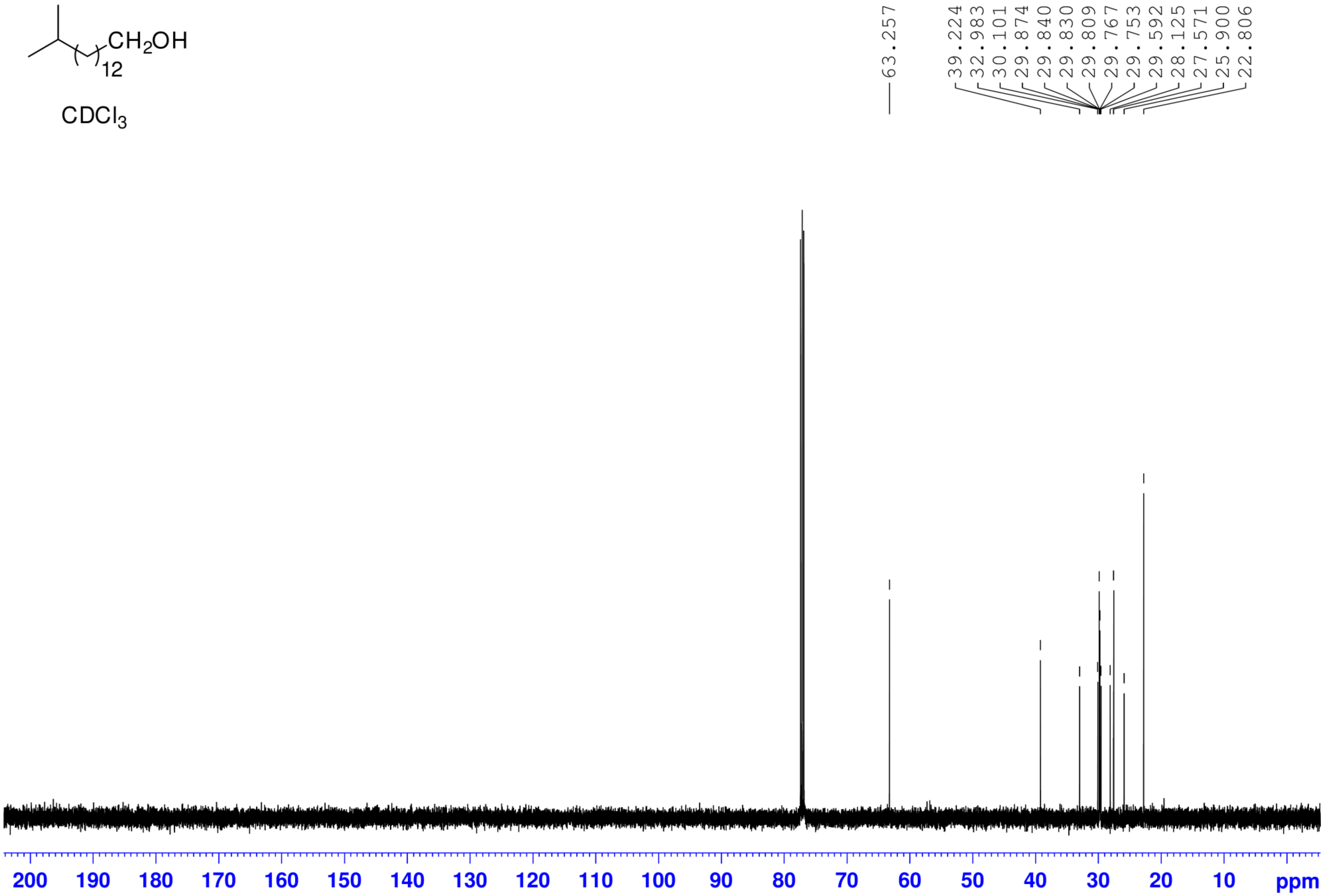

^{1}H NMR (500 MHz, $CDCl_3$) – 14-Methylpentadec-1-ene (**22**)

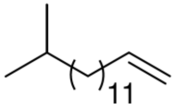

$CDCl_3$

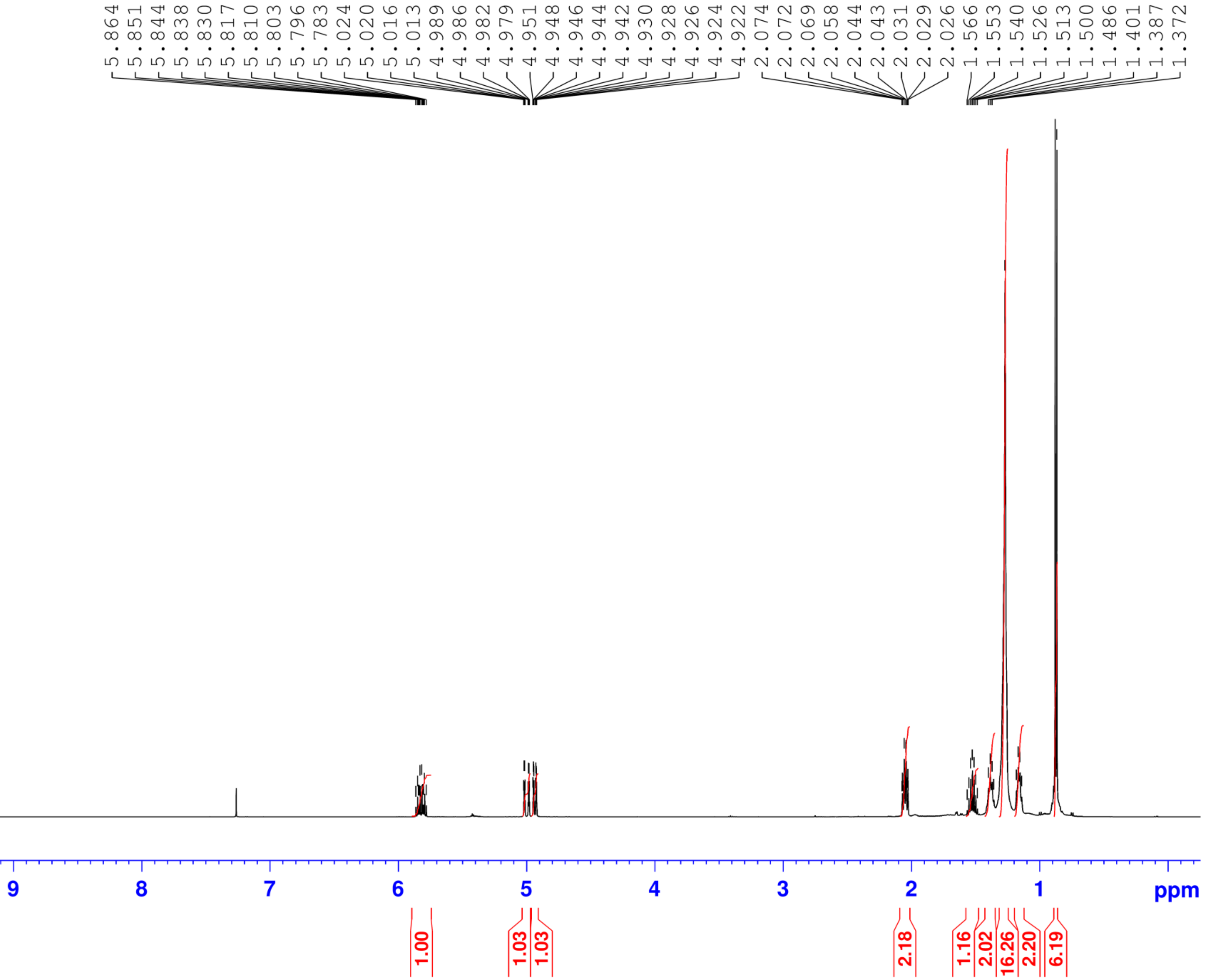

^{13}C NMR (125 MHz, $CDCl_3$) – 14-Methylpentadec-1-ene (**22**)

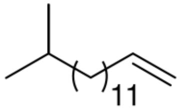

$CDCl_3$

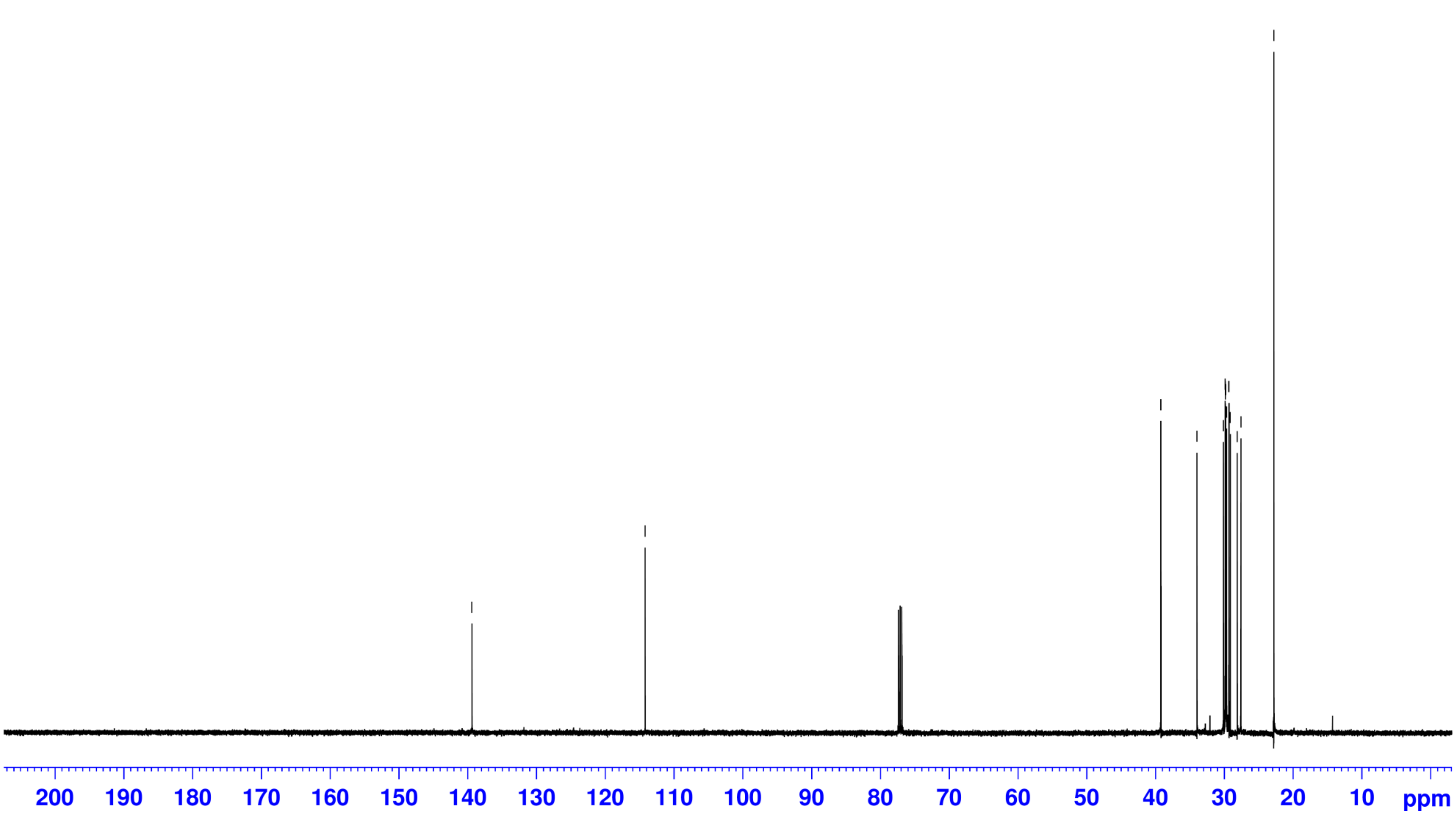

^{1}H NMR (500 MHz, DMSO-d_6) – 16-Methylheptadecane-1,16-diol (**24**)

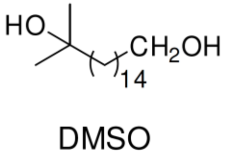

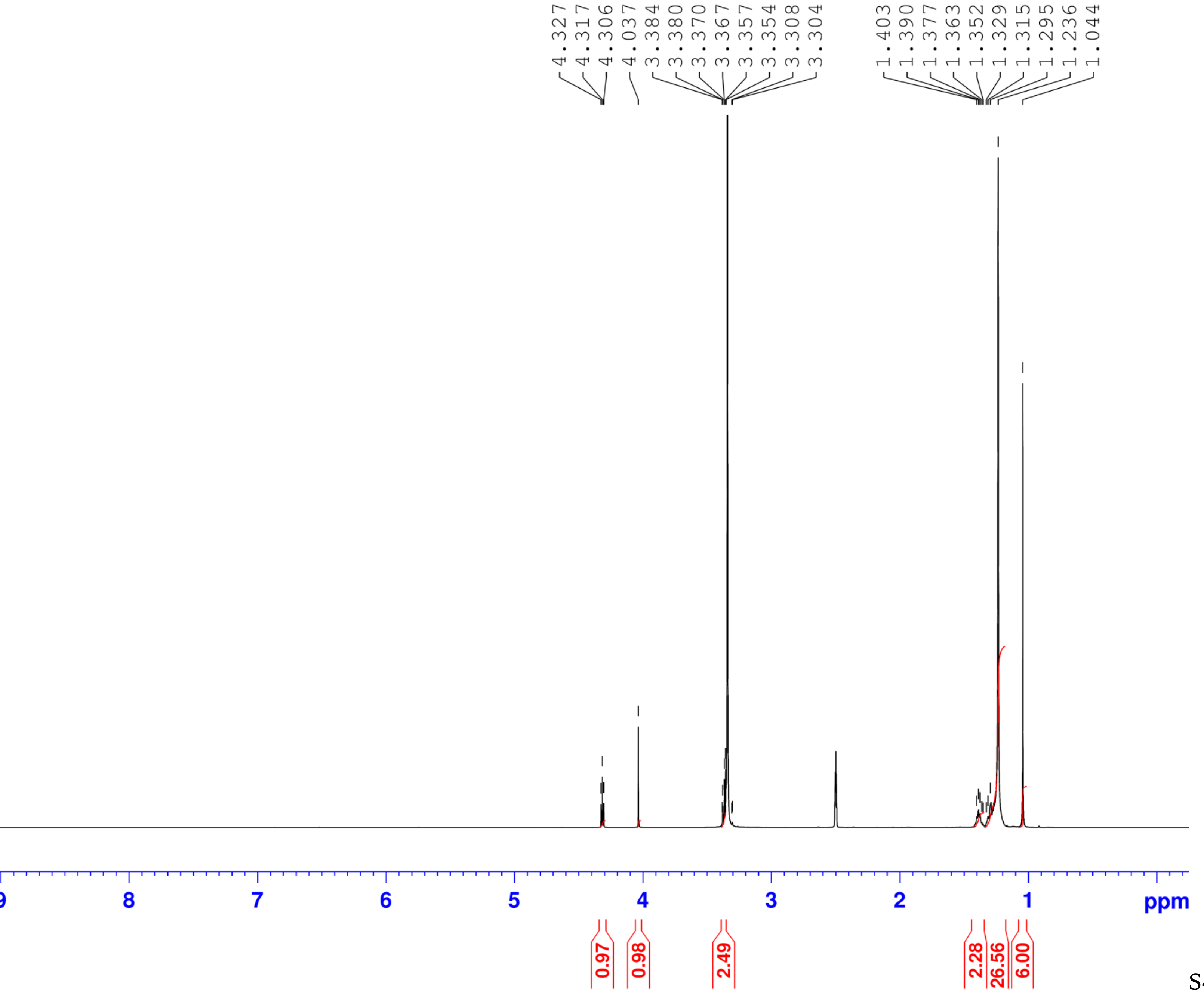

^{13}C NMR (125 MHz, $CDCl_3$) – 16-Methylheptadecane-1,16-diol (**24**)

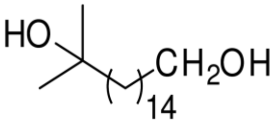

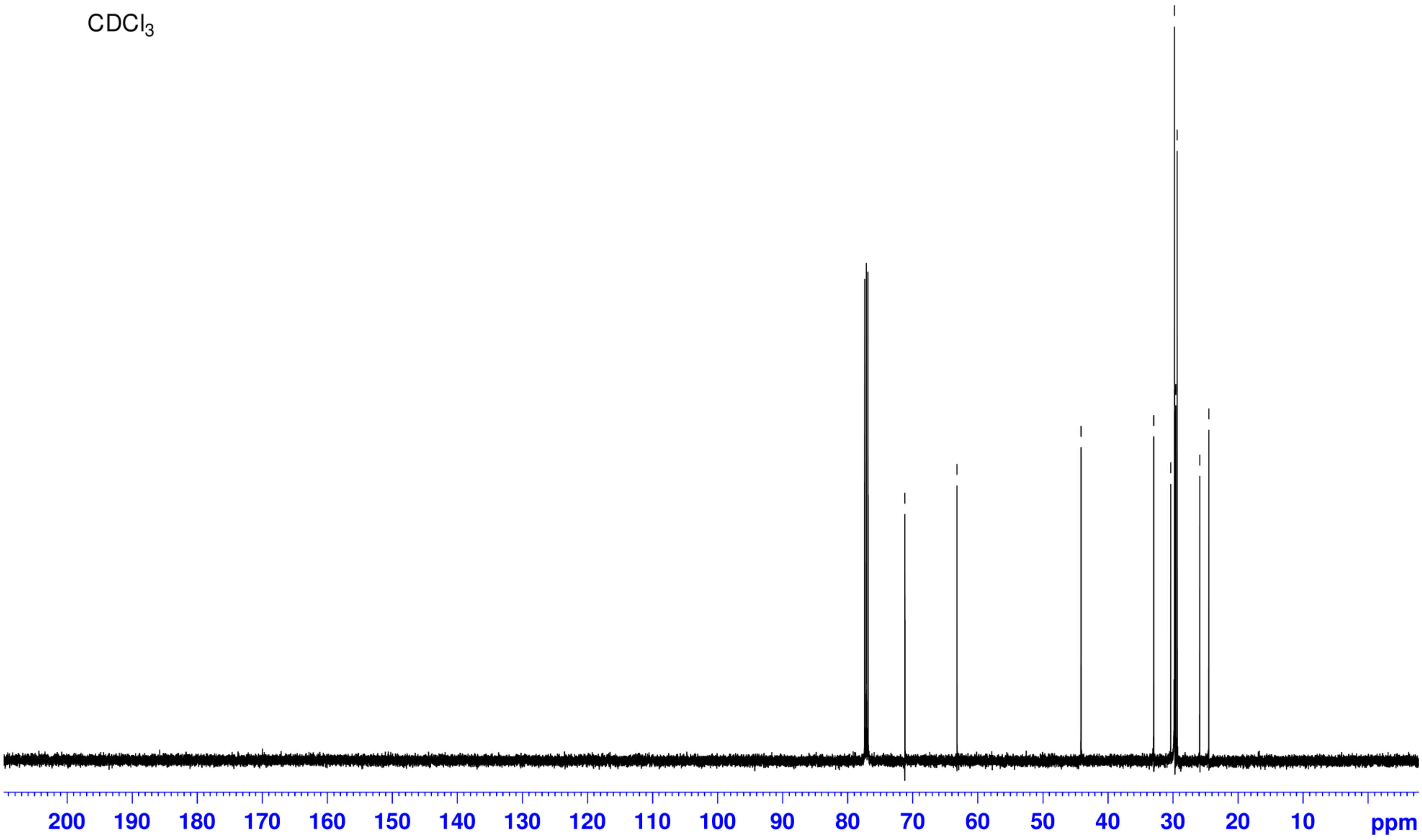

^{1}H NMR (500 MHz, $CDCl_3$) – 16-Methylheptadecan-1-ol (**25**)

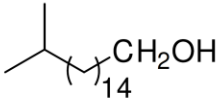

$CDCl_3$

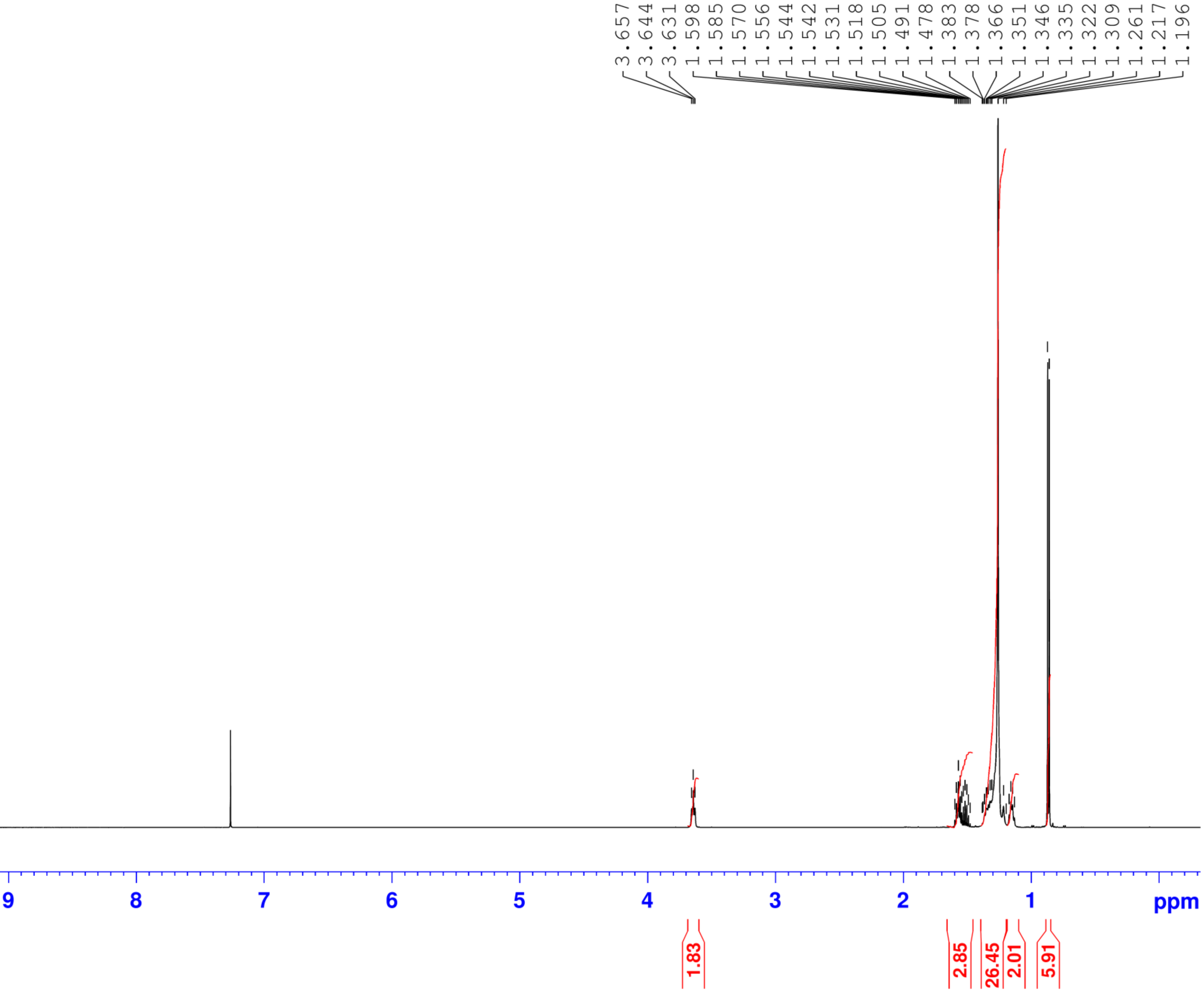

^{13}C NMR (125 MHz, $CDCl_3$) – 16-Methylheptadecan-1-ol (**25**)

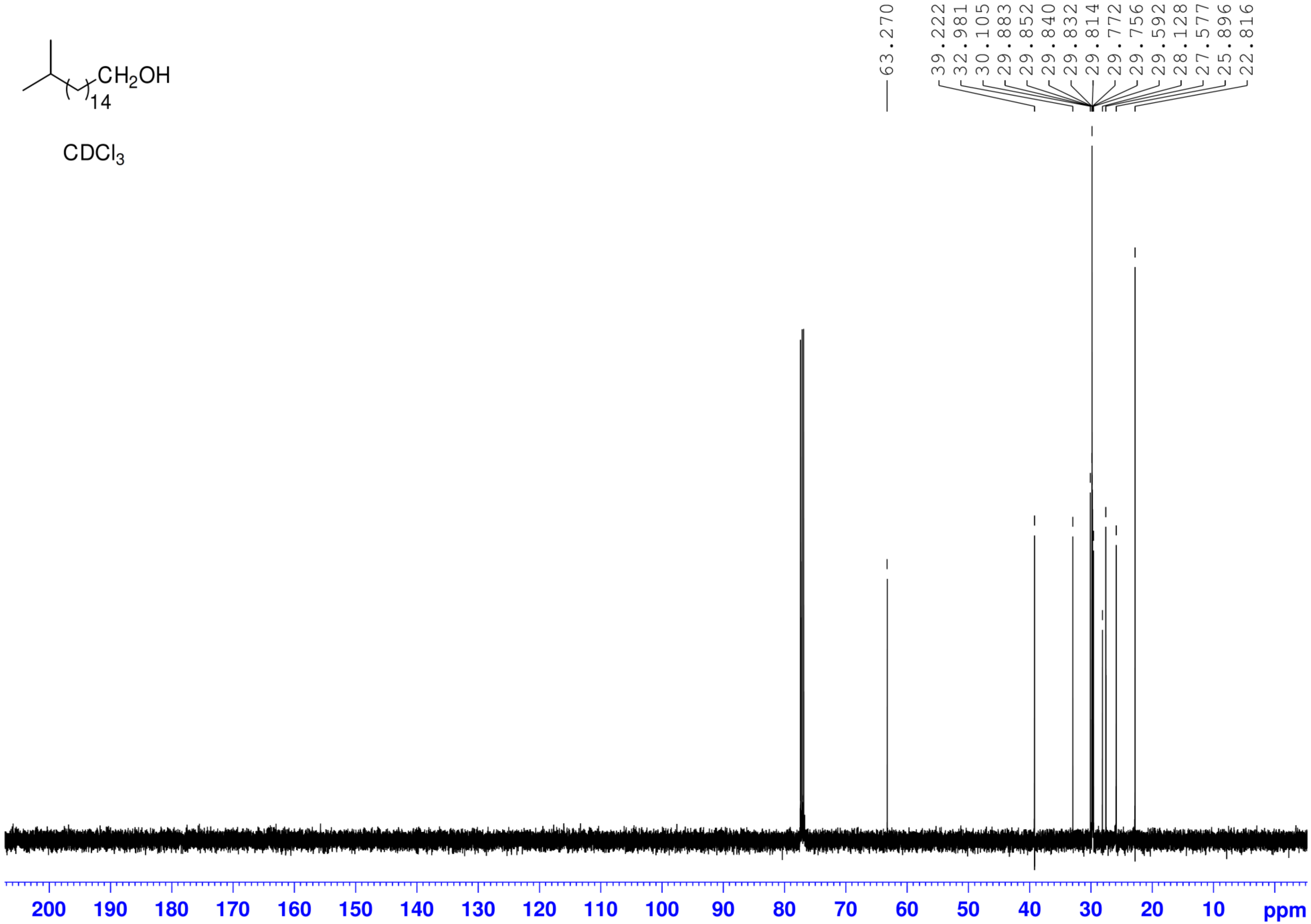

^{1}H NMR (500 MHz, $CDCl_3$) – 16-Methylheptadecyl methanesulfonate (**26**)

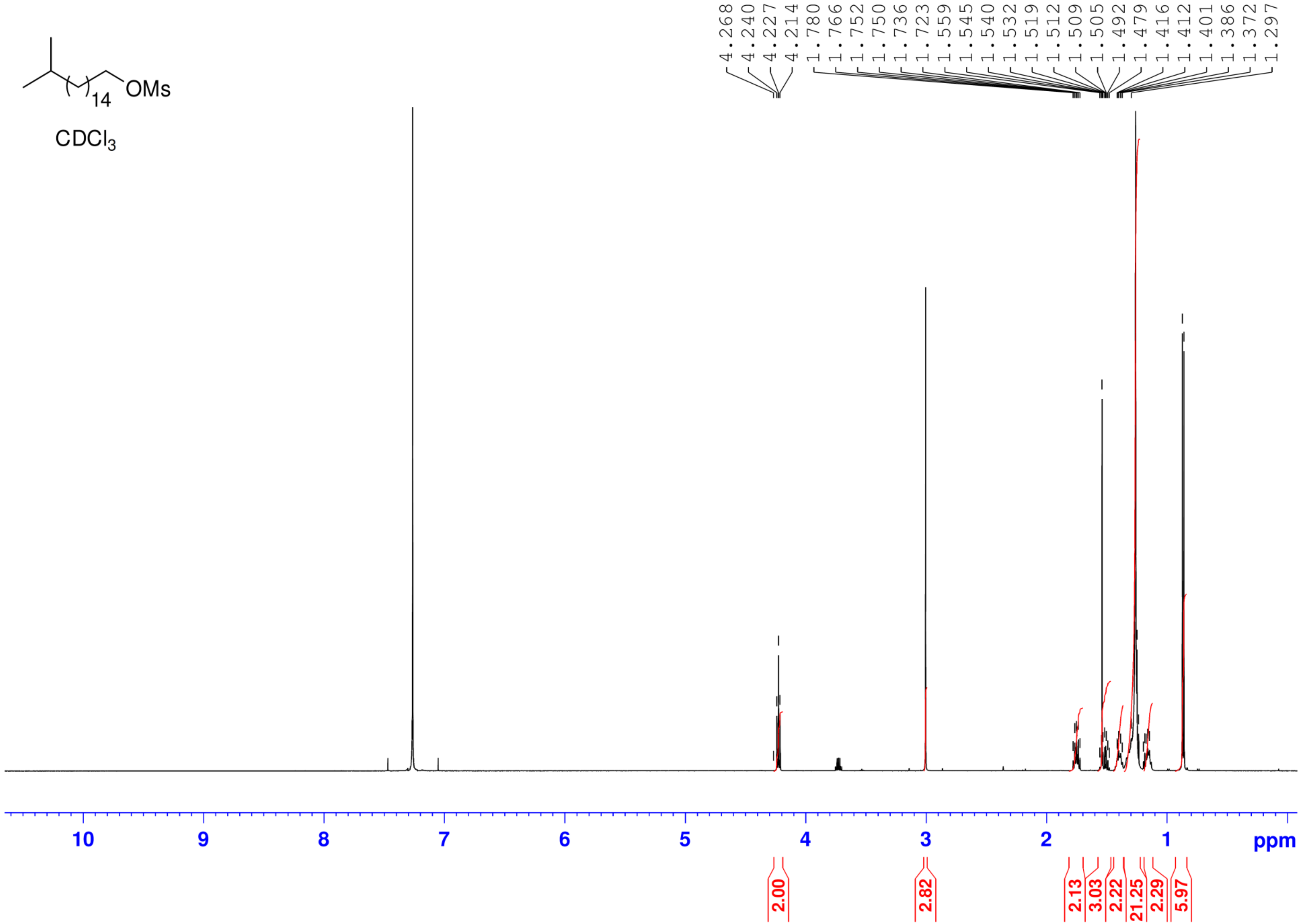

^{13}C NMR (125 MHz, $CDCl_3$) – 16-Methylheptadecyl methanesulfonate (**26**)

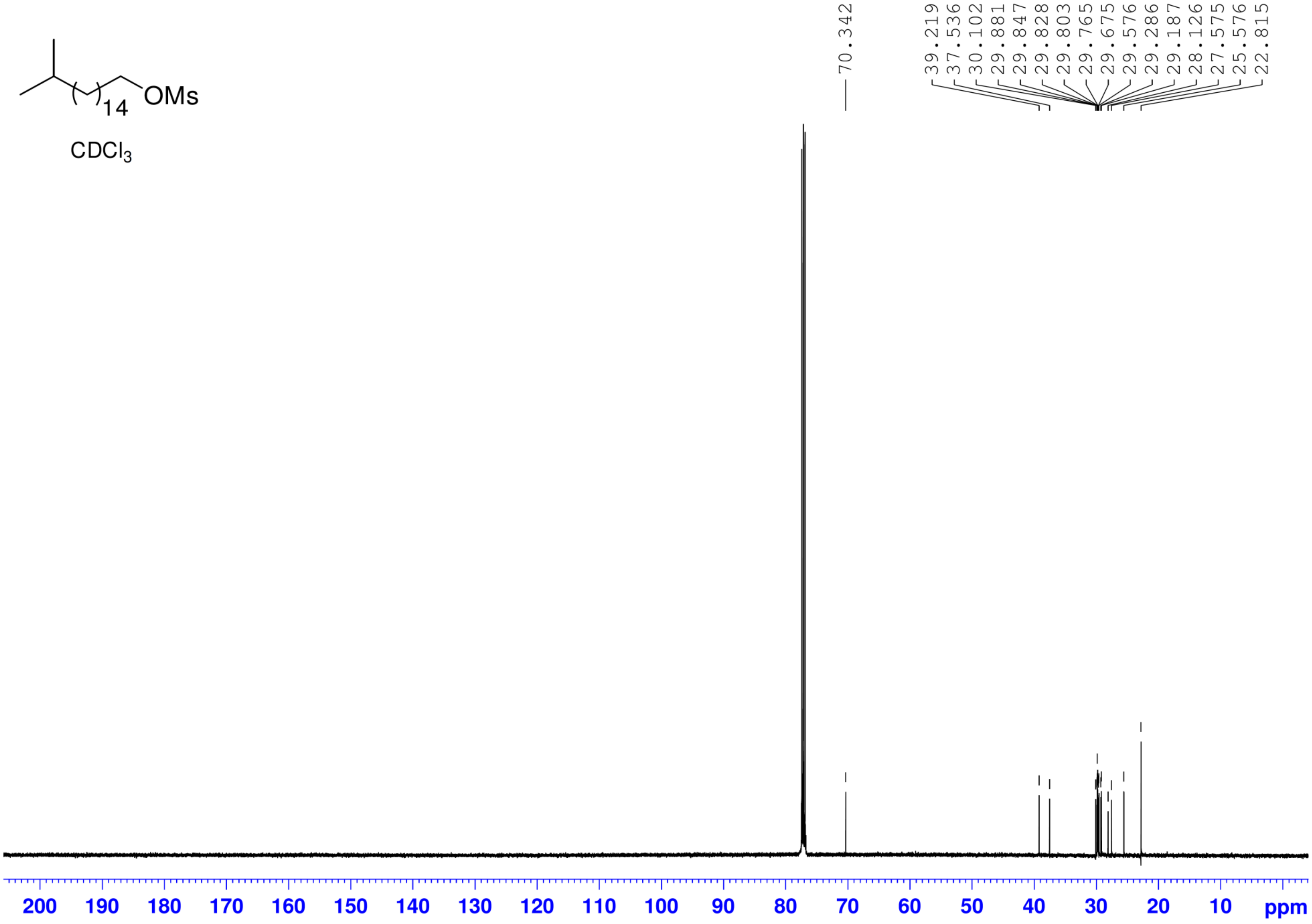

^{1}H NMR (500 MHz, $CDCl_3$) – 17-Methyloctadecanenitrile (**27**)

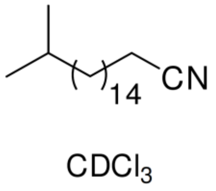

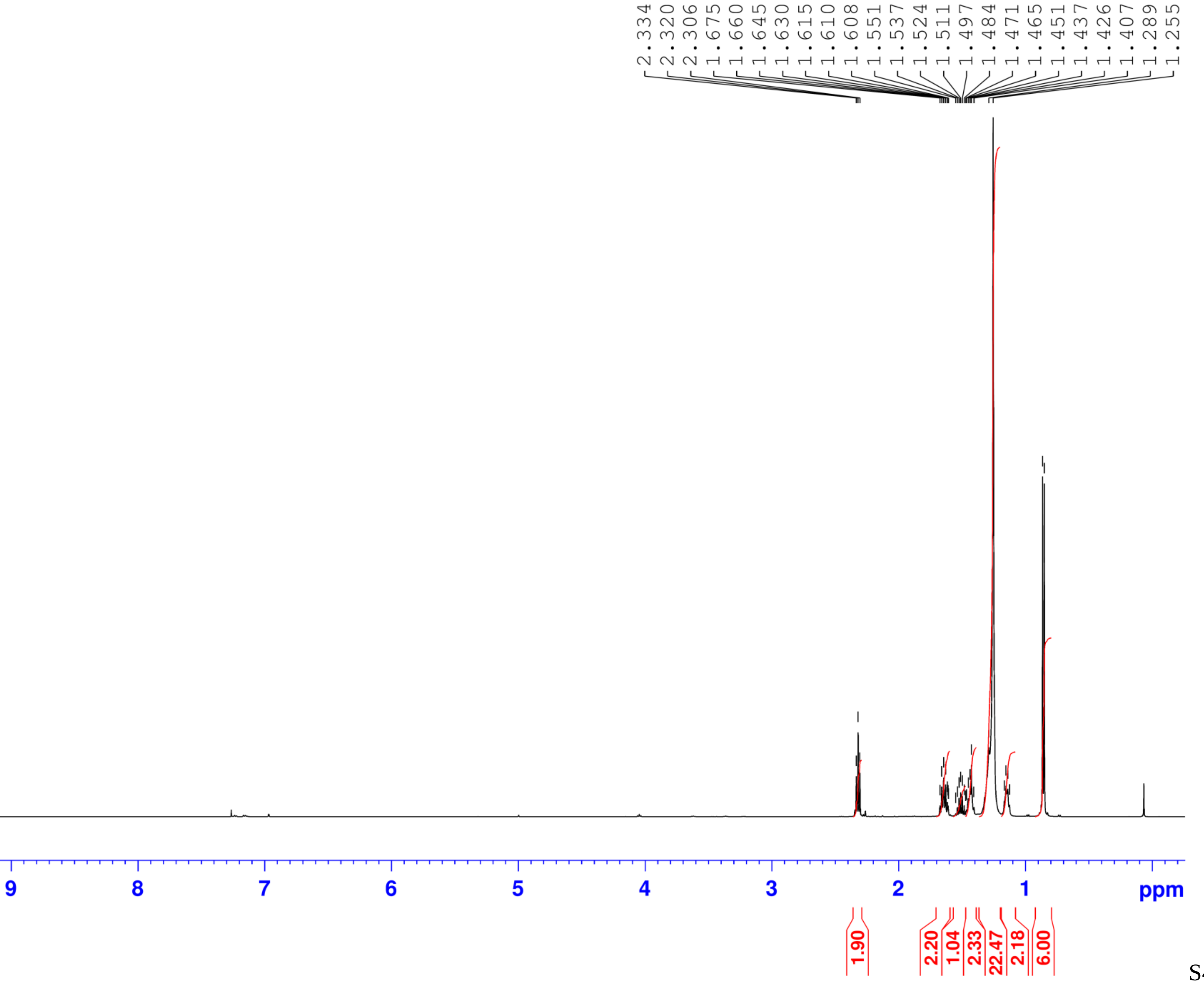

^{13}C NMR (125 MHz, $CDCl_3$) – 17-Methyloctadecanenitrile (**27**)

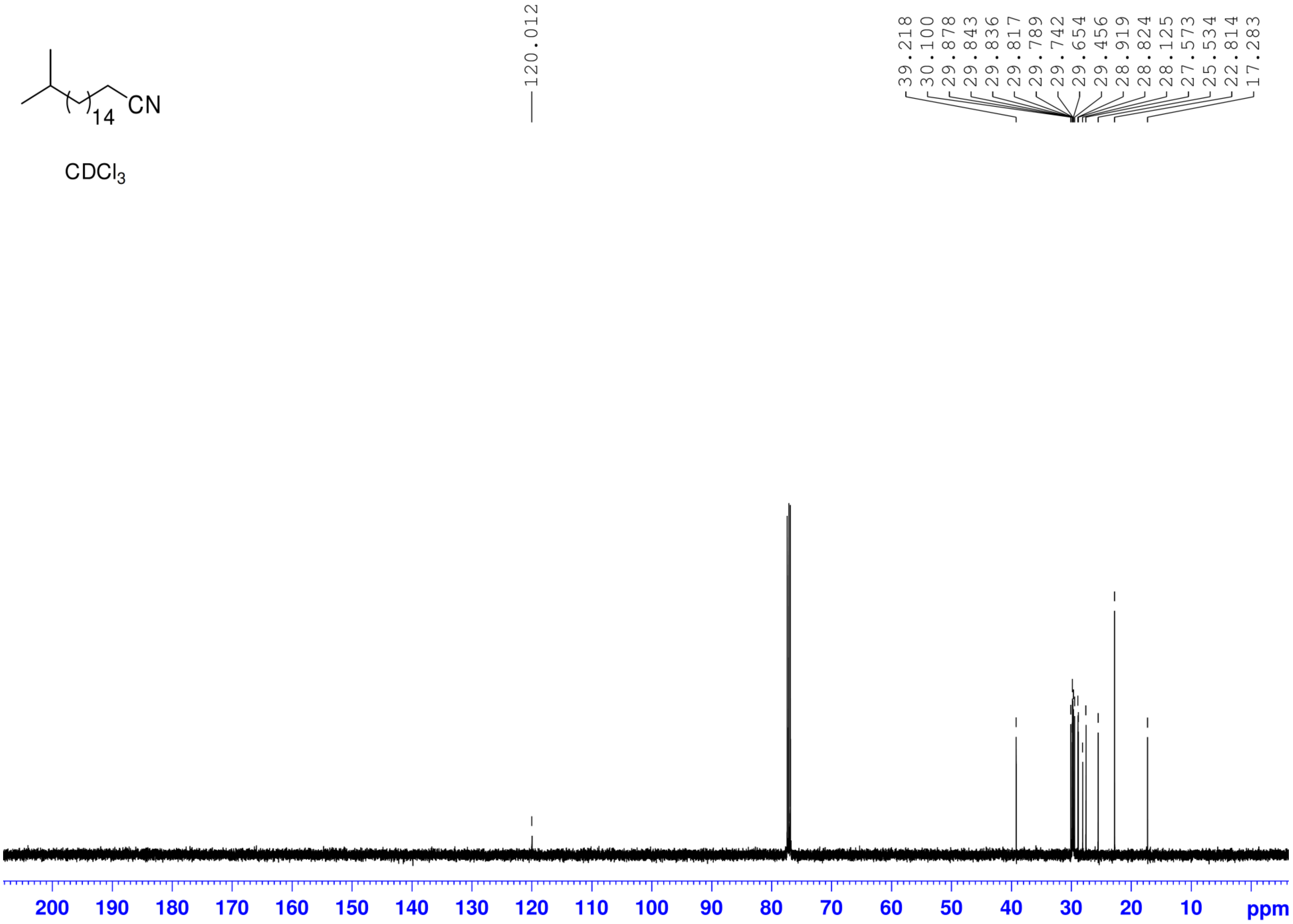

^{1}H NMR (500 MHz, $CDCl_3$) – (*S*)-4-Benzyl-3-(15-methylhexadecanoyl)oxazolidin-2-one (**28**)

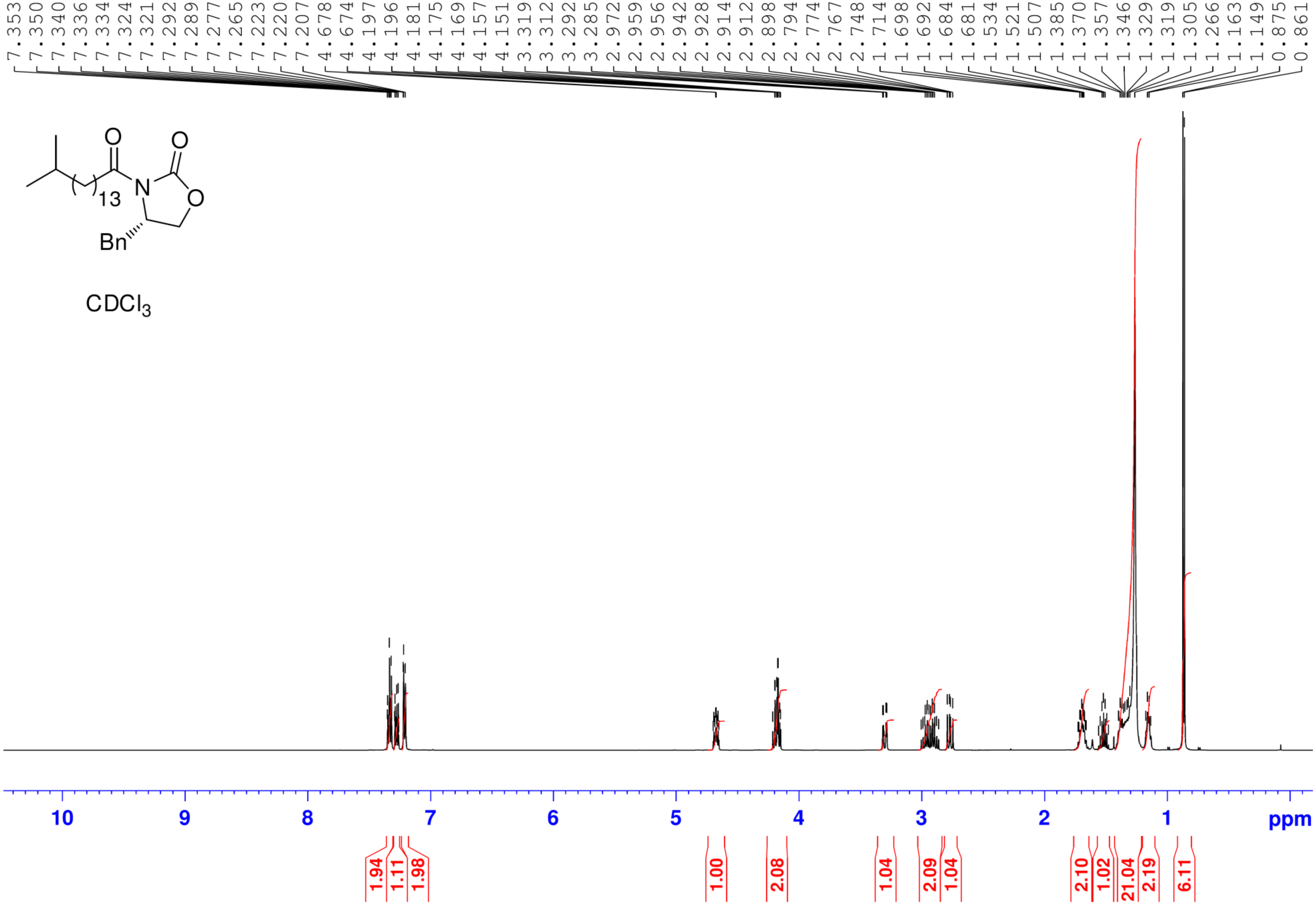

^{13}C NMR (125 MHz, $CDCl_3$) – (*S*)-4-Benzyl-3-(15-methylhexadecanoyl)oxazolidin-2-one (**28**)

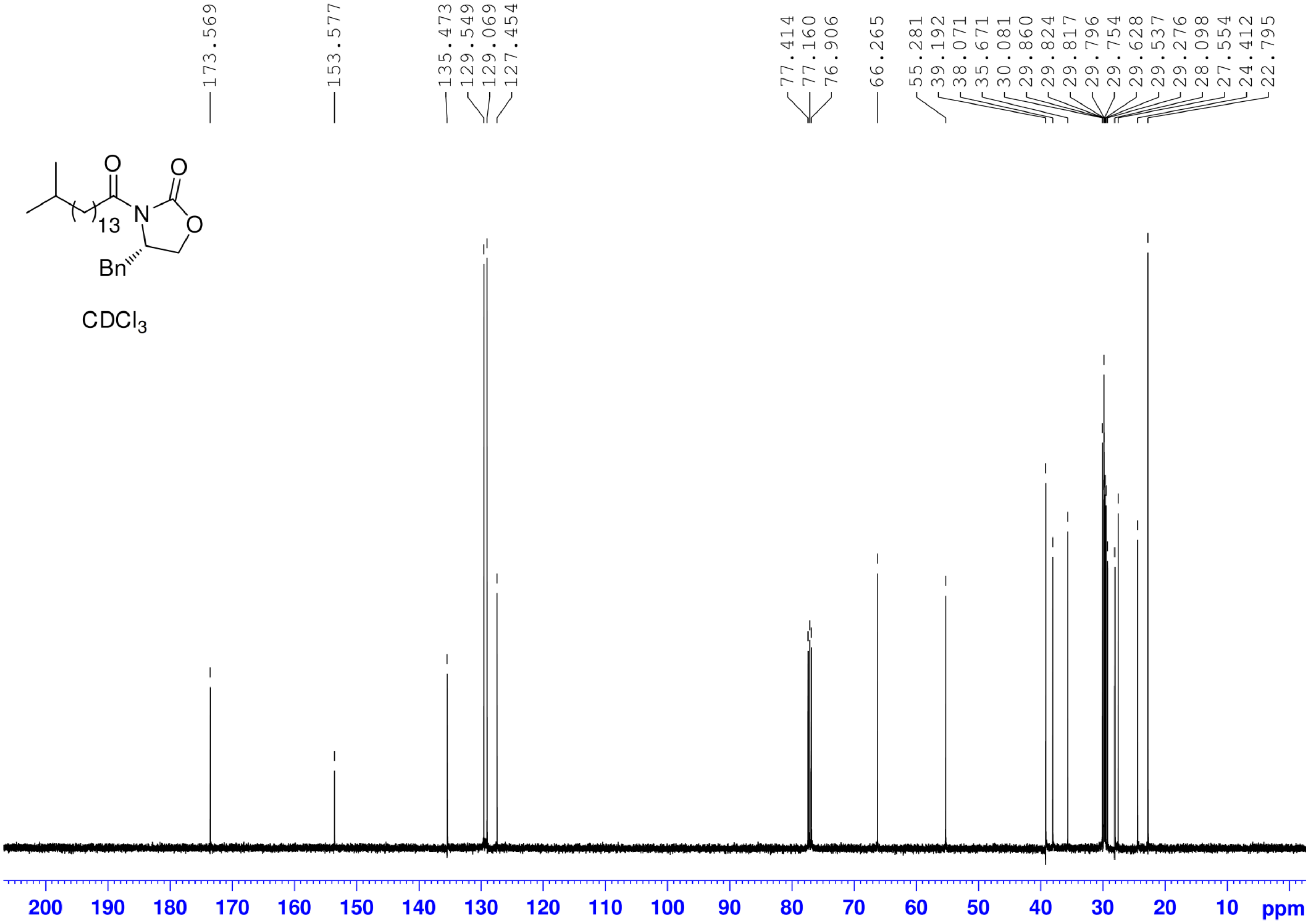

^{13}C NMR (125 MHz, $CDCl_3$) – (*S*)-4-Benzyl-3-((*S*)-2,15-dimethylhexadecanoyl)oxazolidin-2-one (**29**)

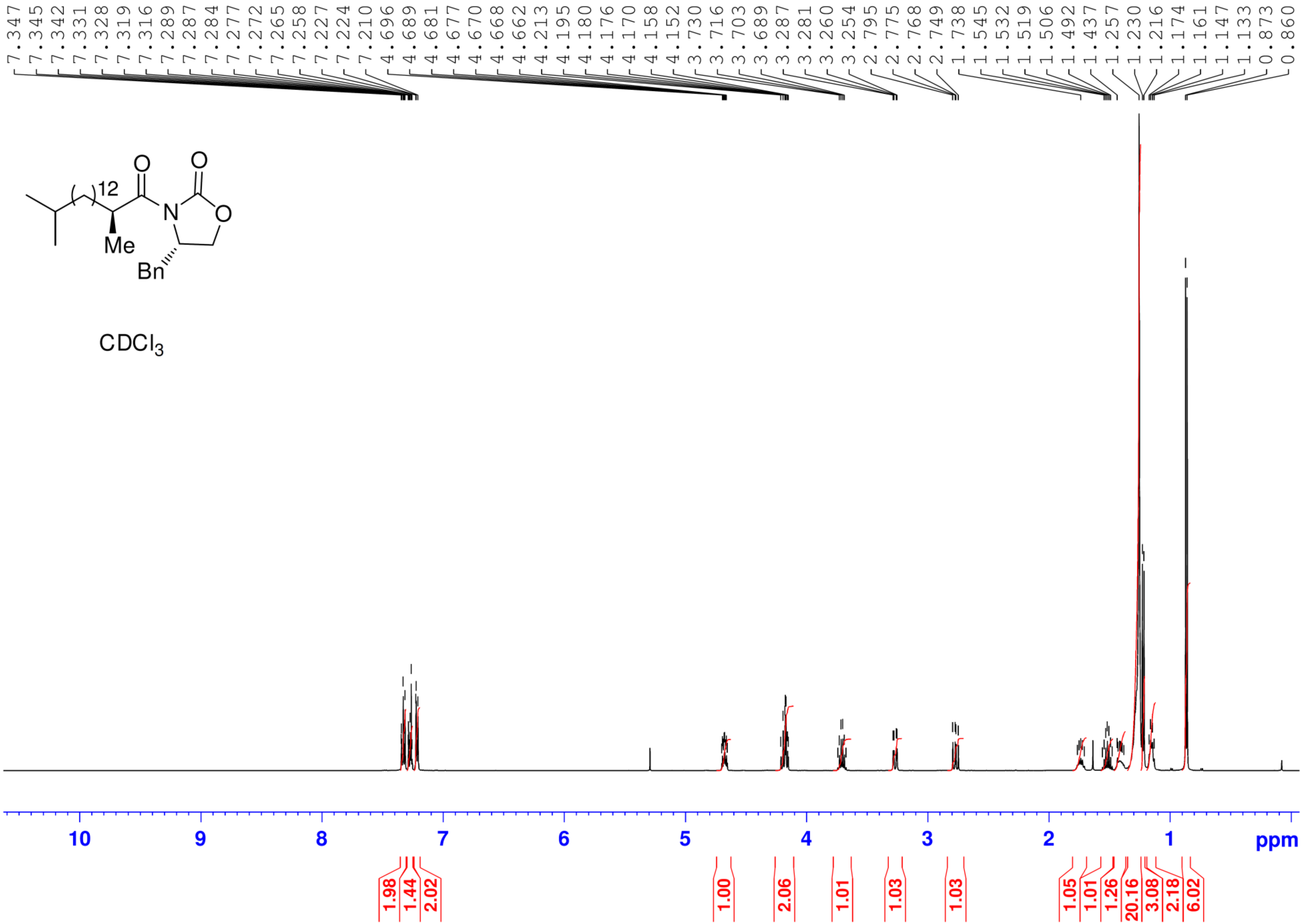

^{1}H NMR (500 MHz, $CDCl_3$) – (*S*)-4-Benzyl-3-((*S*)-2,15-dimethylhexadecanoyl)oxazolidin-2-one (**29**)

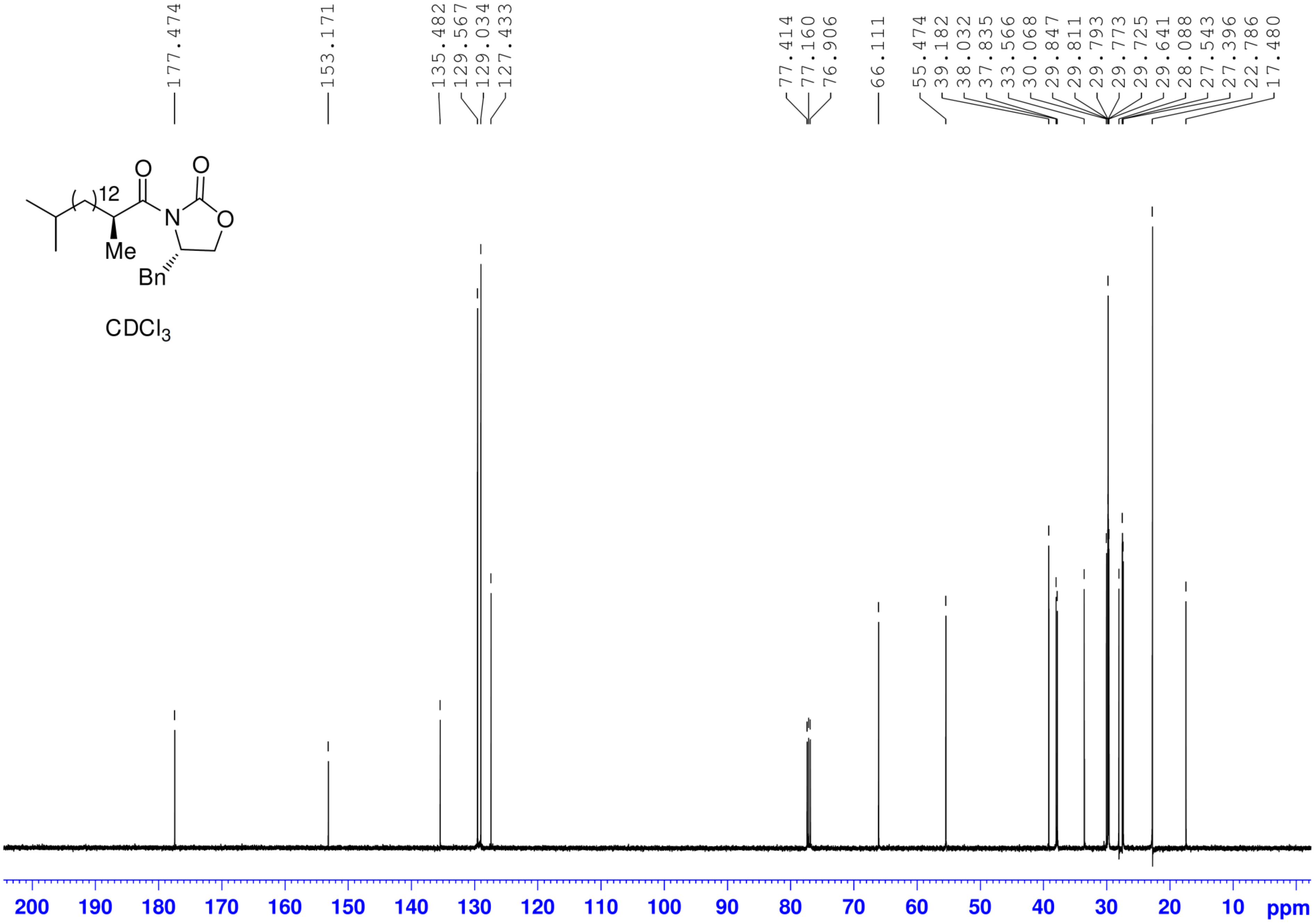

^{1}H NMR (500 MHz, $CDCl_3$) – (*S*)-2,15-Dimethylhexadecanoic acid (**30**)

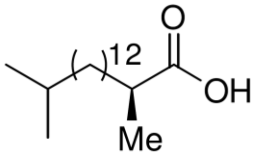

$CDCl_3$

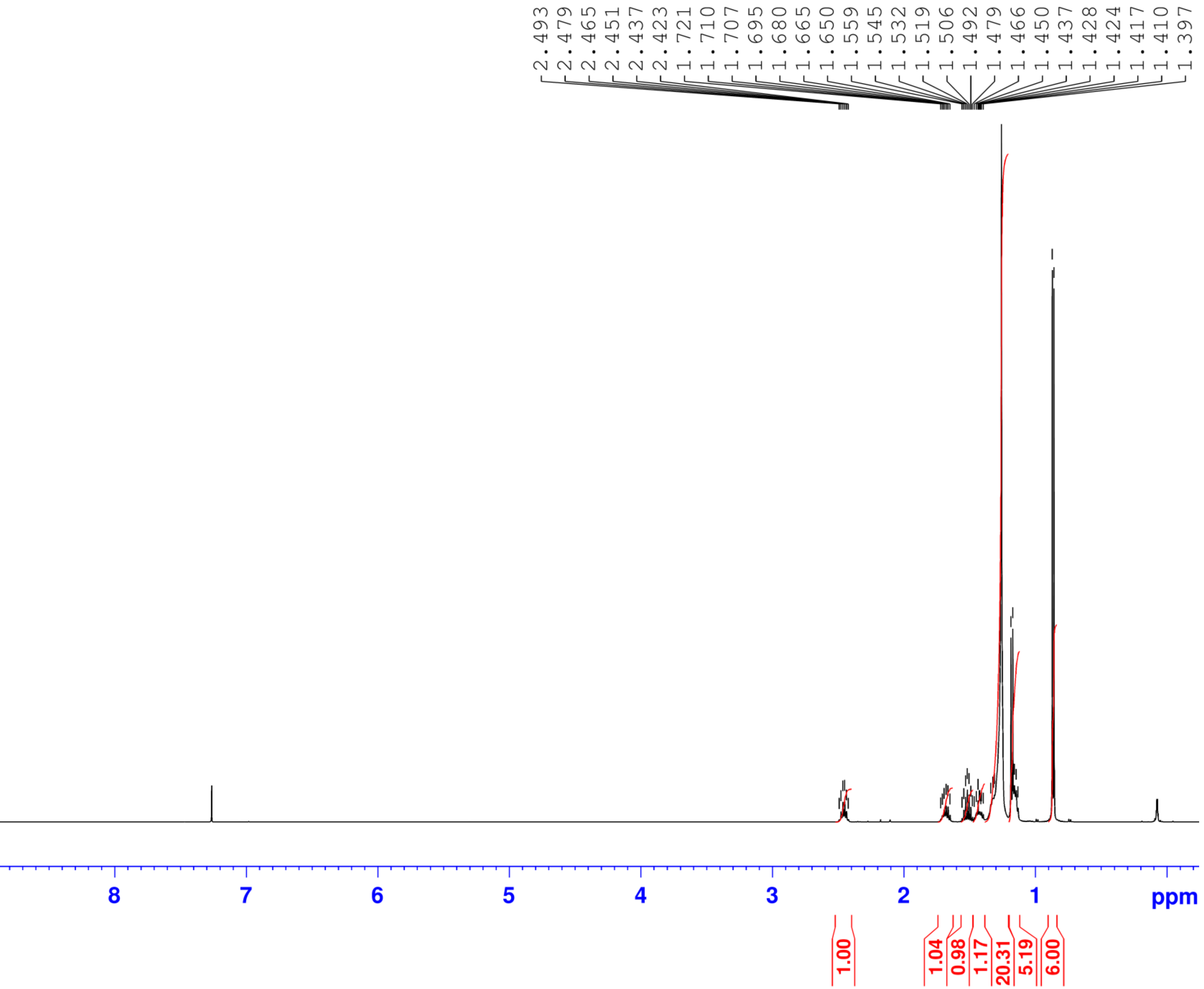

^{13}C NMR (125 MHz, $CDCl_3$) – (*S*)-2,15-Dimethylhexadecanoic acid (**30**)

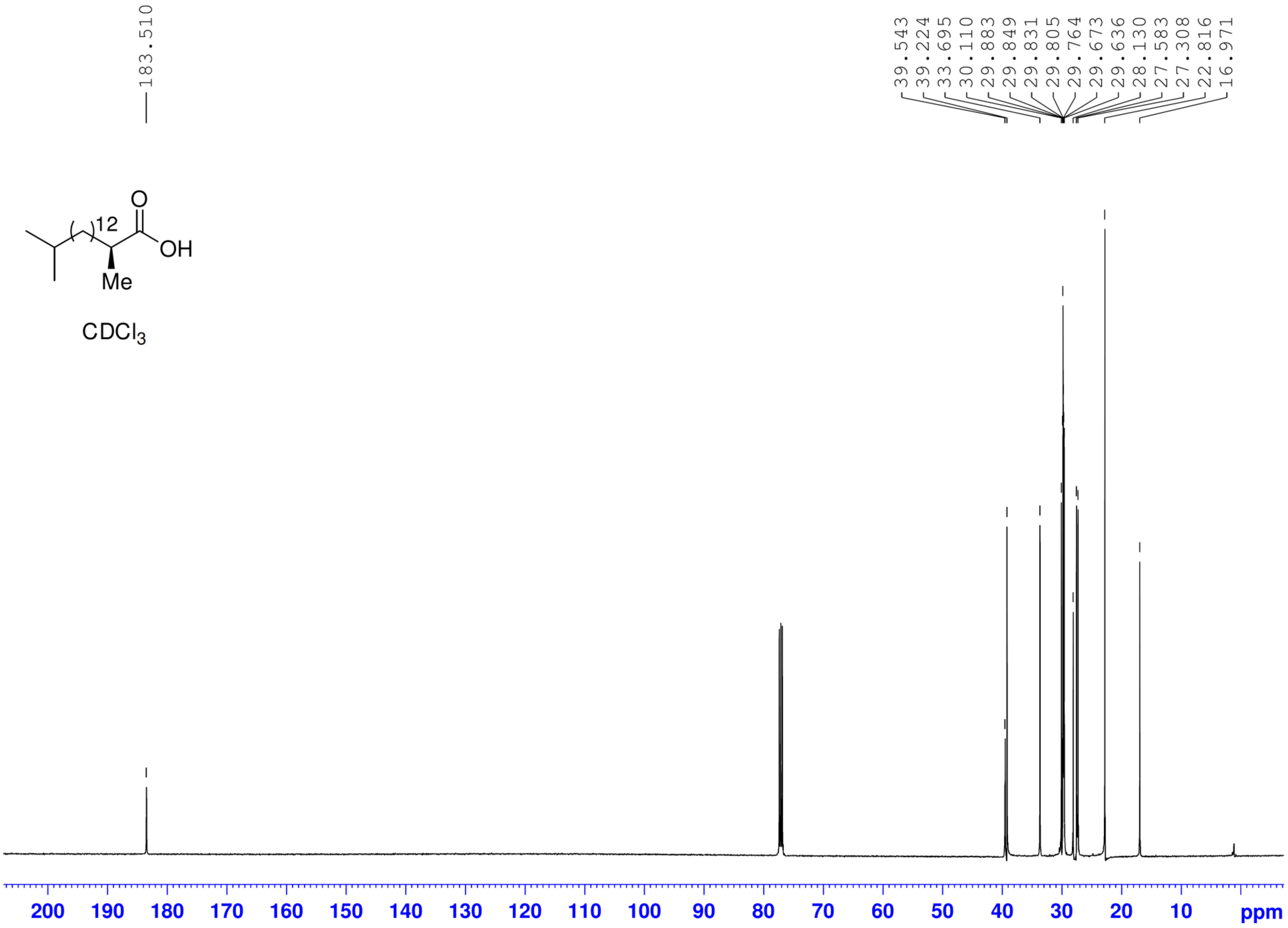

^{1}H NMR (500 MHz, $CDCl_3$) – 3-Phenyl-2-(phenylsulfonyl)-1,2-oxaziridine

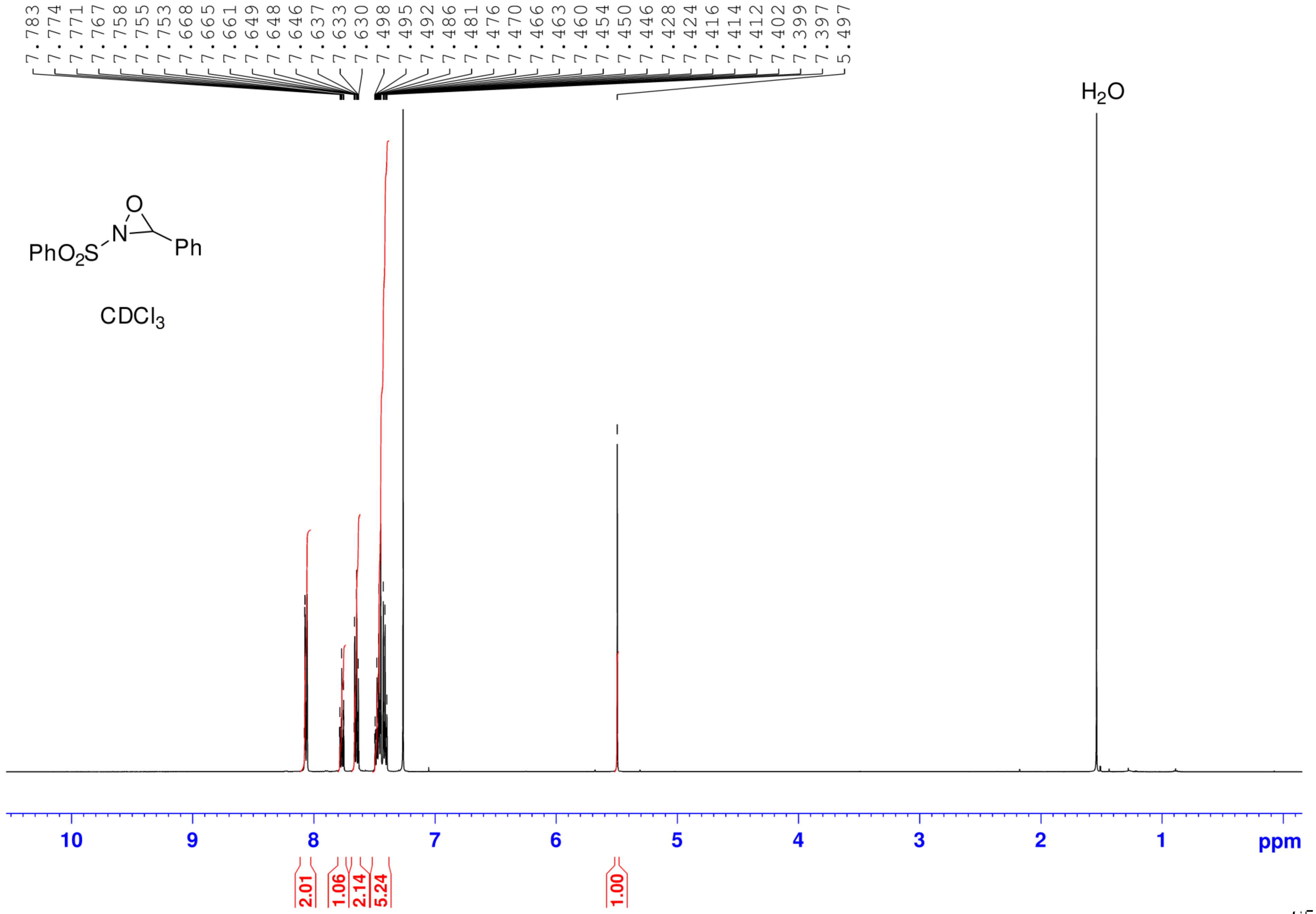

^{13}C NMR (125 MHz, $CDCl_3$) – 3-Phenyl-2-(phenylsulfonyl)-1,2-oxaziridine

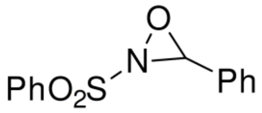

CDCl3

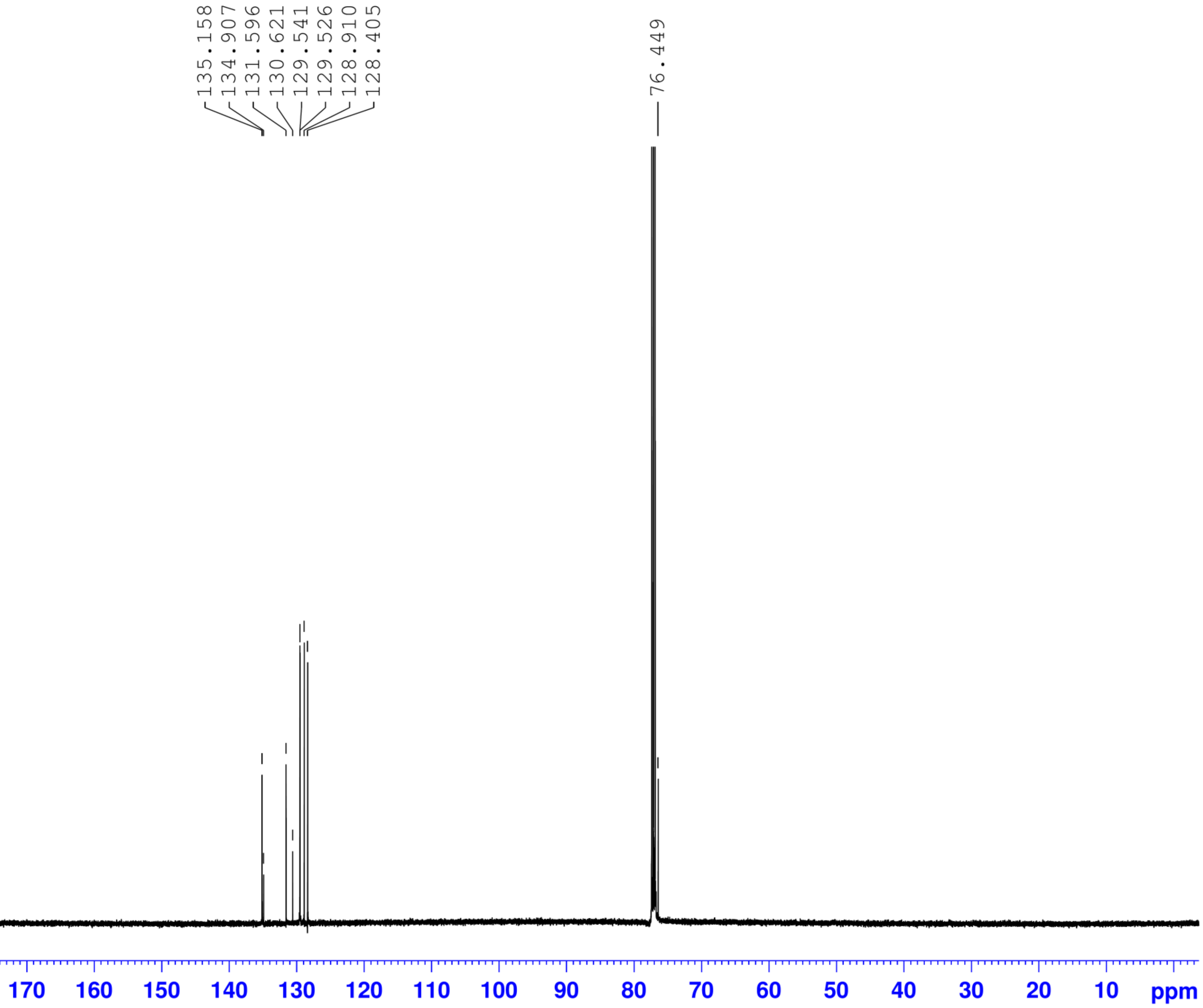

^{1}H NMR (500 MHz, $CDCl_3$) – (*S*)-4-Benzyl-3-((*S*)-2-hydroxy-15-methylhexadecanoyl)oxazolidin-2-one (**31**)

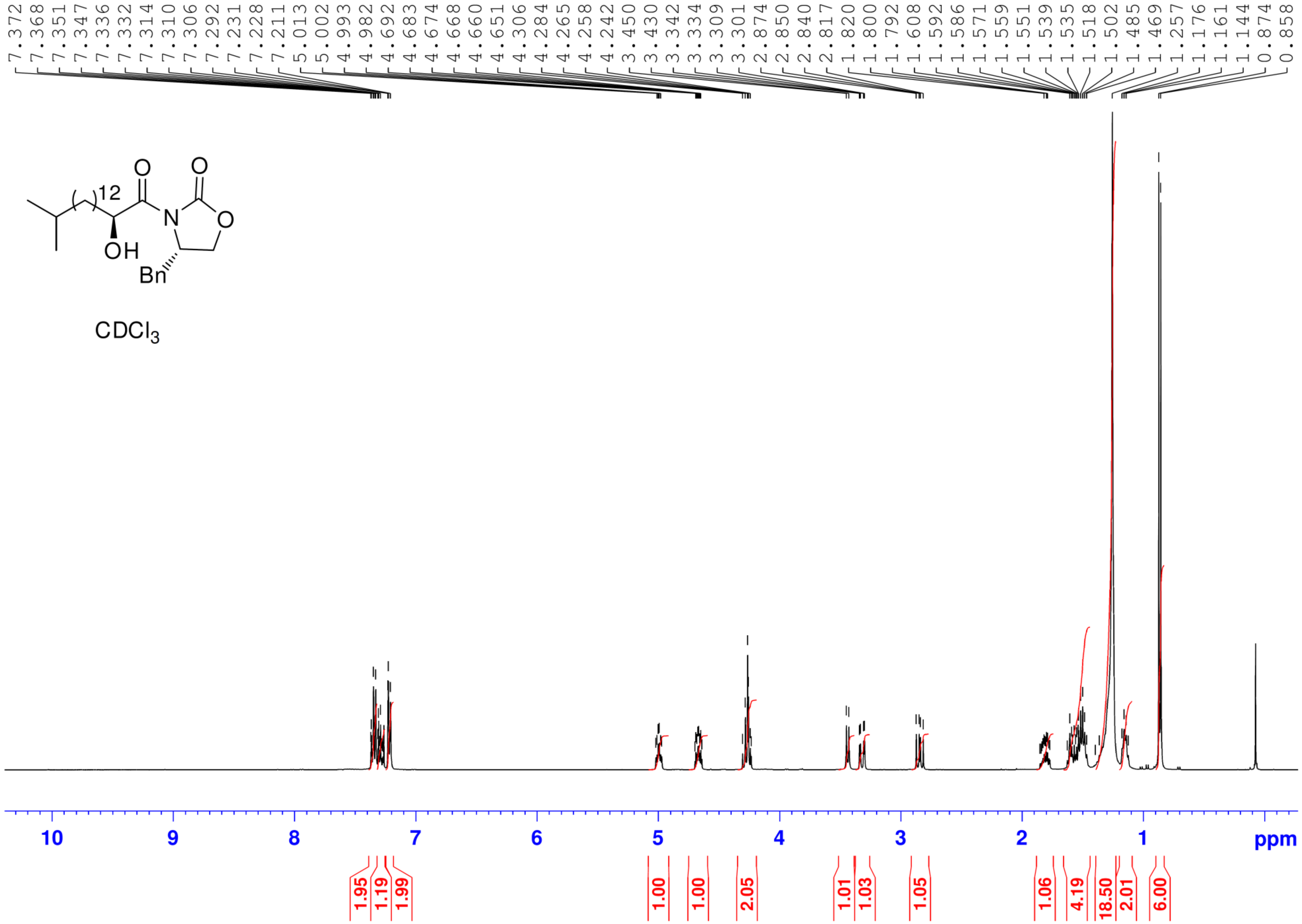

^{13}C NMR (125 MHz, $CDCl_3$) – (*S*)-4-Benzyl-3-((*S*)-2-hydroxy-15-methylhexadecanoyl)oxazolidin-2-one (**31**)

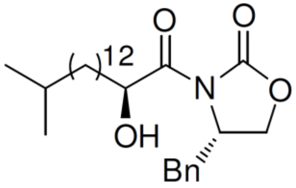

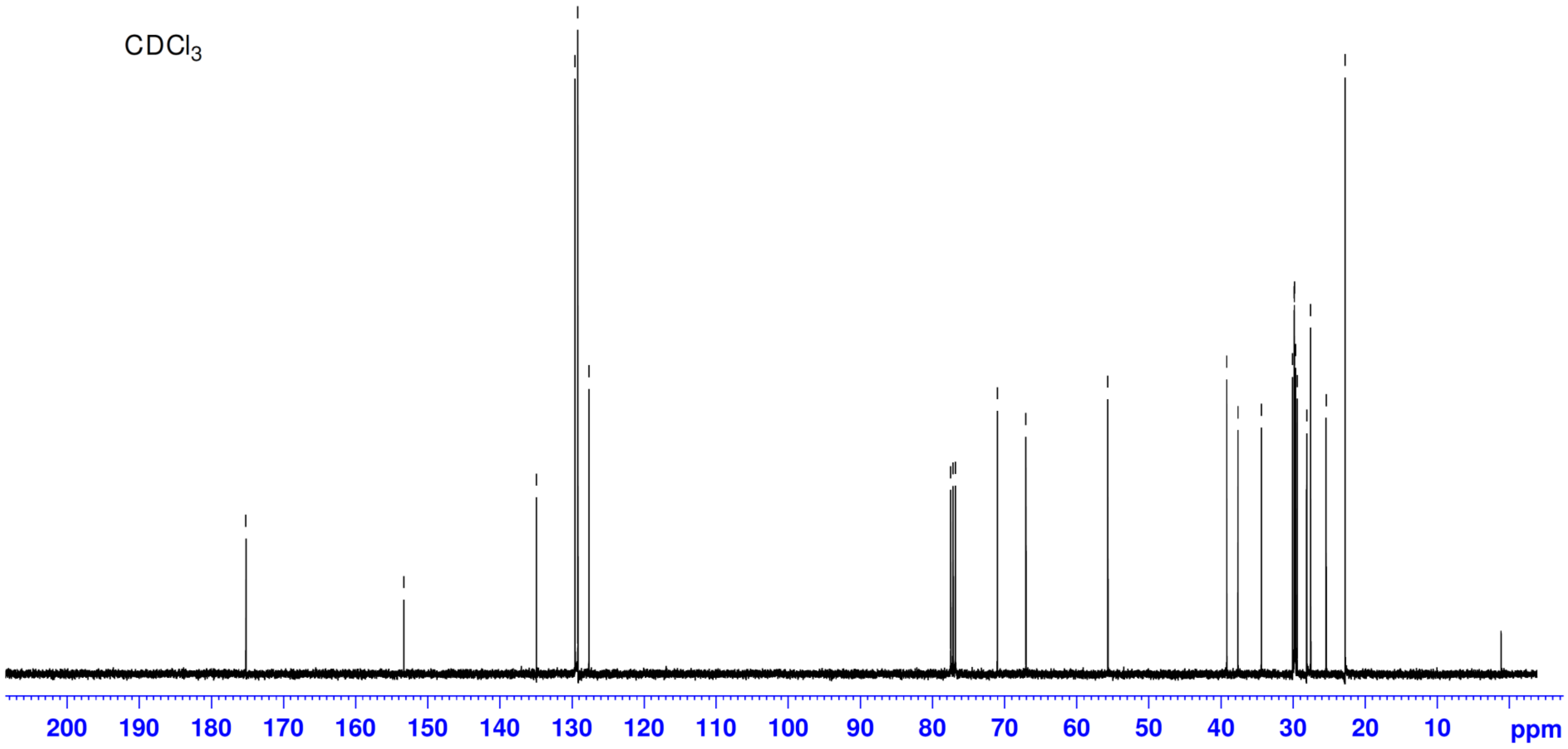

^{1}H NMR (500 MHz, $CDCl_3$) – (*S*)-Methyl 2-hydroxy-15-methylhexadecanoate

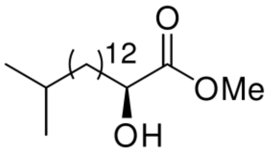

$CDCl_3$

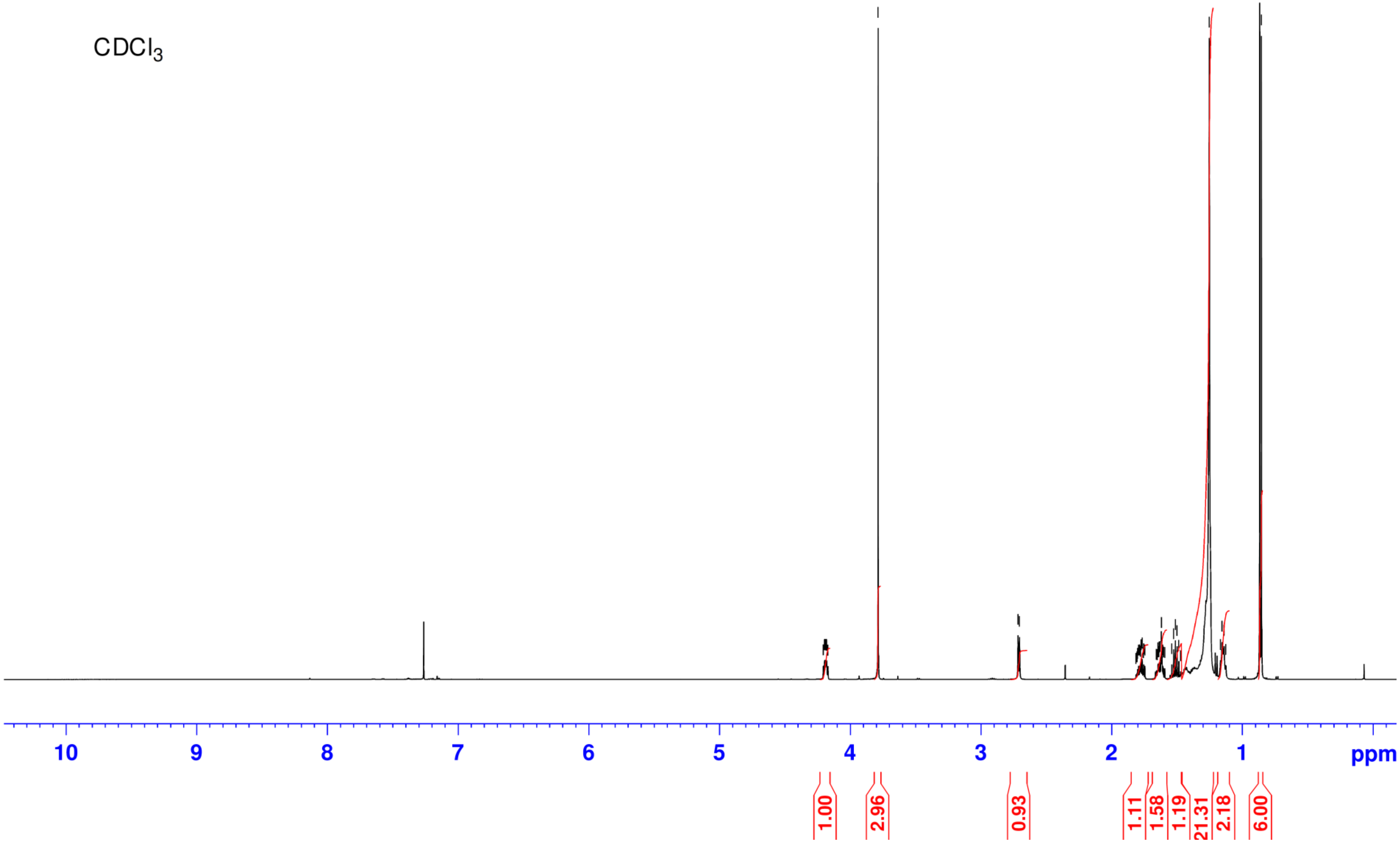

^{13}C NMR (125 MHz, $CDCl_3$) – (*S*)-Methyl 2-hydroxy-15-methylhexadecanoate

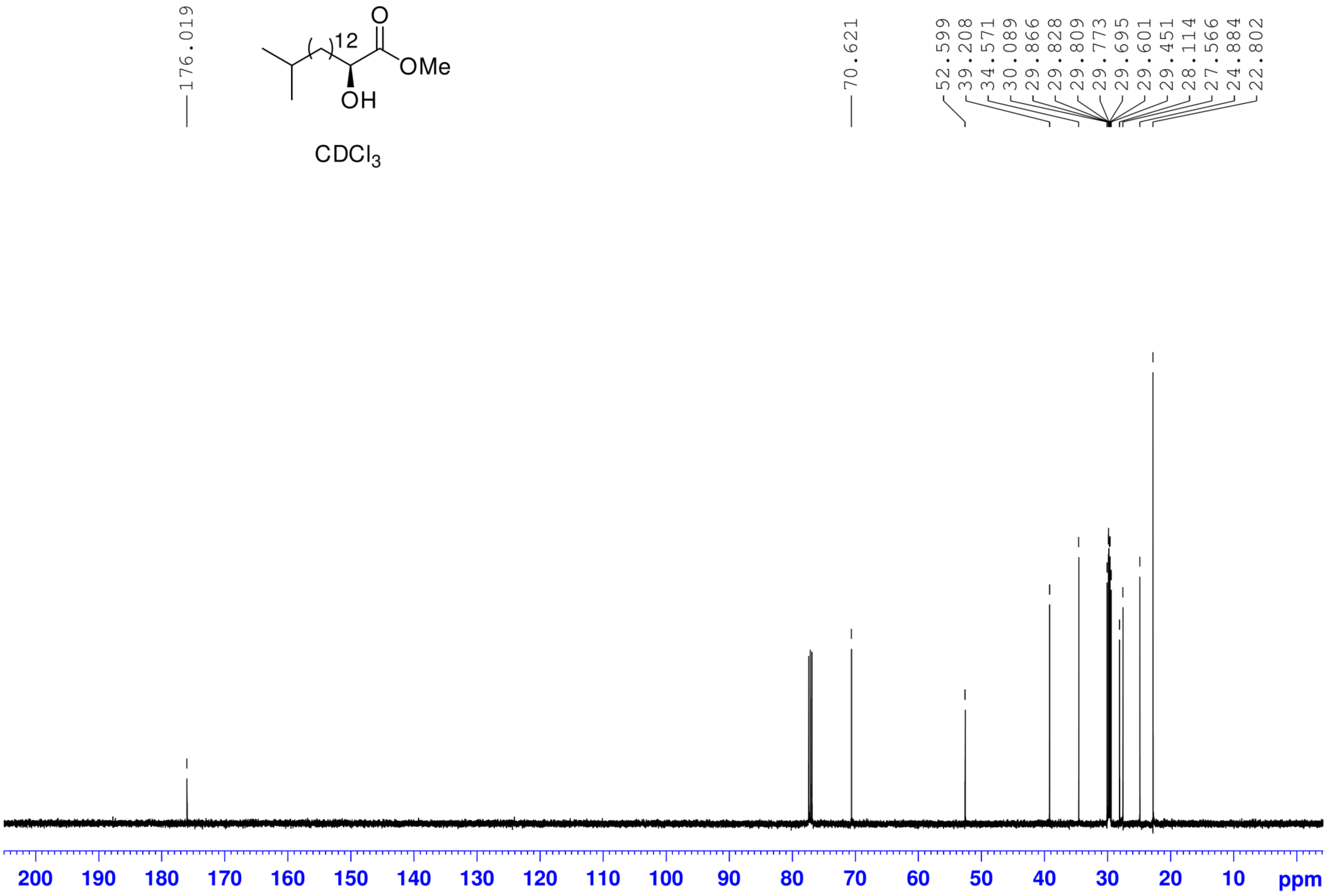

^{1}H NMR (500 MHz, $CDCl_3/CD_3OD$) – (*S*)-2-Hydroxy-15-methylhexadecanoic acid (**32**)

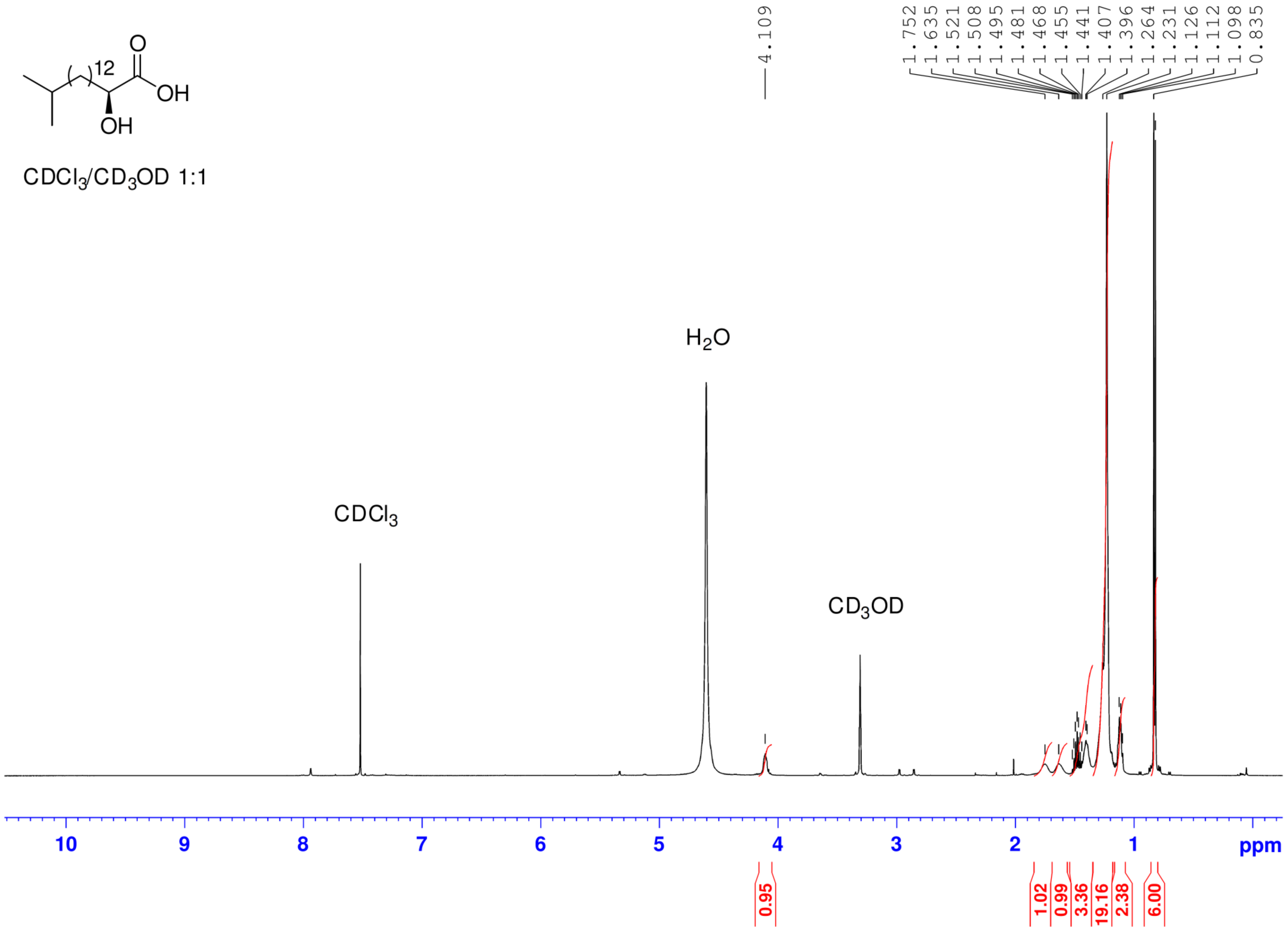

^{13}C NMR (125 MHz, DMSO-d_6/TFA) – (*S*)-2-Hydroxy-15-methylhexadecanoic acid (**32**)

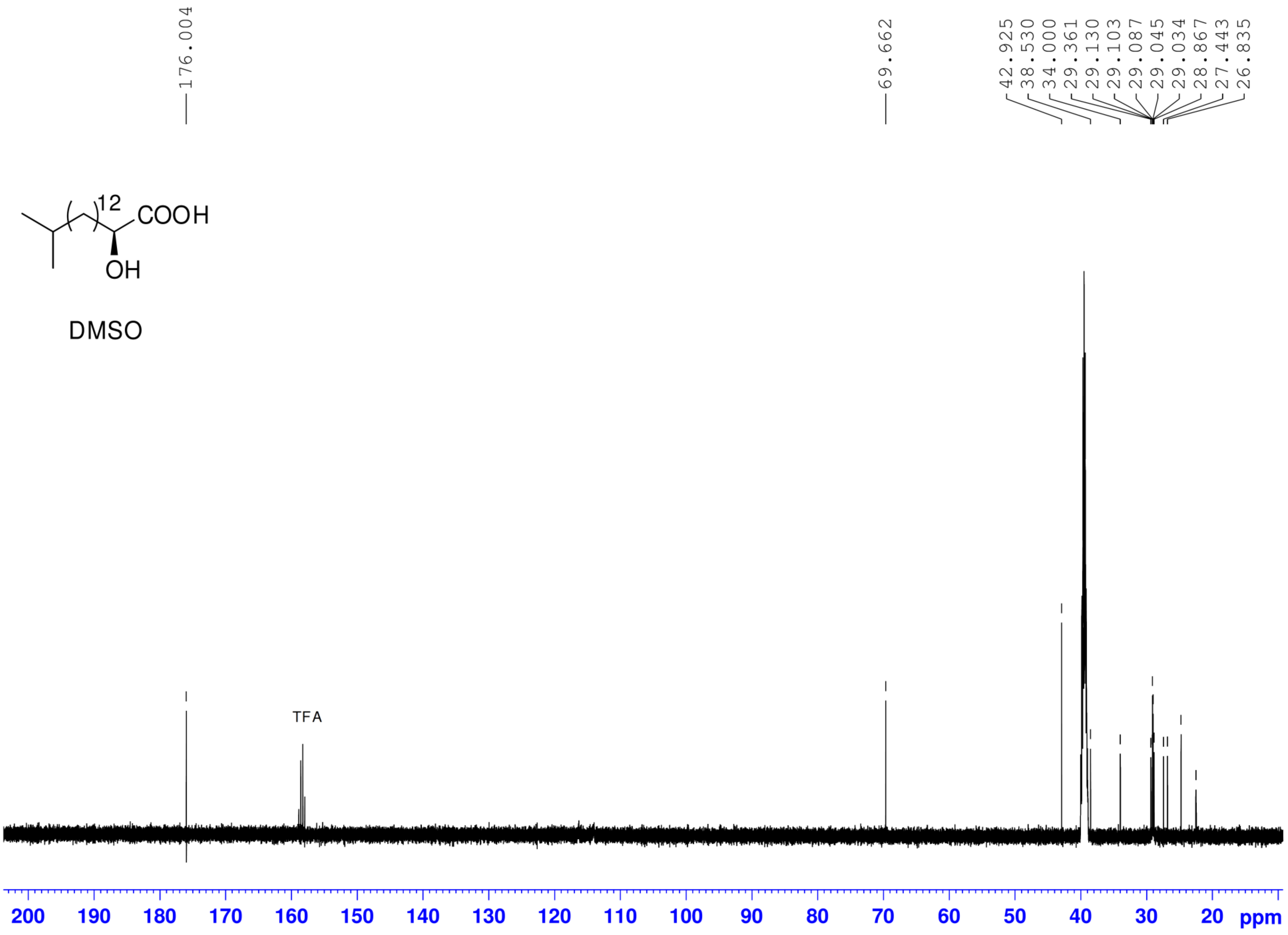

^{1}H NMR (500 MHz, $CDCl_3$) – 14-Methylpentadecan-2-one (**33**)

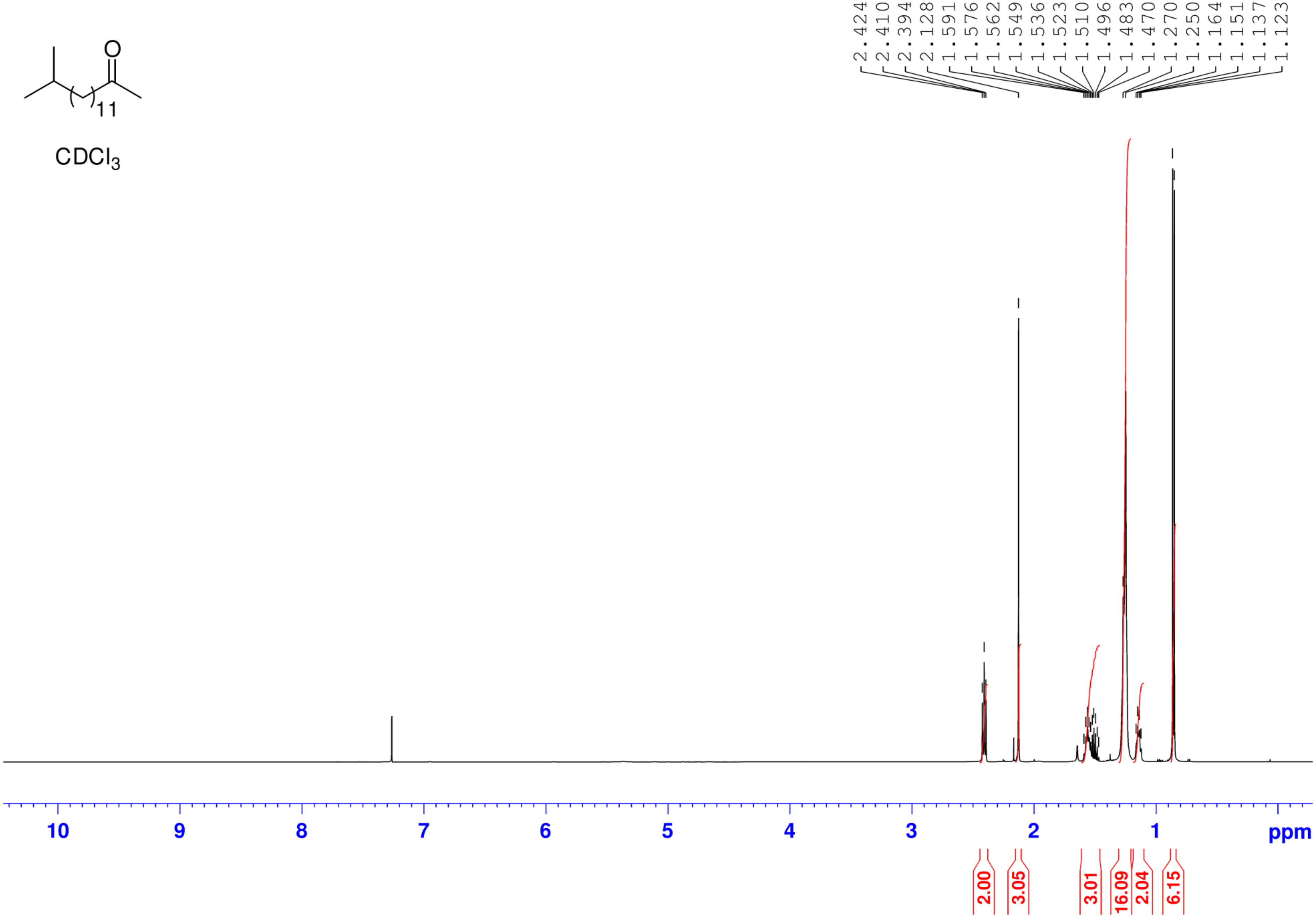

^{13}C NMR (125 MHz, $CDCl_3$) – 14-Methylpentadecan-2-one (**33**)

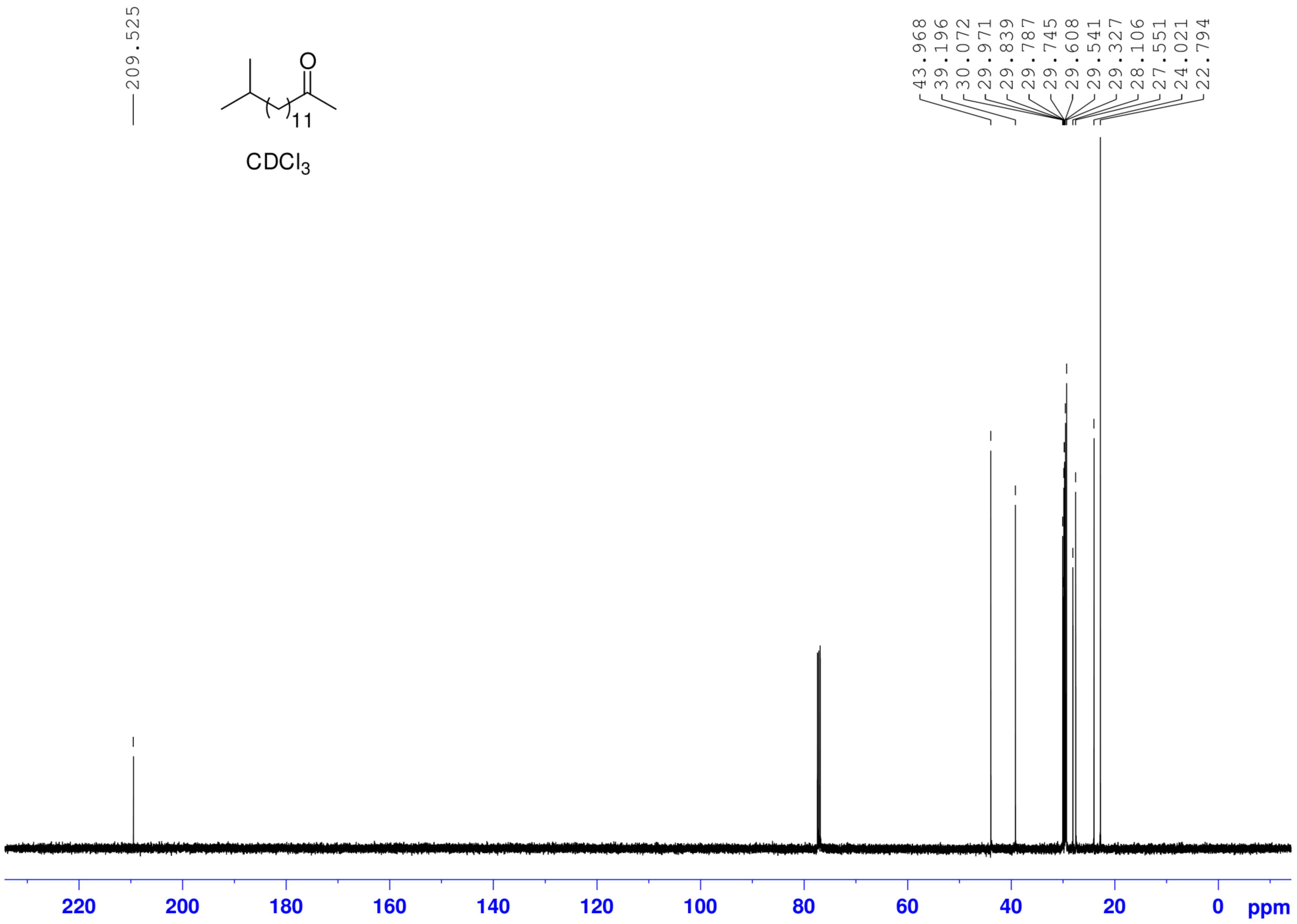

Supplement: File 2 — NMR spectra. [file Beilstein_J_Org_Chem-09-1807-s002.pdf]
